# Iodide Anion Enables a Reductive Cross‐Electrophile Coupling for Preparing Tertiary Amines

**DOI:** 10.1002/anie.202409688

**Published:** 2024-11-29

**Authors:** Miran Lemmerer, Veronica Tona, David Just, Miloš Vavrík, Boris Maryasin, Giovanni Di Mauro, Andreas B. zur Bonsen, Daniel Kaiser, Nuno Maulide

**Affiliations:** ^1^ Institute of Organic Chemistry University of Vienna Währinger Straße 38 1090 Vienna Austria; ^2^ Institute of Theoretical Chemistry University of Vienna Währinger Straße 17 1090 Vienna Austria

**Keywords:** iminium ion, cross coupling, radicals, iodide, reaction mechanisms

## Abstract

The reducing power of iodide anion is an underexplored property that can be used for the cross‐electrophile coupling of organic molecules. Herein we harness this trait for the preparation of tertiary amines through the combination of two simple reagents: an electrophilic‐carbon precursor and an iminium iodide in a dual role – both as nitrogen‐containing building block and as reducing agent. The underlying mechanism of this new C−C bond‐formation paradigm is explored through a combination of experiment and quantum chemical calculations.

## Graphical abstract

We describe a cross‐electrophile coupling of iminium ions with a range of electrophilic substrates to prepare tertiary amines. This reaction is mediated by the underexplored single‐electron reducing power of the iodide counter anion, which is investigated both experimentally and through quantum chemical calculations.

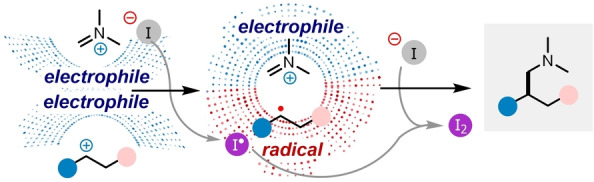

Compounds containing a carbon–iodine bond are valuable building blocks in synthetic chemistry. Indeed, the C−I bond enables a broad palette of reactivity, be it in one‐electron or two‐electron manifolds. In its elemental form (I_2_) and in higher oxidation states (I(III) and I(V)), iodine typically assumes the role of an oxidizing agent,^[1]^ while, equally as interestingly, albeit perhaps less synthetically versatile, this element's ability to act as a reducing agent has also been reported.^[2]^

In particular, the reducing chemistry of iodine (iodide) appears to be largely confined to hydrodefunctionalization reactions on specific families of carbon–heteroatom single bonds and carbon–carbon multiple bonds (Scheme 1A). Such processes typically proceed either through single‐electron reduction followed by proton‐coupled electron transfer (PCET, top) or nucleophilic deiodination (bottom) pathways.[^2^, ^3^] Additionally, the homolytic cleavage of certain alkyl C−I bonds by simple heating in the presence of iodide offers an attractive source of C‐centered radicals, and this has been deployed to initiate radical polymerization.^[4]^

**Scheme 1:**
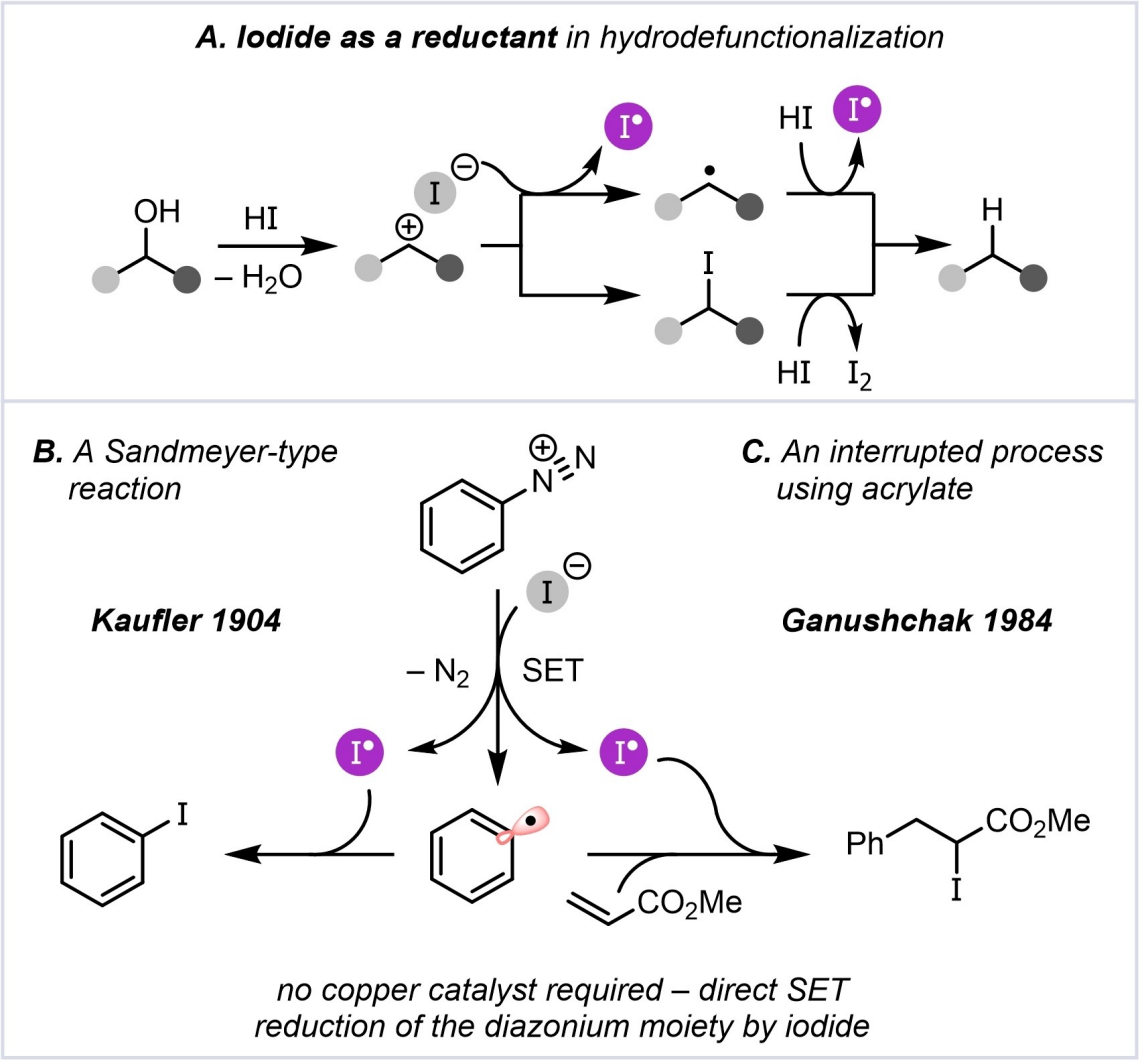
Reported classes of transformations in which iodide acts as a reductant and atom donor.

Interesting applications of iodide in the context of reductive transformations include the seminal iodination of aryldiazonium salts, reported by Kaufler in the early 1900’s, employing only potassium iodide (Scheme 1B).^[5]^ The fact that, in contrast to the venerable Sandmeyer reaction,[^6^, ^7^] this transformation does not require a copper catalyst can be ascribed to direct single‐electron transfer (SET) reduction of the diazonium moiety by iodide, leading to the formation of an aryl radical, nitrogen, and an unpaired iodine atom.^[8]^ The interruption of this process with a radical acceptor, reacting with the aryl radical before being trapped by the iodine atom, was achieved in 1984 by Ganushchak *et* *al*. using acrylate (Scheme 1C).[^9^, ^10^]

This last example would appear to be an early instance of a cross‐electrophile coupling (XEC), with iodide serving as the reducing agent, enabling the otherwise polarity‐mismatched coupling of a diazonium salt and a Michael acceptor. General strategies for XEC rely on the selective donation of either one electron or two electrons to one of the electrophilic partners – effectively a reduction with reversal of polarity.^[11]^

Naturally, these transformations face the pervasive challenge of chemoselectivity and are prone to either undesired homo‐coupling, or the formation of statistical mixtures of products.^[11]^ This issue is often addressed by the use of metal catalysts able to discriminate between the substrates through the recognition of electronic or steric differences.^[12]^ Given the success of XEC chemistry, it is surprising that the deployment of iodide as reducing enabler has not been exploited in valuable contexts.

In our previous work towards redox‐neutral amine synthesis by hydride transfer, Eschenmoser's salt (**1**) proved to be an ideal reagent for a two‐electron pathway towards linear amines (Scheme 2A). This hydroaminomethylenation selectively delivers linear products through a process initiated by a Prins‐type addition, followed by 1,5‐hydride transfer, whereby one of the amine's substituents undergoes oxidation concomitantly with reduction of the initially formed carbocation.[^13^, ^14^] Surprisingly, when combining styrene (**2 a**) and Eschenmoser's salt (**1**) in TFA – instead of HFIP – we observed formation of a different hydroaminomethylenation product **3 a** in 64 % yield (expected: **4 a**, Scheme 2B). Indeed, not only was a branched product formed, but the amino‐moiety had remained dimethylated (in contrast to the monomethylated products formed in the redox‐neutral, hydride‐transfer pathway of Scheme 2A).

**Scheme 2:**
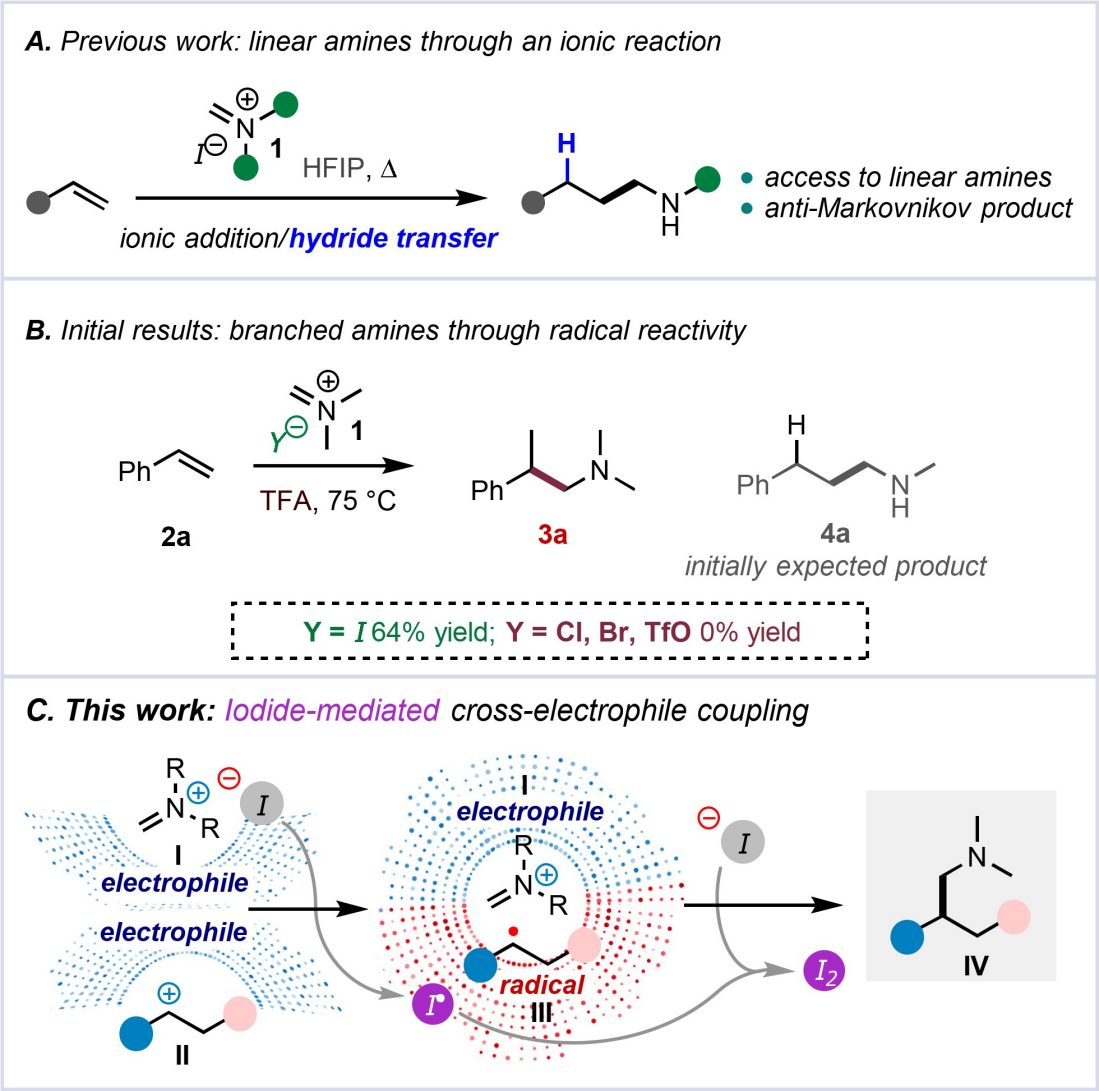
Discovery of the reductive ability of iodide to mediate a cross‐electrophile coupling between alkenes and iminium ions to generate branched amines.

Further experimentation (Scheme 2B) revealed this to be exclusive for Eschenmoser's salt (bearing an iodide counter anion), as the corresponding chloride, bromide or triflate returned no product. This result invoked the intervention of one‐electron mechanistic manifolds and triggered the thought that reagent **1**, containing both an electrophilic species and an iodide counterion, could constitute a unique reductive enabler for electrophile‐electrophile bond formation. The specificity of this chemistry for iodide also contrasts with prior examples of reductive iodide couplings, where bromide was also a capable promoter.^[4]^ Our hypothesis regarding the observed outcome is shown in Scheme 2C: We initially believed that iodide might have reduced the cation **II** (resulting from protonation of the alkene), forming radical species **III**, and opening an avenue to C−C bond formation with the iminium ion **I** (Eschenmoser's salt).^[15]^

Eager to demonstrate generality of this chemistry, we also realized that, besides undesired recombination with iodide – the ultimate potential consequences of which were initially unclear – and uncertainties surrounding SET efficiency, the background ionic hydroaminomethylenation (whenever alkenes would be used as precursors to **II**)[^13^, ^14^] appeared as a noteworthy hurdle. In this manuscript, we describe a family of cross‐electrophile couplings for the synthesis of amines, relying on iodide as the sole reductant.

Initially aware of the potential for ion‐pair collapse between cation **II** and either iodide or trifluoroacetate, we aimed to probe whether benzylic iodides or related structures could themselves serve as suitable substrates. Indeed, when subjecting iodide **5 a** to a solution of **1** in trifluoroacetic acid, we were pleased to observe the formation of homobenzylic amine **3 a** in excellent yield (90 %, Scheme 3A).^[16]^ Moreover, investigation of related potential starting materials revealed benzylic chlorides, bromides, and even alcohols to be suitable partners for this coupling (Scheme 3B, see the Supporting Information for additional information).

**Scheme 3:**
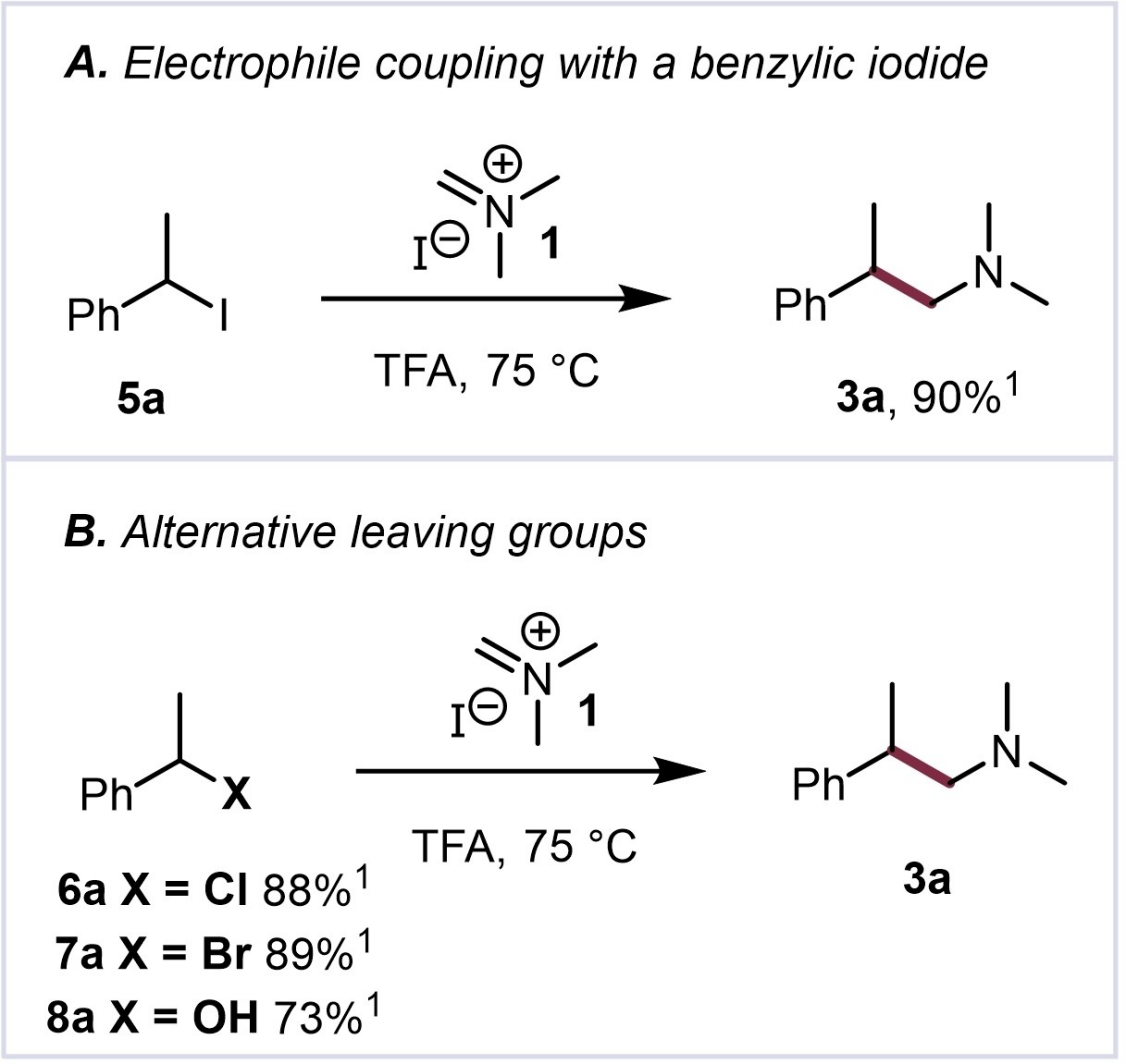
Initial screening of potential substrates for this iodide‐mediated cross‐electrophile coupling. ^1^NMR yield using 1,3,5‐trimethylbenzene as an internal standard.

Following the finding that a range of different electrophile precursors can react with Eschenmoser's salt **1**, converging to the same aminomethylenated products, we directed our attention to the exploration of the scope of the reaction with styrenes (**2**), benzyl halides (**5–7**), and benzyl alcohols (**8**) (Scheme 4).

**Scheme 4:**
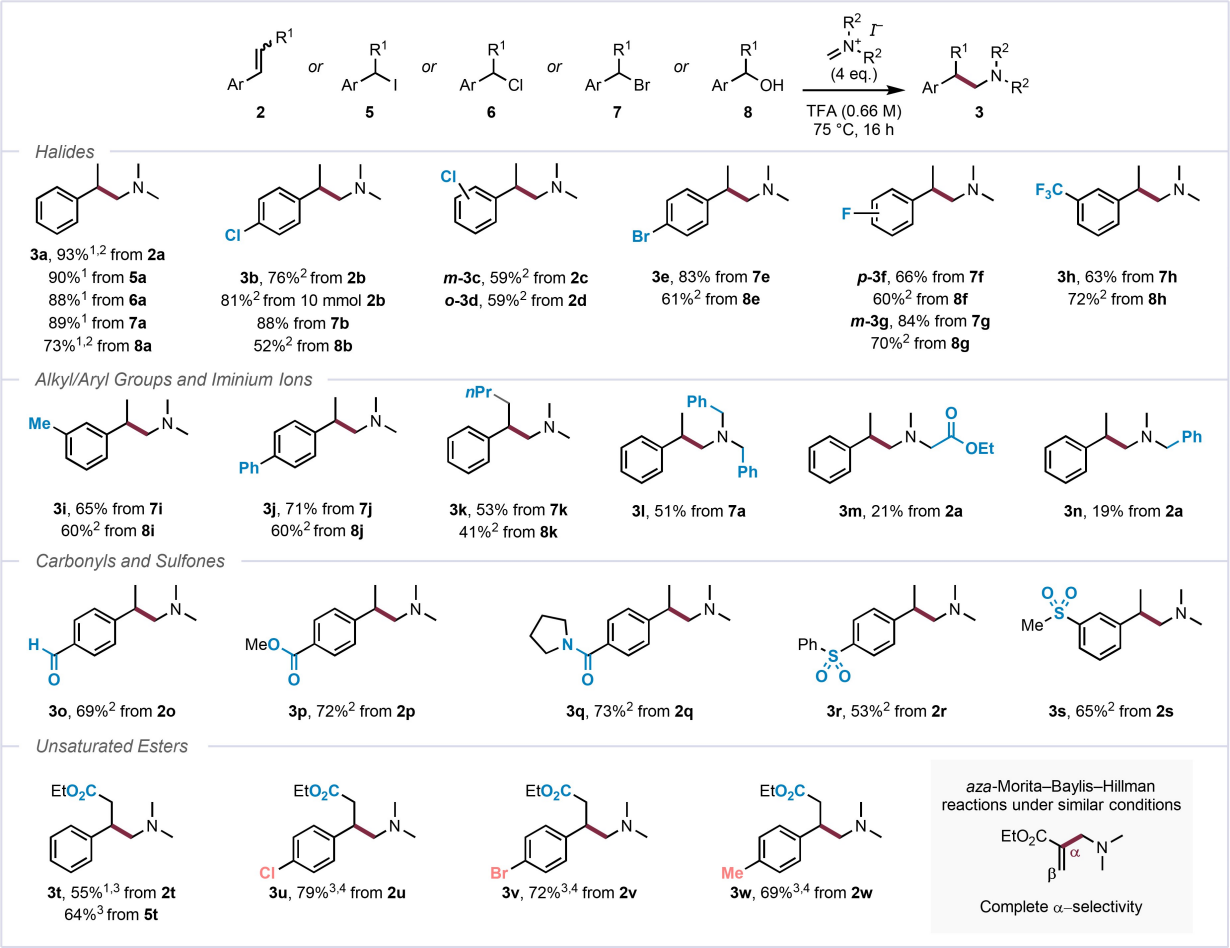
Substrate scope of the reaction of benzyl halides, benzyl alcohols, and styrenes. Unless otherwise stated, all reactions were performed on a 0.2 mmol scale at a 0.66 M concentration of **2**–**8** under argon atmosphere. ^1^NMR yield using 1,3,5‐trimethylbenzene as an internal standard. ^2^Reaction run at 1.2 M concentration. ^3^Reaction run at 1.2 M concentration and with 8 eq. Eschenmoser's salt. ^4^Based on recovered starting material; see the Supporting Information for details. TFA, trifluoroacetic acid.

It is noteworthy that, following some optimization of the reaction of styrene (see the Supporting Information for details), product **3 a** was formed in almost identical yield from any of the suitable starting materials, with only the use of benzyl alcohol **8 a** leading to a slightly diminished yield (average yield 87 %). Pleasingly, variation of the substitution pattern on the arene had little effect and, regardless of the employed substrate class, the reaction proceeded with good to high yields in the presence of aromatic halides (forming products **3 b–g**, 52–88 %) and electron‐withdrawing substituents (**3 h**, 63–72 %). Alkyl (**3 i**, 60–65 %) and aryl (**3 j**, 60–71 %) groups attached to the arene were also tolerated well. Product **3 k**, bearing a longer aliphatic chain and derived either from benzyl bromide **7 k** or benzyl alcohol **8 k** (53 % and 41 % yield, respectively) showed the ability of this method to yield products that go beyond methyl‐substitution of the α‐position.^[17]^ Dibenzylamine‐containing product **3 l** was synthesized from the corresponding *N,N*‐dibenzyl methylene iminium iodide and benzyl bromide **7 l** in moderate yield (51 %), and **3 m** and **3 n** were prepared in low yields from sarcosine ethyl ester‐ and benzylmethylamine‐derived iminium iodides, respectively. These results show that this reactivity is not limited to Eschenmoser's salt.^[18]^ Various other arene substituents were also tolerated, with carbonyl groups such as an aldehyde (**3 o**, 69 %), an ester (**3 p**, 72 %), and an amide (**3 q**, 73 %), as well as sulfones in different positions (**3 r** and **3 s**, 53 % and 65 % yield, respectively) remaining untouched. It is worth noting that, while the desired products can largely be synthesized through reductive amination of the respective α‐branched aldehydes, synthesis of such aldehydes is often laborious, particularly in the presence of additional functional groups (e.g., the putative dialdehyde precursor to **3 o**). In contrast, the styrenes employed herein are considerably more readily accessible substrates, leading to a streamlined protocol.

Intriguingly, cinnamic esters are also capable of undergoing this reaction (**3 t**, 55–64 %), with halides (**3 u** and **3 v**, 79 % and 72 %, respectively) and an alkyl group (**3 w**, 69 %) once more not showing any negative impact on the reaction outcome compared to the unsubstituted congener **3 t**. These entries further highlight the unusual nature of this transformation, in particular as similar conditions have been reported to lead to aza‐Morita–Baylis–Hillman‐type reactivity, forming a C−C bond at the α‐ rather than the β‐position (Scheme 4, bottom right).^[19]^ In contrast to the results presented in Scheme 4, however, a variety of other potential substrates, ranging from prenyl alcohol to bromodiphenylmethane, were found not to form the desired products (see the Supporting Information for a complete list of surveyed substrates).

Mechanistic investigations first relied on experimental approaches and revealed interesting trends (Scheme 5). Triply deuterated product **3 a–d3** was obtained when the reaction was performed in deuterated trifluoroacetic acid using styrene (**2 a**) as the substrate, suggesting reversible protonation (Scheme 5A). This is additionally an interesting and cost‐effective method for the introduction of a CD_3_ substituent.

**Scheme 5:**
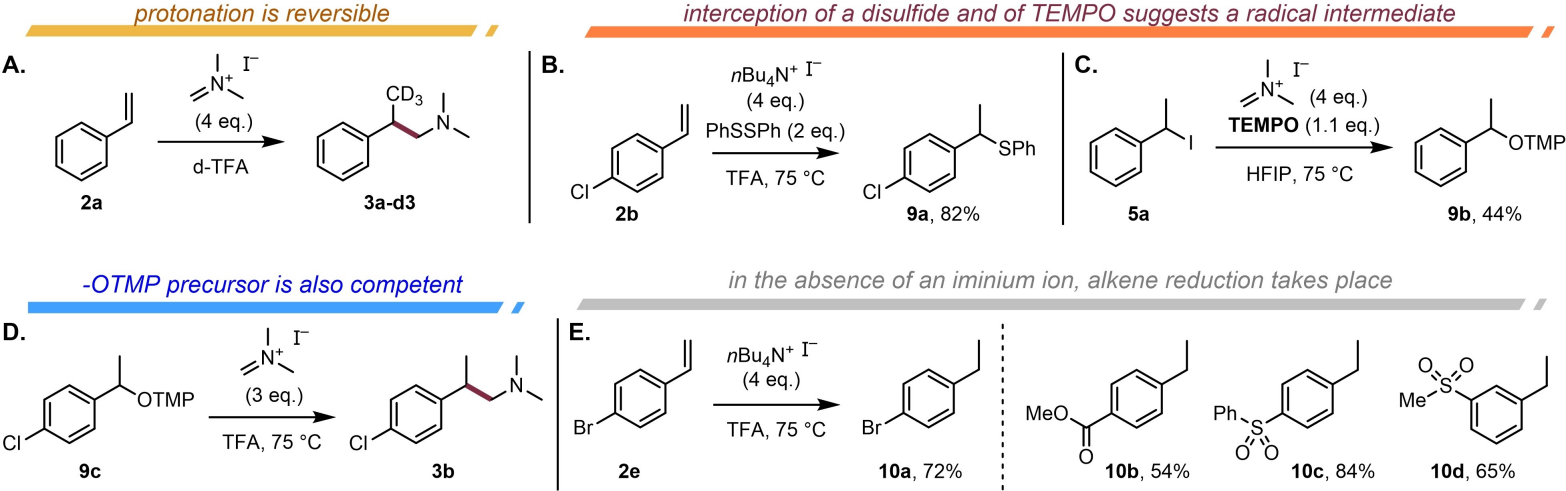
Mechanistic experiments. A) The investigated reaction in d‐TFA shows reversible protonation/deuteration. B) Reaction with diphenyl disulfide leads to sulfide formation. C) Capture by TEMPO suggests a radical intermediate. See the Supporting Information for details. D) An OTMP‐precursor also delivers the coupling product under the reaction conditions. E) Formal reduction of styrenes using iodide.

In the absence of the iminium salt, the addition of a disulfide and an iodide source led to the smooth formation of sulfide **9 a** (Scheme 5B). In contrast to this outcome, the same reaction does not proceed when the iodide is replaced by a bromide, suggesting that a radical process is at play (see the Supporting Information for details).^[20]^

Interestingly, when the iodide **5 a** was employed in the presence of TEMPO, the covalent TEMPO adduct **9 b** was isolated in 44 % yield (Scheme 5C, see the Supporting Information for further details).^[21]^ Both experiments strongly suggest the intermediacy of a benzylic radical. Further support of the hypothesized radical/iminium coupling was obtained when –OTMP ether **9 c** was subjected to the reaction conditions with Eschenmoser's salt, resulting in the same reaction product (**3 b**) (Scheme 5D).

Notably, in the absence of the electrophilic iminium ion, we observed alkene reduction (Scheme 5E). Treatment of styrenes with an iodide source bearing a spectator counter cation (*n*Bu_4_N^+^) delivered the fully saturated products **10 a–10 d** in good yields. However, this process must be considerably slower than the otherwise observed C−C coupling, given the efficiency of formation of products **3** when the iminium partner is present.[^2^, ^22^] The experimentally observed formation of iodine (I_2_) as a byproduct (see the Supporting Information for details), as well as the experimental results described in Scheme 5, suggest a process in which all electrophilic precursors transiently converge to an iodinated hydrocarbon intermediate, and it is the (homolytic) cleavage of such a species, forming a secondary benzylic radical and iodine radical, that results in a formal SET event.

Computational analysis (Scheme 6) of the possible radical intermediates indicated that the association of the iodine radical and an iodide (from Eschenmoser's salt) leads to the formation of an anionic diiodide radical species in an exergonic process (I^−^ + I^⋅^→I_2_^⋅−^, Δ*G*_348_=−9.6 kcal mol^−1^).^[23]^

**Scheme 6:**
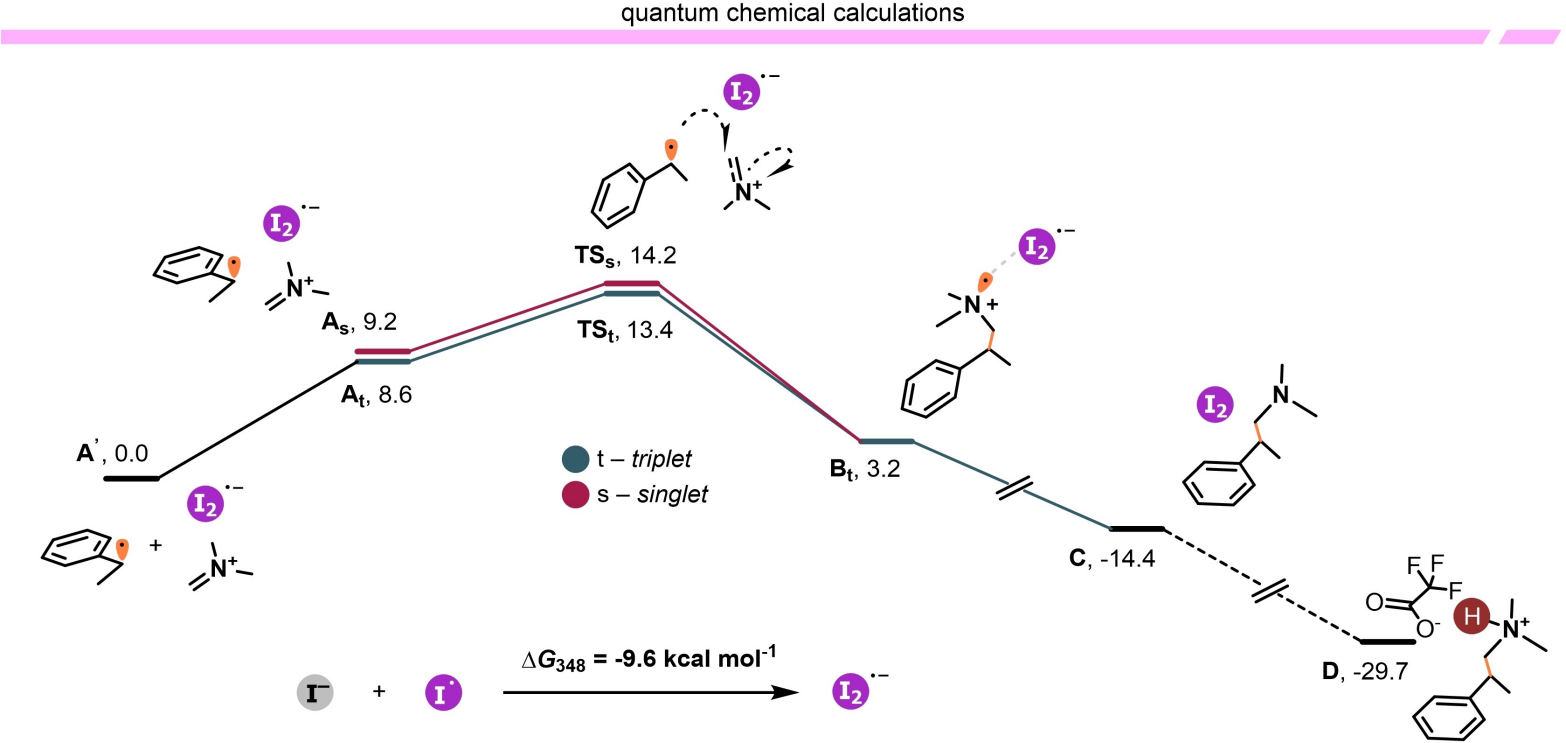
Computed reaction profile (M06‐2X‐D3/def2‐TZVP, Δ*G*_348_,_TFA_). The sum of the energies of the isolated radical species **A′** is set as the reference (0.0 kcal/mol). See the Supporting Information for computational details.

Therefore, the neutral complex formed by the association of this radical with the counterion, combined with the benzylic radical considered separately, was selected as the reference point (**A’**, 0.0 kcal/mol) for the computational investigation of the mechanism. Initially, the ternary diradical complex **A**, which allows for system analysis in the triplet (green path) and singlet state (red path), is formed with a characteristic entropic price of 8.6 kcal mol^−1^. Radical nucleophilic addition of the benzylic radical onto the iminium ion was found to be a favored process (Δ*G*_348_(**A_t_** →**B_t_**)=−5.4 kcal mol^−1^), leading to the formation of intermediate **B** via transition state **TS** (Δ*G* ^≠^_348_(**A_t_** →**B_t_**)=4.8 kcal mol^−1^).

It should be noted that, for intermediate **A** and the transition state **TS**, the triplet state is computed to be ~0.7 kcal mol^−1^ more stable than the singlet state. This reduced singlet‐triplet gap emphasizes the strong diradical character of the found stationary points.

Aminium radical cations (**B**) are known single‐electron oxidants,[^24^, ^25^] capable of abstracting an electron from the weakly reducing diiodide radical, thereby forming the neutral intermediate **C** in a highly exergonic process (Δ*G*_348_(**A’**→**C**)=−14.4 kcal mol^−1^). Under the reaction conditions, amine **C**, in turn, is swiftly protonated by a solvent molecule, yielding **D** as the final product (Δ*G*_348_(**A’**→**D**)=−29.7 kcal mol^−1^).

Based on the computational and experimental data, we propose the mechanism depicted in Scheme 7, for the case of an alkene partner. Rapid and reversible protonation leads to the formation of TFA‐adduct **11** and subsequently the corresponding benzylic iodide (**5 a**), a species that was additionally observed by ^1^H NMR analysis at incomplete conversions.

**Scheme 7:**
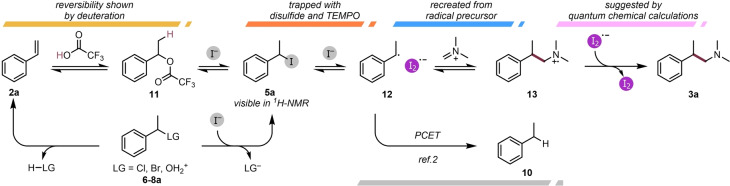
The proposed reaction mechanism based on experimental and computational results.

Other precursors of the investigated reaction, such as halides or alcohols, can be converted to the pivotal iodide via direct substitution or intermittent elimination to the styrene (see the Supporting Information for details). Interestingly, the iodide intermediate is also observed in the case of unsaturated esters **3 t–w**; here, it is likely formed by halo‐Michael addition. Homolytic cleavage of the C−I bond, facilitated by iodide, is proposed to lead to the formation of the aforementioned diiodide radical complex and a radical species (**12**) capable of adding to the iminium ion to form a new carbon−carbon bond, generating radical cation **13**. Single‐electron transfer from the diiodide radical complex to this resulting aminium radical cation forms the reduced tertiary amine and generates I_2_ as the only stoichiometric byproduct (crystals of which were occasionally observed in the headspace of the reaction mixture). As a slow side reaction, inconsequential in the presence of iminium ions, the generation of formally hydrogenated product **10** from stabilized radical **12** could potentially proceed through a proton‐coupled electron transfer mechanism mediated by HI.^[2]^

In summary, we have developed a metal‐free cross‐electrophile coupling of iminium iodides with carbocation precursors such as halides, alcohols, and alkenes, a method which utilizes the underexplored redox capability of iodide. Through mechanistic investigations, we highlight the unique role of this species in allowing the union of two electrophiles in an amine synthesis that does not require stoichiometric HAT agents, is regiocomplementary to ionic chemistry and provides iodine as single byproduct. We anticipate that this report might open a new direction for iodide as a readily available enabler in the context of reductive C−C bond‐forming reactions.

## Supporting Information

The authors have cited additional references within the Supporting Information.[^26^, ^27^, ^28^, ^29^, ^30^, ^31^, ^32^, ^33^, ^34^, ^35^, ^36^, ^37^, ^38^, ^39^, ^40^, ^41^, ^42^, ^43^, ^44^, ^45^, ^46^, ^47^, ^48^, ^49^, ^50^, ^51^, ^52^]

## Conflict of Interests

The authors declare no conflict of interest.

## Supporting information

As a service to our authors and readers, this journal provides supporting information supplied by the authors. Such materials are peer reviewed and may be re‐organized for online delivery, but are not copy‐edited or typeset. Technical support issues arising from supporting information (other than missing files) should be addressed to the authors.

Supporting Information

## Data Availability

The data that support the findings of this study are available in the supplementary material of this article.
