# Supplementary material for: Iodide Anion Enables a Reductive Cross‐Electrophile Coupling for Preparing Tertiary Amines
Source: Angew Chem Int Ed Engl. 2024 Nov 29;64(2):e202409688. doi: 10.1002/anie.202409688 (PMC11720395; doi:10.1002/anie.202409688)
Supplement: Supplementary file 1 — Supporting Information [file ANIE-64-e202409688-s001.pdf]

Angewandte Chemie
Eine Zeitschrift der Gesellschaft Deutscher Chemiker

Supporting Information

Iodide Anion Enables a Reductive Cross-Electrophile Coupling for Preparing Tertiary Amines

*M. Lemmerer, V. Tona, D. Just, M. Vavrík, B. Maryasin, G. Di Mauro, A. B. zur Bonsen, D. Kaiser, N. Maulide**

Contents

Materials and Methods 1
Optimization for hydroaminomethylation of styrenes and benzylic substrates 2
Optimization for hydroaminomethylation of cinnamates 3
Preparation of starting materials and their characterization 4
Product synthesis and characterization 6
Reduction of styrenes by tetrabutylammonium iodide 17
Iminium iodides derived from other amines 19
Investigation of other potential substrates 20
Mechanistic investigation 21
Formation of I_2 during the reaction 26
NMR Spectra 28
Computational details 63
References 69

Materials and Methods

General information

Unless otherwise stated, all glassware was flame-dried before use and all reactions were performed under an atmosphere of argon. All reagents were used as received from commercial suppliers unless otherwise stated. Eschenmoser's salt was purchased from TCI, stored in smaller containers (to avoid repeated air contact of larger batches) when received (ca. 5 g each) and used as such. Reaction progress was monitored by thin layer chromatography (TLC) performed on aluminium plates coated with silica gel F254 with 0.2 mm thickness. Chromatograms were visualized by fluorescence quenching with UV light at 254 nm or by staining using potassium permanganate. Flash column chromatography was performed on an Isolera 4 medium pressure chromatography system (Biotage) using silica gel 60 (230-400 mesh, Merck and co.). Neat infrared spectra were recorded using a Perkin-Elmer Spectrum 100 FT-IR spectrometer. Wavenumbers (ν_{max}) are reported in cm^{-1}. Mass spectra were obtained using a Bruker maXis UHR-TOF (Qq-TOF) spectrometer, using electrospray ionization (ESI). All ^{1}H NMR, ^{13}C NMR and ^{19}F NMR spectra were recorded using a Bruker AV-400, AV-500, AV-600 or AV-700 spectrometer at 300K. Chemical shifts are given in parts per million (ppm, δ), referenced to the solvent peak of $CDCl_3$, defined at δ = 7.26 ppm (^{1}H NMR) and δ = 77.16 (^{13}C NMR). Coupling constants are quoted in Hz (J). ^{1}H NMR splitting patterns are designated as singlet (s), doublet (d), triplet (t), quartet (q) as they appeared in the spectrum. If the appearance of a signal differs from the expected splitting pattern, the observed pattern is designated as apparent (app). Splitting patterns that could not be interpreted or easily visualized are designated as multiplet (m) or broad (br).

Optimization for hydroaminomethylation of styrenes and benzylic substrates

Optimization was performed under argon in an aluminium foil-covered Schlenk flask, unless otherwise stated. Since neither light nor air affected the yield, the reaction scope was investigated in capped oven-dried vials under air (see general procedures below).

Table S1. Optimization on styrene

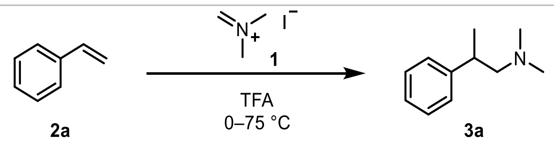

entry	Equiv. **1**	temp (°C)	TFA (M)	time (h)	notes	NMR yield
**1**	4	75	0.6	16	under light	76%
**2**	4	75	0.6	16	under air	73%
**3**	4	r.t.	0.6	16	-	28%
**4**	4	75	0.6	1	-	53%
**5**	4	75	0.6	2	-	75%
**6**	1	75	0.6	2	-	15%
**7**	2	75	0.6	2	-	37%
**8**	4	75	1.2	2	-	93%
**9**	4	75	4 equiv.	2	-	40%
**10**	4	75	4 equiv. + DCE (0.6)	2	-	<15%

Table S2. Optimization on 1-bromo-1-phenyl ethane

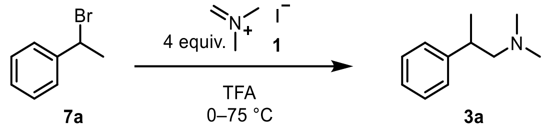

entry	Equiv. TFA	temp (°C)	c (M)	time (h)	co-solvent	NMR yield
**1**	22.4	75	0.6	16	-	93%
**2**	1	75	0.6	16	DCE	39%
**3**	0	75	0.6	16	DCE	15%
**4**	22.4	75	0.6	2	-	77%
**5**	11.2	75	1.2	16	-	88%
**6**	11.2	75	0.6	16	DCE	89%
**7**	11.2	50	0.6	16	DCE	-
**8**	11.2	60	0.6	16	DCE	60%
**9**	1 TfOH	75	0.6	16	DCE	37%
**10**	0	75	0.6	16	HFIP	62%

Optimization for hydroaminomethylation of cinnamates

Table S3. Optimization on ethyl cinnamate

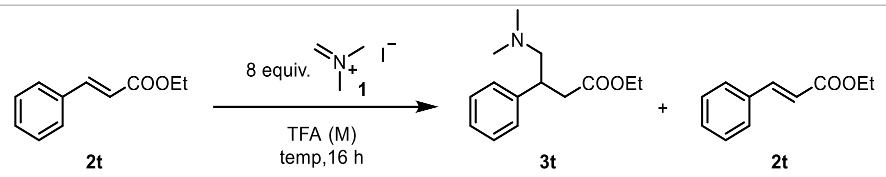

**entry**	c (M)	temp (°C)	other deviations	NMR yield **3t** (%)	NMR yield **2t** (%)
**1**	0.6	75	-	38	
**2**	1.8	75	-	33	50
**3**	1.2	75	4 equiv. **1**	34	65
**4**	1.2	75	6 equiv. **1**	54	38
**5**	1.2	75	8 equiv. **1**	55	36
**6**	1.2	75	degassed TFA	43	48
**7**	1.2	75	oxygen atmosphere	n.d.	80
**8**	1.2	75	drop of water	37	39
**9**	1.2	100	-	41	35
**10**	1.2	75	4 equal portions of **1** after 1 hour each	34	66
**11**	1.2	75	1 equiv. I_2	40	60
**12**	1.2	75	**1** washed with Et_2O prior to use (contains sulfolane from production)	50	45
**13**	1.2	75	Additional 4 equiv. of TBAI	17	75
**14**	1.2	75	Additional 0.4 equiv. of **2t**	63	74
**15**	1.2	75	subjected to a second cycle	53	20
**16**	1.2	75	AcOH (1.2 M)	n.d.	85
**17**	1.2	75	TCA (1.2 M)	17	77
**18**	1.2	75	H_2SO_4 (1.2 M)	n.d	n.d.

Preparation of starting materials and their characterization

Styrenes

The styrene **2p** was synthesized in methanol according to the procedure described by Bezerra França *et al.*[26] The obtained spectroscopic data match those reported.

The styrenes **2o** and **2s** were synthesized according to the procedure described by Scheidt *et al.*[27] The obtained spectroscopic data match those reported.

Pyrrolidin-1-yl(4-vinylphenyl)methanone (2q)

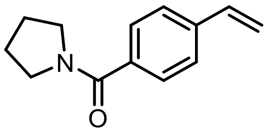

To 4-vinylbenzoic acid (148 mg, 1.00 mmol, 1.00 equiv.) was added pyrrolidine (71.1 mg, 1.00 mmol, 1.00 equiv.), Et_3N (101 mg, 1.00 mmol, 1.00 equiv), HOBt (135 mg, 1.00 mmol, 1.00 equiv.), EDCI (192 mg, 1.00 mmol, 1.00 equiv.) in 5 mL DCM (0.2 M). The reaction was stirred for 16 h, before being diluted with EtOAc and washed sequentially with 1 M HCl (aq.), saturated $NaHCO_3$ (aq.), and brine. The organic phase was dried over $MgSO_4$, and, after filtration, the solvent was removed under reduced pressure to afford compound **2q** in quantitative (201 mg) yield as a yellow solid.

^{1}H NMR (400 MHz, $CDCl_3$) δ 7.49 (d, *J* = 8.3 Hz, 2H), 7.42 (d, *J* = 8.3 Hz, 2H), 6.72 (dd, *J* = 17.5, 10.9 Hz, 1H), 5.79 (d, *J* = 17.5 Hz, 1H), 5.30 (d, *J* = 10.9 Hz, 1H), 3.64 (t, *J* = 6.9 Hz, 2H), 3.43 (t, *J* = 6.9 Hz, 2H), 2.00–1.81 (m, 4H); **^{13}C NMR (100 MHz, $CDCl_3$) δ** 169.5, 139.1, 136.6, 136.3, 127.6 (2C), 126.1 (2C), 115.2, 49.7, 46.3, 26.5, 24.5; **IR (neat) ν_{max}:** 2970, 2873, 1608, 1413, 907, 851, 775, 726; **HRMS (ESI$^+$):** exact mass calculated for $[M+Na]^+$ ($C_{13}H_{15}NNaO^+$) requires 224.1046 *m/z*, found 224.1043 *m/z*.

1-(Phenylsulfonyl)-4-vinylbenzene (2r)

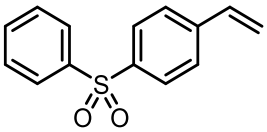

This procedure was adapted from Wang *et al.*[28] To a Schlenk flask was added 1-bromo-4-(phenylsulfonyl)benzene (594 mg, 2.00 mmol, 1.00 equiv.), dicyclohexyl(2′,6′-dimethoxy[1,1′-biphenyl]-2-yl)phosphane (SPhos, 82.1 mg, 0.20 mmol, 0.10 equiv.), anhydrous potassium phosphate (1.27 g, 6 mmol, 3.00 equiv.), 4,4,5,5-tetramethyl-2-vinyl-1,3,2-dioxaborolane (509 µL, 3.00 mmol, 1.50 equiv.), 15 mL dioxane and 3 mL H_2O (0.11 M). The mixture was degassed by bubbling argon through the solution. Then, palladium acetate (22.5 mg, 0.10 mmol, 0.05 equiv.) was added and the mixture heated to 80 °C for 14 h. After cooling to room temperature, the mixture was diluted with 10 mL EtOAc and filtered through a short silica pad. The solution was washed with brine, dried over $MgSO_4$, filtered, and the filtrate was concentrated under reduced pressure. Purification by column chromatography on silica gel (0% to 40% EtOAc in heptane) afforded compound **2r** in 99% (486 mg) yield as a yellow solid.

^{1}H NMR (400 MHz, CDCl$_3$): **δ** 7.97 – 7.87 (m, 4H), 7.59 – 7.47 (m, 5H), 6.71 (dd, *J* = 17.5, 10.8 Hz, 1H), 5.85 (d, *J* = 17.5 Hz, 1H), 5.42 (d, *J* = 10.8 Hz, 1H); **^{13}C NMR (176 MHz, CDCl$_3$): δ** 142.5, 141.9, 140.5, 135.4, 133.3, 129.4 (2C), 128.2 (2C), 127.7 (2C), 127.1 (2C), 117.9; **IR (neat) ν$_{max}$:** 1594, 1446, 1308, 1156, 1105, 924, 846, 719, 648; **HRMS (ESI$^+$):** exact mass calculated for $[M+Na]^+$ ($C_{14}H_{12}NaO_2S^+$) requires *m/z* 267.0450, found *m/z* 267.0447.

Ethyl cinnamates **2u**–**2w** were synthesized from the corresponding carboxylic acids and thionyl chloride in ethanol. The obtained spectroscopic data match those reported.[26]

Benzylic halides and alcohols

Non-commercial benzylic halides were synthesized from the corresponding alcohols using the procedure described by Holz *et al.*[29] Non-commercial benzylic alcohols were synthesized from the corresponding aldehydes using the procedure described by Polidano *et al.*[30] The obtained spectroscopic data match those previously reported.

Ethyl 3-iodo-3-phenylpropanoate (5t)

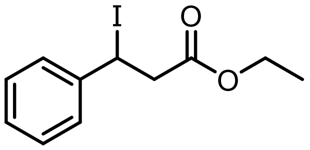

The procedure was adapted from Das *et al.*[31] To a solution of ethyl cinnamate (168 µL, 1.0 mmol, 1.00 equiv.) and polymethylhydrosiloxane (89.7 µL, 1.50 mmol, 1.50 equiv.) in chloroform (10 mL; 0.1 M), iodine (254 mg, 1 mmol, 1.00 equiv.) was added. The reaction was allowed to stir at room temperature for 2 h and monitored by TLC. On completion, the solvent was evaporated under vacuum and the reaction mixture was dissolved in DCM and filtered through a plug of silica gel to yield the iodinated product **5r** in pure form in 88% (267 mg) yield.

^{1}H NMR (400 MHz, CDCl$_3$) δ 7.44 – 7.37 (m, 2H), 7.33 – 7.22 (m, 3H), 5.54 (dd, *J* = 8.7, 7.0 Hz, 1H), 4.21 – 4.05 (m, 2H), 3.40 (dd, *J* = 16.3, 8.8 Hz, 1H), 3.29 (dd, *J* = 16.3, 6.9 Hz, 1H), 1.22 (t, *J* = 7.1 Hz, 3H); **^{13}C NMR (101 MHz, CDCl$_3$) δ** 170.1, 143.1, 129.0 (2C), 128.4 (2C), 127.1, 61.2, 46.6, 23.9, 14.3; **IR (neat) ν$_{max}$:** 3086, 3063, 3031, 2980, 2931, 2908, 2871, 1736, 1494, 1454, 1393, 1376, 1321, 1299, 1265 1196, 1176, 1115, 1079, 1021, 954, 927, 911, 762; **HRMS (ESI$^+$):** exact mass calculated for $[M+Na]^+$ ($C_{11}H_{13}NO_2INa^+$) requires 326.9858 *m/z*, found 326.9852 *m/z*.

Product synthesis and characterization

Hydroaminomethylation of styrenes and aminomethylation of benzylic halides or alcohols

General Procedure A: Aminomethylation of benzylic halides

An oven-dried vial containing a stirring bar was charged with Eschenmoser's salt (**1**) (148 mg, 0.80 mmol, 4.00 equiv.), followed by addition of the benzylic halide (0.20 mmol, 1.00 equiv.) and finally trifluoroacetic acid (TFA, 0.33 mL, 22.4 equiv., 0.6 M with respect to the halide). After complete addition of TFA, the flask was sealed and placed in an oil bath at 75 °C. The reaction was vigorously stirred at this temperature for 16 h, after which it was allowed to cool to room temperature. The crude mixture was then treated with aqueous sodium hydroxide (until pH = 12) and extracted with DCM (4 x 15 mL). The combined organic phases were then dried over anhydrous K_2CO_3, filtered, and concentrated under reduced pressure. The crude product was purified by flash column chromatography on silica gel with DCM/DMA-MIX 2:1 (DMA-MIX = DCM:MeOH:NH_4OH (aq. 25%) = 90:10:1) to afford the pure product.

General Procedure B: Aminomethylation of benzylic alcohols

An oven-dried vial containing a stirring bar was charged with Eschenmoser's salt (**1**) (148 mg, 0.80 mmol, 4.00 equiv.), followed by addition of the benzylic alcohol (0.20 mmol, 1.00 equiv.) and finally trifluoroacetic acid (TFA, 0.166 mL, 11.2 equiv., 1.2 M with respect to the alcohol). After complete addition of TFA, the flask was sealed and placed in an oil bath at 75 °C. The reaction was vigorously stirred at this temperature for 12 h, after which it was allowed to cool to room temperature. The crude mixture was then treated with aqueous sodium hydroxide (until pH = 12) and extracted with DCM (4 x 15 mL). The combined organic phases were then dried over anhydrous K_2CO_3, filtered and concentrated under reduced pressure. The crude product was purified by flash column chromatography on silica gel with DCM/DMA-MIX 2:1 (DMA-MIX = DCM:MeOH:NH_4OH (aq. 25%) = 90:10:1) to afford the pure product.

General Procedure C: Hydroaminomethylation of styrenes

An oven-dried vial containing a stirring bar was charged with Eschenmoser's salt (**1**) (148 mg, 0.80 mmol, 4.00 equiv.). After this, trifluoroacetic acid (TFA, 0.33 mL, 22.4 equiv., 0.6 M with respect to the alkene) was added, followed by the styrene (0.20 mmol, 1.00 equiv.) in one portion. The flask was sealed and placed in an oil bath at 75 °C. The reaction was vigorously stirred at this temperature for 16 h, after which it was allowed to cool to room temperature. The crude mixture was then treated with aqueous sodium hydroxide (until pH = 12) and extracted with DCM (4 x 15 mL). The combined organic phases were then dried over anhydrous K_2CO_3, filtered, and concentrated under reduced pressure. The crude product was purified by flash column chromatography on silica gel with DCM /DMA-MIX 2:1 (DMA-MIX = DCM:MeOH:NH_4OH (aq. 25%) = 90:10:1) to afford the pure product.

General Prodecure D: Hydroaminomethylation of cinnamates

An oven-dried vial containing a stirring bar was charged with Eschenmoser's salt (**1**) (296 mg, 1.60 mmol, 8.00 equiv.). The ethyl cinnamate (0.20 mmol, unless stated otherwise, 1.00 equiv.) was then added, followed by trifluoroacetic acid (0.166 mL, 11.2 equiv., 1.2 M with respect to the alkene). After complete addition of TFA, the flask was sealed and placed in pre-heated oil bath and vigorously stirred at 75 °C for 16 h. Upon completion, the mixture was allowed to cool to room temperature and diluted with DCM. The mixture was then washed with 1 M aqueous sodium hydroxide (15 mL) and the aqueous phase was extracted with dichloromethane (4 x 15 mL). The combined organic phases were dried over anhydrous K_2CO_3, filtered, and concentrated under reduced pressure. The crude product

was purified by flash column chromatography on silica gel (DCM/DMA-MIX 1:0 to 2:1 gradient; DMA-MIX = DCM:MeOH:NH_4OH (aq. 25%) = 90:10:1) to afford the β-substituted γ-aminoacid ester.

_N,N_-Dimethyl-2-phenylpropan-1-amine (3a)

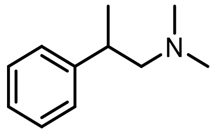

3a was prepared according to general procedures **A**, **B**, and **C** using styrene **2a**, halides **5a–7a**, and alcohol **8a**, respectively. Purification by column chromatography on silica gel (10% DMA-MIX to 75% DMA-MIX in DCM, Rf = 0.25 in 40% DMA-MIX) afforded compound **3a** in 90% (NMR yield, procedure **A** from iodide **5a**), 57% (18.7 mg, procedure **B** from alcohol **6a**), and 64% (21 mg, procedure **C** from styrene **2a**) yields as a yellow liquid.

The spectroscopic data were in accordance with those reported.[32]

^{1}H NMR (400 MHz, CDCl3) δ 7.32 – 7.28 (m, 2H), 7.22 – 7.18 (m, 3H), 2.97 – 2.84 (m, 1H), 2.42 – 2.40 (m, 2H), 2.23 (s, 6H), 1.27 (d, J = 6.9 Hz, 3H).

2-(4-Chlorophenyl)-_N,N_-dimethylpropan-1-amine (3b)

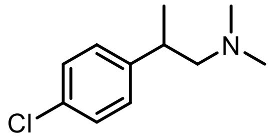

3b was prepared according to general procedures **A**, **B**, and **C** using styrene **2b**, bromide **7b**, and alcohol **8b** (at 1.2 M concentration), respectively. Purification by column chromatography on silica gel (10% DMA-MIX to 75% DMA-MIX in DCM, Rf = 0.23 in 40% DMA-MIX) afforded compound **3b** in 88% (35 mg, procedure **A**), 52% (20.5 mg, procedure **B**), and 76% (30 mg, procedure **C**; 81%, 1.60 g on a 10 mmol scale) yields as a yellow liquid.

^{1}H NMR (400 MHz, $CDCl_3$) δ 7.28 – 7.24 (m, 2H), 7.15 – 7.13 (m, 2H), 2.92 – 2.83 (m, 1H), 2.41 (dd, *J* = 12.2, 7.3 Hz, 1H), 2.37 – 2.32 (m, 1H), 2.21 (s, 6H), 1.23 (d, J = 6.9 Hz, 3H); **^{13}C NMR (100 MHz, $CDCl_3$) δ** 144.8, 131.9, 128.7 (2C), 128.6 (2C), 67.5, 46.0 (2C), 37.7, 20.4; **IR (neat) ν_{max}:** 2928, 2816, 2766, 1492, 1459, 1092, 1035, 1012, 855, 820; **HRMS (ESI$^+$):** exact mass calculated for $[M+H]^+$ ($C_{11}H_{17}NCl$) requires *m/z* 198.1044, found *m/z* 198.1044.

2-(3-Chlorophenyl)-_N,N_-dimethylpropan-1-amine (3c)

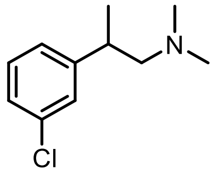

3c was prepared according to general procedure **C** using styrene **2c** at 1.2 M concentration. Purification by column chromatography on silica gel (20% DMA-MIX to 70% DMA-MIX in DCM, Rf = 0.2 in 40% DMA-MIX) afforded compound **3c** in 59% (23.3 mg) yield as a yellow liquid.

^{1}H NMR (400 MHz, CDCl$_3$) δ 7.30 –7.18 (m, 3H) 7.12 (d, *J* = 7.6 Hz, 1H), 2.96 – 2.85 (m, 1H), 2.49 – 2.35 (m, 2H), 2.25 (s, 6H), 1.27 (d, *J* = 7.0 Hz, 3H); **^{13}C NMR (150 MHz, CDCl$_3$) δ** 148.4, 134.3, 129.8, 127.4, 126.5, 125.5, 67.4, 46.0 (2C), 38.1, 20.3; **IR (neat) ν_{max}:** 2967, 2816, 2766, 1596, 1572, 1459, 1430, 1283, 1035, 781, 695; **HRMS (ESI$^+$):** exact mass calculated for $[M+H]^+$ ($C_{11}H_{17}NCl$) requires *m/z* 198.1044, found *m/z* 198.1047.

2-(2-Chlorophenyl)-*N,N*-dimethylpropan-1-amine (3d)

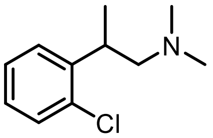

3d was prepared according to general procedure **C** using styrene **2d**. Purification by column chromatography on silica gel (20% DMA-MIX to 70% DMA-MIX in DCM, Rf = 0.2 in 40% DMA-MIX) afforded compound **3d** in 59% (23 mg) yield as a yellow liquid.

^{1}H NMR (400 MHz, CDCl$_3$) δ 7.34 (dd, *J* = 7.9, 0.9 Hz, 1H), 7.27 – 7.21 (m, 2H), 7.14 – 7.10 (m, 1H), 3.54 – 3.45 (m, 1H), 2.48 (dd, *J* = 12.1, 6.7 Hz, 1H), 2.33 (dd, *J* = 12.1, 8.4 Hz, 1H), 2.25 (s, 6H), 1.25 (d, *J* = 6.9 Hz, 3H); **^{13}C NMR (150 MHz, CDCl$_3$) δ** 143.0, 133.7, 129.6, 127.4, 127.2, 127.0, 66.1, 45.8 (2C), 33.6, 19.1; **IR (neat) ν_{max}:** 2928, 2816, 2766, 1492, 1458, 1092, 1035, 1012, 856, 820; **HRMS (ESI$^+$):** exact mass calculated for $[M+H]^+$ ($C_{11}H_{17}NCl$) requires *m/z* 198.1044, found *m/z* 198.1040.

2-(4-Bromophenyl)-*N,N*-dimethylpropan-1-amine (3e)

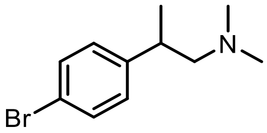

3e was prepared according to general procedures **A** and **B** using bromide **7e** and alcohol **8e**, respectively. Purification by column chromatography on silica gel (20% DMA-MIX to 50% DMA-MIX in DCM, Rf = 0.4 in 40% DMA-MIX) afforded compound **3e** in 83% (40 mg, procedure **A**) and 61% (29.4 mg, procedure **B**) yields as a yellow liquid.

^{1}H NMR (400 MHz, CDCl$_3$) δ 7.44 – 7.42 (m, 2H), 7.12 – 7.10 (m, 2H), 3.02 (app. dd, *J* = 14.3, 7.2 Hz, 1H), 2.59 (d, *J* = 7.4 Hz, 2H), 2.32 (s, 6H), 1.28 (d, *J* = 6.9 Hz, 3H); **^{13}C NMR (150 MHz, CDCl$_3$) δ** 144.3, 131.9 (2C), 129.0 (2C), 120.4, 66.7, 45.6 (2C), 37.3, 20.7; **IR (neat) ν_{max}:** 2967, 2683, 1487, 1459, 1408, 1071, 1009, 823, 749; **HRMS (ESI$^+$):** exact mass calculated for $[M+H]^+$ ($C_{11}H_{17}NBr$) requires *m/z* 242.0539, found *m/z* 242.0547.

2-(4-Fluorophenyl)-*N,N*-dimethylpropan-1-amine (3f)

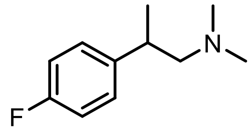

3f was prepared according to general procedure **A** and **B** using bromide **7f**, alcohol **8f**. Purification by column chromatography on silica (20% DMA-MIX to 70% DMA-MIX in DCM, Rf = 0.3 in 40% DMA-MIX) afforded compound **3f** in 66% (22 mg, procedure **A**) and 60% (21.6 mg, procedure **B**) yields as a yellow liquid.

^{1}H NMR (400 MHz, CDCl$_3$) δ 7.21 – 7.17 (m, 2H), 7.02 – 6.98 (m, 2H), 3.04 (app. dd, *J* = 14.2, 7.1 Hz, 1H), 2.57 (d, *J* = 7.5 Hz, 2H), 2.32 (s, 6H), 1.29 (d, *J* = 6.9 Hz, 3H); **^{13}C NMR (150 MHz, CDCl$_3$) δ** 161.5 (d, *J* = 243.4 Hz), 141.9, 128.5 (d, *J* = 7.7 Hz, 2C), 115.3 (d, *J* = 21.0 Hz, 2C), 67.8, 46.0 (2C), 37.5, 20.2; **^{19}F NMR (659 MHz, CDCl$_3$) δ** -117.5 (s, 1F); **IR (neat) ν_{max}:** 2935, 2870, 1634, 1567, 1453, 1315, 1222, 1065, 1013, 898, 864, 703; **HRMS (ESI$^+$):** exact mass calculated for $[M+H]^+$ ($C_{11}H_{17}NF$) requires *m/z* 182.1340, found *m/z* 182.1340.

2-(3-Fluorophenyl)-*N,N*-dimethylpropan-1-amine (3g)

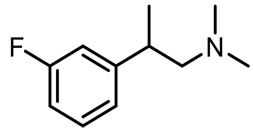

3g was prepared according to general procedure **A** and **B** using bromide **7g**, alcohol **8g**. Purification by column chromatography on silica gel (20% DMA-MIX to 70% DMA-MIX in DCM, Rf = 0.2 in 40% DMA-MIX) afforded compound **3g** in 84% (26 mg, procedure **A**) and 70% (25.4 mg, procedure **B**) yields as a yellow liquid.

^{1}H NMR (600 MHz, CDCl$_3$) δ 7.27 – 7.23 (m, 1H), 6.99 (d, *J* = 7.7 Hz, 1H), 6.92 – 6.86 (m, 2H), 2.94 – 2.88 (m, 1H), 2.43 (dd, *J* = 12.2, 7.2 Hz, 1H), 2.37 (dd, *J* = 12.2, 8.0 Hz, 1H), 2.22 (s, 6H), 1.24 (d, *J* = 6.9 Hz, 3H); **^{13}C NMR (150 MHz, CDCl$_3$) δ** 163.1 (d, *J* = 245.0 Hz), 149.0 (d, *J* = 6.8 Hz), 129.9 (d, *J* = 8.3 Hz), 123.0 (d, *J* = 2.6 Hz), 114.0 (d, *J* = 20.9 Hz), 113.1 (d, *J* = 21.1 Hz), 67.38 (2C), 45.98, 38.1, 20.3; **^{19}F NMR (659 MHz, CDCl$_3$) δ** -112.8 (s, 1F); **IR (neat) ν_{max}:** 2967, 2939, 2817, 2766, 1615, 1590, 1484, 1447, 1264, 1098, 1034, 871, 781, 695; **HRMS (ESI$^+$):** exact mass calculated for $[M+H]^+$ ($C_{11}H_{17}NF$) requires *m/z* 182.1340, found *m/z* 182.1341.

***N,N*-Dimethyl-2-(3-(trifluoromethyl)phenyl)propan-1-amine (3h)**

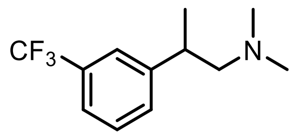

3h was prepared according to general procedure **A** and **B** using bromide **7h**, alcohol **8h**. Purification by column chromatography on silica gel (10% DMA-MIX to 50% DMA-MIX in DCM, Rf = 0.4 in 40% DMA-MIX) afforded compound **3h** in 63% (29 mg, procedure **A**) and 72% (31 mg, procedure **B**) yields as a yellow liquid.

^{1}H NMR (600 MHz, CDCl$_3$) δ 7.46 – 7.38 (m, 4H), 2.99 – 2.95 (m, 1H), 2.45 (dd, *J* = 12.2, 7.2 Hz, 1H), 2.39 (dd, *J* = 12.2, 8.0 Hz, 1H), 2.22 (s, 6H), 1.27 (d, *J* = 6.9 Hz, 3H); **^{13}C NMR (150 MHz, CDCl$_3$) δ** 147.1, 131.1 (q, *J* = 32.4 Hz), 130.7, 129.0, 124.2 (q, *J* = 271.8 Hz), 124.0 (q, *J* = 3.8 Hz), 123.2 (q, *J* = 3.8 Hz), 67.3, 46.0 (2C), 38.2, 20.3; **^{19}F NMR (659 MHz, CDCl$_3$) δ** -62.6 (s, 3F); **IR (neat) ν_{max}:** 2925, 1711, 1330, 1163, 1126, 1073, 749; **HRMS (ESI$^+$):** exact mass calculated for $[M+H]^+$ ($C_{12}H_{17}NF_3$) requires *m/z* 232.1308, found *m/z* 232.1315.

***N,N*-Dimethyl-2-(*m*-tolyl)propan-1-amine (3i)**

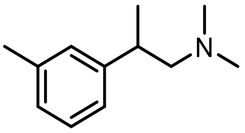

3i was prepared according to general procedure **A** and **B** using bromide **7i**, alcohol **8i**. Purification by column chromatography on silica (20% DMA-MIX to 70% DMA-MIX in DCM, Rf = 0.3 in 40% DMA-MIX) afforded compound **3i** in 65% (23 mg, procedure **A**) and 60% (19.7 mg, procedure **B**) yields as a yellow liquid.

^{1}H NMR (400 MHz, CDCl$_3$) δ 7.22 – 7.19 (m, 1H), 7.03 (d, *J* = 7.2 Hz, 3H), 3.01 (app. dd, *J* = 14.3, 7.1 Hz, 1H), 2.63 – 2.60 (m, 2H), 2.34 (s, 3H), 2.33 (s, 6H), 1.31 (d, *J* = 6.9 Hz, 3H); **^{13}C NMR (150 MHz, CDCl$_3$) δ** 145.1, 138.4, 128.7, 128.0, 127.5, 124.1, 66.9, 45.5 (2C), 37.6, 21.6, 20.7; **IR (neat) ν_{max}:** 3015, 2963, 2720, 1607, 1458, 1392, 1376, 1033, 781, 703; **HRMS (ESI$^+$):** exact mass calculated for $[M+H]^+$ ($C_{12}H_{20}N$) requires *m/z* 178.1590, found *m/z* 178.1591.

2-([1,1'-Biphenyl]-4-yl)-*N,N*-dimethylpropan-1-amine (3j)

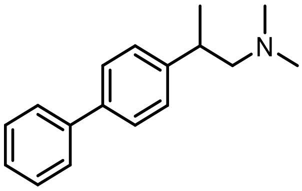

3j was prepared according to general procedure **A** and **B** using bromide **7j**, alcohol **8j**. Purification by column chromatography on silica gel (10% DMA-MIX to 50% DMA-MIX in DCM, Rf = 0.37 in 40% DMA-MIX) afforded compound **3j** in 71% (34 mg, procedure **A**) and 60% (27.2 mg, procedure **B**) yields as a white solid.

^{1}H NMR (400 MHz, CDCl$_3$) δ 7.60 – 7.53 (m, 4H), 7.43 (app. t, *J* = 7.6 Hz, 2H), 7.34 – 7.26 (m, 3H), 2.96 (app. dd, *J* = 14.7, 7.2 Hz, 1H), 2.51 – 2.42 (m, 2H), 2.26 (s, 6H), 1.30 (d, *J* = 6.9 Hz, 3H); **^{13}C NMR (150 MHz, CDCl$_3$) δ** 145.4, 141.3, 139.2, 128.8 (2C), 127.6 (2C), 127.3 (2C), 127.2 (2C), 127.1, 67.7, 46.1 (2C), 37.9, 20.4; **IR (neat) ν_{max}:** 2960, 2926, 2854, 2815, 2765, 1486, 1459, 1264, 1034, 1010, 764; **HRMS (ESI$^+$):** exact mass calculated for $[M+H]^+$ ($C_{17}H_{22}N$) requires *m/z* 240.1747, found *m/z* 240.1747.

_N,N_-Dimethyl-2-phenylhexan-1-amine (3k)

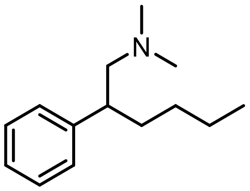

3k was prepared according to general procedure **A** and **B** using bromide **7k**, alcohol **8k**. Purification by column chromatography on silica gel (10% DMA-MIX to 70% DMA-MIX in DCM, Rf = 0.4 in 40% DMA-MIX) afforded compound **3k** in 53% (21.8 mg, procedure **A**) and 41% (17 mg, procedure **B**) yield as a yellow liquid.

^{1}H NMR (400 MHz, $CDCl_3$) δ 7.38 – 7.35 (m, 2H), 7.30 – 7.24 (m, 3H), 3.28 – 3.19 (m, 2H), 3.10 (app. dd, J = 11.7, 7.9 Hz, 1H), 2.54 (s, 6H), 1.86 – 1.78 (m, 1H), 1.64 – 1.55 (m, 1H), 1.32 – 1.05 (m, 4H), 0.82 (t, J = 7.2 Hz, 3H); **^{13}C NMR (150 MHz, $CDCl_3$) δ** 141.3, 129.3 (2C), 127.6 (3C), 64.2, 44.4 (2C), 42.1, 35.2, 29.0, 22.4, 13.9; **IR (neat) ν_{max}:** 2955, 2927, 2695, 1455, 1397, 1378, 763, 703; **HRMS (ESI$^+$):** exact mass calculated for $[M+H]^+$ ($C_{14}H_{24}N$) requires m/z 206.1903, found m/z 206.1910.

_N,N_-Dibenzyl-2-phenylpropan-1-amine (3l)

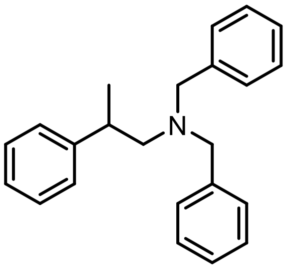

3l was prepared according to general procedure **A** using bromide **7a**. Purification by column chromatography on silica gel (20% DMA-MIX to 70% DMA-MIX in DCM, Rf = 0.4 in 40% DMA-MIX) afforded compound **3l** in 51% (32 mg) yield as a yellow semi-solid.

The spectroscopic data were in accordance with those reported.[33]

^{1}H NMR (400 MHz, $CDCl_3$) δ 7.31 – 7.22 (m, 13H), 7.11 – 7.08 (m, 2H), 3.59 (d, J = 13.7 Hz, 2H), 3.55 (d, J = 13.7 Hz, 2H), 3.09 – 2.96 (m, 1H), 2.65 – 2.49 (m, 2H), 1.24 (d, J = 6.9 Hz, 3H).

Ethyl _N_-methyl-_N_-(2-phenylpropyl)glycinate (3m)

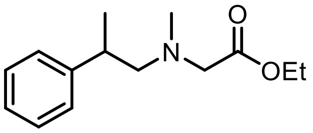

3m was prepared according to general procedure **C** using styrene **2a**. Purification by column chromatography on silica gel (10% DMA-MIX to 20% DMA-MIX in DCM, Rf = 0.57 in 10% DMA-MIX) afforded compound **3m** in 21% yield as a colourless liquid.

^{1}H NMR (400 MHz, CDCl$_3$) δ 7.30 (t, *J* = 7.7 Hz, 2H), 7.24 – 7.16 (m, 3H), 4.16 (q, *J* = 7.1 Hz, 2H), 3.29 (d, *J* = 16.6 Hz, 1H), 3.21 (d, *J* = 16.5 Hz, 1H), 2.98 – 2.86 (m, 1H), 2.72 (dd, *J* = 12.5, 6.7 Hz, 1H), 2.62 (dd, *J* = 12.4, 8.3 Hz, 1H), 2.39 (s, 3H), 1.26 (overlapped t, *J* = 7.4 Hz, 3H), 1.26 (overlapped t, *J* = 7.4 Hz, 3H). **^{13}C NMR (100 MHz, CDCl$_3$)** δ 171.3, 146.0, 128.5 (2C), 127.3 (2C), 126.3, 64.3, 60.4, 59.0, 42.7, 38.4, 20.1, 14.4. **IR (neat) ν_{max}:** 2937, 2802, 1738, 1494, 1454, 1185, 1052, 911, 736, 700; **HRMS (ESI$^+$):** exact mass calculated for $[M+H]^+$ ($C_{14}H_{22}NO_2$) requires *m/z* 236.1645, found *m/z* 236.1633.

N-Benzyl-*N*-methyl-2-phenylpropan-1-amine (3n)

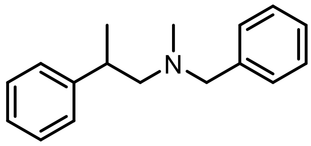

3n was prepared according to general procedure **C** using styrene **2a**. Purification by column chromatography on silica gel (10% DMA-MIX to 20% DMA-MIX in DCM, Rf = 0.48 in 10% DMA-MIX) afforded compound **3n** in 19% yield as a yellow liquid.

The spectroscopic data were in accordance with those reported.[34]

^{1}H NMR (400 MHz, CDCl$_3$) δ 7.39 – 7.27 (m, 4H), 7.24 – 7.14 (m, 6H), 3.52 (d, *J* = 13.2 Hz, 1H), 3.45 (d, *J* = 13.3 Hz, 1H), 3.04 – 2.91 (m, 1H), 2.52 (dd, *J* = 12.2, 6.8 Hz, 1H), 2.46 (dd, *J* = 12.2, 8.4 Hz, 1H), 2.21 (s, 3H), 1.27 (d, *J* = 6.9 Hz, 3H).

4-(1-(Dimethylamino)propan-2-yl)benzaldehyde (3o)

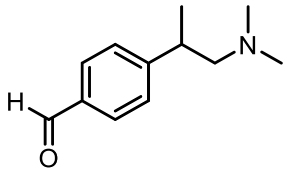

3o was prepared according to general procedure **C** using styrene **2o** at 1.2 M concentration. Purification by column chromatography on silica gel (0% DMA-MIX to 50% DMA-MIX in DCM, Rf = 0.20 in 50% DMA-MIX) afforded compound **3o** in 69% (26.3 mg) yield as a pale-yellow liquid.

^{1}H NMR (400 MHz, CDCl$_3$) δ 9.97 (s, 1H), 7.82 (d, *J* = 8.2 Hz, 2H), 7.37 (d, *J* = 8.2 Hz, 2H), 3.06 – 2.94 (m, 1H), 2.50 (dd, *J* = 12.2, 7.7 Hz, 1H), 2.40 (dd, *J* = 12.2, 7.7 Hz, 1H), 2.22 (s, 6H), 1.27 (d, *J* = 6.9 Hz, 3H); **^{13}C NMR (100 MHz, CDCl$_3$) δ** 192.1, 153.7, 136.0, 130.2 (2C), 128.0 (2C), 67.1, 46.0 (2C), 38.6, 20.1; **IR (neat) ν_{max}:** 2963, 2856, 2817, 1699, 1605, 1458, 1306, 1212, 1169, 1036, 841, 825; **HRMS (ESI$^+$):** exact mass calculated for $[M+H]^+$ ($C_{12}H_{18}NO^+$) requires *m/z* 192.1383, found *m/z* 192.1384.

Methyl 4-(1-(dimethylamino)propan-2-yl)benzoate (3p)

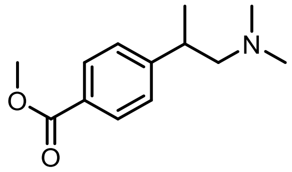

3p was prepared according to general procedure **C** using styrene **2p** at 1.2 M concentration. Purification by column chromatography on silica gel (0% DMA-MIX to 50% DMA-MIX in DCM, Rf = 0.20 in 50% DMA-MIX) afforded compound **3p** in 72% (32.0 mg) yield as a pale-yellow liquid.

^{1}H NMR (400 MHz, $CDCl_3$) δ 7.97 (d, *J* = 8.3 Hz, 2H), 7.27 (d, *J* = 8.3 Hz, 2H), 3.89 (s, 3H), 3.03 – 2.89 (m, 1H), 2.50 – 2.33 (m, 2H), 2.21 (s, 6H), 1.26 (d, *J* = 6.9 Hz, 3H); **^{13}C NMR (100 MHz, $CDCl_3$) δ** 167.3, 151.8, 130.0 (2C), 128.2, 127.3 (2C), 67.3, 52.1, 46.0 (2C), 38.4, 20.2; **IR (neat) ν_{max}:** 2950, 2768, 1721, 1610, 1435, 1277, 1181, 1111, 1016, 773, 707; **HRMS (ESI$^+$):** exact mass calculated for $[M+H]^+$ ($C_{13}H_{20}NO_2^+$) requires *m/z* 222.1489, found *m/z* 222.1490.

(4-(1-(Dimethylamino)propan-2-yl)phenyl)(pyrrolidin-1-yl)methanone (3q)

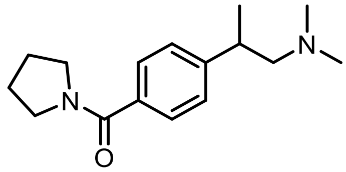

3q was prepared according to general procedure **C** using styrene **2q** at 1.2 M concentration. Purification by column chromatography on silica gel (0% DMA-MIX to 50% DMA-MIX in DCM, Rf = 0.20 in 50% DMA-MIX) afforded compound **3q** in 73% (37.9 mg) yield as a pale-yellow liquid.

^{1}H NMR (400 MHz, $CDCl_3$) δ 7.45 (d, *J* = 8.1 Hz, 2H), 7.21 (d, *J* = 8.1 Hz, 2H), 3.63 (t, *J* = 6.8 Hz, 2H), 3.45 (t, *J* = 6.8 Hz, 2H), 2.97 – 2.85 (m, 1H), 2.44 – 2.32 (m, 2H), 2.21 (s, 6H), 2.01 – 1.78 (m, 4H), 1.24 (d, *J* = 6.9 Hz, 3H); **^{13}C NMR (100 MHz, $CDCl_3$) δ** 169.9, 148.3, 135.2, 127.5 (2C), 127.1 (2C), 67.5, 49.8, 46.3, 46.0 (2C), 38.1, 26.6, 24.6, 20.2; **IR (neat) ν_{max}:** 2966, 2872, 2765, 1619, 1420, 1033, 841, 766; **HRMS (ESI$^+$):** exact mass calculated for $[M+H]^+$ ($C_{16}H_{25}N_2O^+$) requires *m/z* 261.1961, found *m/z* 261.1961.

***N*,*N*-Dimethyl-2-(4-(phenylsulfonyl)phenyl)propan-1-amine (3r)**

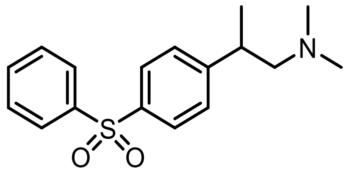

3r was prepared according to general procedure **C** using styrene **2r** at 1.2 M concentration. Purification by column chromatography on silica gel (0% DMA-MIX to 50% DMA-MIX in DCM, Rf = 0.20 in 50% DMA) afforded compound **3r** in 53% (32.0 mg) yield as a pale-yellow liquid.

^{1}H NMR (400 MHz, $CDCl_3$) δ 7.95 (d, *J* = 7.2 Hz, 2H), 7.86 (d, *J* = 8.4 Hz, 2H), 7.58 – 7.46 (m, 3H), 7.33 (d, *J* = 8.4 Hz, 2H), 3.01 – 2.90 (m, 1H), 2.46 (dd, *J* = 12.3, 7.9 Hz, 1H), 2.33 (dd, *J* = 12.3, 7.9 Hz, 1H), 2.19 (s, 6H), 1.21 (d, *J* = 6.9 Hz, 3H); **^{13}C NMR (100 MHz, $CDCl_3$) δ** 152.3, 142.0, 139.4, 133.2, 129.4 (2C), 128.2 (2C), 128.1 (2C), 127.8 (2C), 66.9, 46.0 (2C), 38.3, 20.4; **IR (neat) ν_{max}:** 2938, 2767, 1596, 1446,

1307, 1155, 1107, 717, 688, 589; **HRMS (ESI⁺):** exact mass calculated for $[M+H]^+$ ($C_{17}H_{22}NO_2S^+$) requires *m/z* 304.1366, found *m/z* 304.1369.

_N,N_-Dimethyl-2-(3-(methylsulfonyl)phenyl)propan-1-amine (3s)

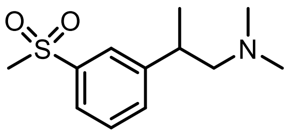

3s was prepared according to general procedure **C** using styrene **2s** at 1.2 M concentration. Purification by column chromatography on silica (0% DMA-MIX to 50% DMA-MIX in DCM, Rf = 0.20 in 50% DMA) afforded compound **3s** in 65% (31.2 mg) yield as a pale-yellow liquid.

^{1}H NMR (400 MHz, $CDCl_3$) δ 7.81 – 7.73 (m, 2H), 7.54 – 7.47 (m, 2H), 3.06 – 2.96 (m, 1H), 3.06 (s, 3H), 2.53 – 2.35 (m, 2H), 2.23 (s, 6H), 1.28 (d, *J* = 6.9 Hz, 3H); **^{13}C NMR (100 MHz, $CDCl_3$) δ** 148.1, 140.7, 132.7, 129.6, 126.1, 125.3, 67.0, 45.9 (2C), 44.7, 38.2, 20.3; **IR (neat) ν_{max}:** 2860, 2767, 1460, 1297, 1141, 1088, 1033, 958, 756, 696; **HRMS (ESI⁺):** exact mass calculated for $[M+H]^+$ ($C_{12}H_{20}NO_2S^+$) requires *m/z* 242.1209, found *m/z* 242.1211.

Ethyl 4-(dimethylamino)-3-phenylbutanoate (3t)

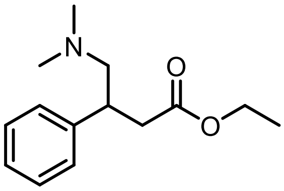

3t was prepared according to general procedures **A** and **D** using iodide **5t** with 8 equiv. of **1** at 1.2 M concentration on 0.15 mmol scale and cinnamate **2t**, respectively. Purification by column chromatography on silica gel (0% DMA-MIX to 80% DMA-MIX in DCM) afforded compound **3t** in 64% (22.5 mg, procedure **A**) and 55% (NMR, procedure **D**) yields as a yellow liquid.

^{1}H NMR (400 MHz, $CDCl_3$) δ 7.33 – 7.27 (m, 2H), 7.23 – 7.17 (m, 3H), 4.09 – 3.93 (m, 2H), 3.39 – 3.27 (m, 1H), 2.82 (dd, *J* = 15.4, 6.2 Hz, 1H), 2.56 – 2.34 (m, 3H), 2.23 (s, 6H), 1.13 (t, *J* = 7.1 Hz, 3H); **^{13}C NMR (150 MHz, $CDCl_3$) δ** 172.8, 143.0, 128.6 (2C), 127.6 (2C), 126.8, 65.7, 60.3, 45.9 (2C), 40.6, 39.6, 14.3; **IR (neat) ν_{max}:** 2941, 2767, 1729, 1455, 1371, 1263, 1181, 1138, 1026, 855, 759, 699; **HRMS (ESI⁺):** exact mass calculated for $[M+H]^+$ ($C_{14}H_{22}NO_2^+$) requires *m/z* 236.1645, found *m/z* 236.1643.

Ethyl 3-(4-chlorophenyl)-4-(dimethylamino)butanoate (3u)

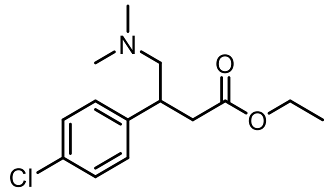

3u was prepared according to general procedure **D** using cinnamate **2u**. Purification by column chromatography on silica gel (0% DMA-MIX to 80% DMA-MIX in DCM) afforded compound **3u** in 42% (22.6 mg, 79% brsm) yield as a yellow liquid.

^{1}H NMR (400 MHz, $CDCl_3$) δ 7.28 – 7.24 (m, 2H), 7.16 – 7.12 (m, 2H), 4.07 – 3.97 (m, 2H), 3.36 – 3.24 (m, 1H), 2.80 (dd, *J* = 15.5, 5.9 Hz, 1H), 2.45 (dd, *J* = 15.5, 8.9 Hz, 1H), 2.42 (dd, *J* = 12.3, 8.6 Hz, 1H), 2.37 (dd, *J* = 12.3, 7.0 Hz, 1H), 2.21 (s, 6H), 1.14 (t, *J* = 7.1 Hz, 3H); **^{13}C NMR (150 MHz, $CDCl_3$) δ** 172.5, 141.5, 132.5, 129.0 (2C), 128.8 (2C), 65.5, 60.4, 45.9 (2C), 40.1, 39.5, 14.3; **IR (neat) ν_{max}:** 2977, 2942, 2906, 2860, 2819, 2769, 2727, 1729, 1493, 1460, 1371, 1264, 1180, 1159, 1139, 1027, 1014, 963, 858, 820; **HRMS (ESI$^+$):** exact mass calculated for $[M+H]^+$ ($C_{14}H_{21}NO_2Cl^+$) requires *m/z* 270.1267, found *m/z* 270.1261.

Ethyl 3-(4-bromophenyl)-4-(dimethylamino)butanoate (3v)

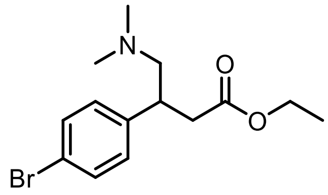

3v was prepared according to general procedure **D** using cinnamate **2v**. Purification by column chromatography on silica gel (0% DMA-MIX to 80% DMA-MIX in DCM) afforded compound **3v** in 30% (19 mg, 72% brsm) yield as a yellow liquid.

^{1}H NMR (400 MHz, $CDCl_3$) δ 7.41 (d, *J* = 8.4 Hz, 2H), 7.08 (d, *J* = 8.4 Hz, 2H), 4.07 – 3.97 (m, 2H), 3.33 – 3.24 (m, 1H), 2.79 (dd, *J* = 15.5, 5.9 Hz, 1H), 2.45 (dd, *J* = 15.5, 8.9 Hz, 1H), 2.43 – 2.34 (m, 2H), 2.21 (s, 6H), 1.14 (t, *J* = 7.1 Hz, 3H); **^{13}C NMR (150 MHz, $CDCl_3$) δ** 172.4, 142.0, 131.7 (2C), 129.4 (2C), 120.6, 65.4, 60.4, 45.9 (C2), 40.1, 39.4, 14.3; **IR (neat) ν_{max}:** 2976, 2941, 2859, 2820, 2769, 2726, 1730, 1489, 1460, 1390, 1371, 1307, 1264, 1212, 1181, 1159, 1139, 1097, 1073, 1027, 1010, 964, 858, 818; **HRMS (ESI$^+$):** exact mass calculated for $[M+H]^+$ ($C_{14}H_{21}NO_2Br^+$) requires *m/z* 314.0756, found *m/z* 314.0749.

Ethyl 4-(dimethylamino)-3-(*p*-tolyl)butanoate (3w)

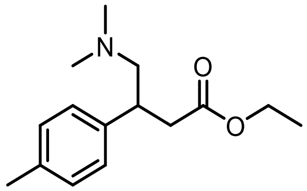

3w was prepared according to general procedure **D** using cinnamate **2w**. Purification by column chromatography on silica gel (0% DMA-MIX to 80% DMA-MIX in DCM) afforded compound **3w** in 43% (21.3 mg, 69% brsm) yield as a yellow liquid.

^{1}H NMR (400 MHz, CDCl$_3$) δ 7.10 (app. s, 4H), 4.07 – 3.97 (m, 2H), 3.29 (tt, *J* = 8.8, 6.4 Hz, 1H), 2.79 (dd, *J* = 15.3, 6.1 Hz, 1H), 2.48 (dd, *J* = 15.3, 8.7 Hz, 1H), 2.45 (dd, *J* = 12.3, 9.1 Hz, 1H), 2.38 (dd, *J* = 12.3, 6.5 Hz, 1H), 2.30 (s, 3H), 2.22 (s, 6H), 1.14 (t, *J* = 7.1 Hz, 3H); **^{13}C NMR (150 MHz, CDCl$_3$) δ** 172.8, 139.9, 136.3, 129.3 (2C), 127.5 (2C), 65.8, 60.3, 45.9 (2C), 40.2, 39.7, 21.2, 14.3; **IR (neat) ν_{max}:** 2977, 2942, 2860, 2819, 2768, 2729, 1733, 1515, 1459, 1371, 1350, 1329, 1263, 1215, 1181, 1159, 1139, 1097, 1059, 1029, 912, 812; **HRMS (ESI$^+$):** exact mass calculated for $[M+H]^+$ ($C_{15}H_{24}NO_2^+$) requires *m/z* 250.1807, found *m/z* 250.1801.

Reduction of styrenes by tetrabutylammonium iodide

Procedure E: Formal hydrogenation of styrenes

The corresponding styrene (0.30 mmol, 1.00 equiv.) and TBAI (443 mg, 1.20 mmol, 4.00 equiv.) were added to an oven-dried vial containing a magnetic stirrer bar. Then, 0.5 mL TFA (0.6 M) was added, and the solution was stirred in a preheated oil bath at 75 °C for 4 days. Upon completion, the reaction was allowed to cool to room temperature and was diluted with Et_2O and washed with sat. $NaHCO_3$ (aq.) solution. The organic phase was dried over $MgSO_4$, and the solvents were removed under reduced pressure to give the crude product.

1-Bromo-4-ethylbenzene (10a)

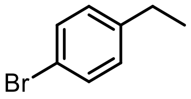

10a was prepared according to general procedure **E** using styrene **2e**. The product was purified via column chromatography on silica gel (pentane) to afford compound **10a** in 72% (40.0 mg) yield as a colourless oil.

The spectroscopic data were in accordance with those reported.[35]

^{1}H NMR (400 MHz, $CDCl_3$) δ 7.40 (d, *J* = 8.3 Hz, 2H), 7.07 (d, *J* = 8.3 Hz, 2H), 2.60 (q, *J* = 7.6 Hz, 2H), 1.22 (t, *J* = 7.6 Hz, 3H).

Methyl 4-ethylbenzoate (10b)

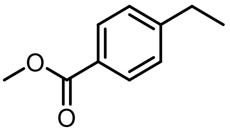

10b was prepared according to general procedure **E** using styrene **2n**. The product was purified via column chromatography on silica gel (0–10% EtOAc in heptane) to afford a compound **10b** in 54% (26.4 mg) yield as a colorless oil.

The spectroscopic data were in accordance with those reported.[36]

^{1}H NMR (400 MHz, $CDCl_3$) δ 7.96 (d, *J* = 8.2 Hz, 2H), 7.26 (d, *J* = 8.2 Hz, 2H), 3.90 (s, 3H), 2.70 (q, *J* = 7.6 Hz, 2H), 1.25 (t, *J* = 7.6 Hz, 3H).

1-Ethyl-4-(phenylsulfonyl)benzene (10c)

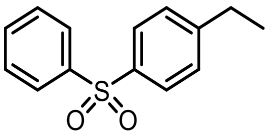

10c was prepared according to general procedure **E** using styrene **2p**. The product was purified via column chromatography on silica gel (0–20% EtOAc in heptane) to afford compound **10c** in 84% (62.3 mg) yield as a colourless oil.

The spectroscopic data were in accordance with those reported.[37]

^{1}H NMR (400 MHz, CDCl$_3$) δ 7.98 – 7.91 (m, 2H), 7.88 – 7.81 (m, 2H), 7.58 – 7.45 (m, 3H), 7.32 (d, *J* = 8.5 Hz, 2H), 2.69 (q, *J* = 7.6 Hz, 2H), 1.23 (t, *J* = 7.6 Hz, 3H).

1-Ethyl-3-(methylsulfonyl)benzene (10d)

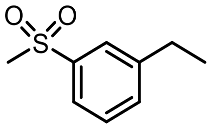

10d was prepared according to general procedure **F** using styrene **2q**. The product was purified via column chromatography on silica gel (0–30% EtOAc in heptane) to afford compound **10d** in 65% (35.7 mg) as a colourless oil.

^{1}H NMR (400 MHz, CDCl$_3$) δ 7.79–7.71 (m, 2H), 7.51–7.44 (m, 2H), 3.05 (s, 3H), 2.75 (q, *J* = 7.6 Hz, 2H), 1.28 (t, *J* = 7.6 Hz, 3H); **^{13}C NMR (150 MHz, CDCl$_3$)** δ 146.1, 140.7, 133.5, 129.5 , 126.7, 124.8, 44.7, 28.8, 15.4; **IR (neat) ν_{max}:** 2967, 2929, 1295, 1139, 956, 752, 691, 533; **HRMS (ESI$^+$):** exact mass calculated for $[M+Na]^+$ ($C_9H_{12}NaO_2S^+$) requires 207.0450, found 207.0442.

Iminium iodides derived from other amines

Iminium iodides other than those derived from dimethylamine, dibenzylamine, sarcosine ethyl ester and benzylmethylamine (see scope table) gave negative results. The reaction of styrene with iminium iodides derived from piperidine and pyrrolidine did not lead to formation of the product and only returned a complex mixture of products of non-specific decomposition.

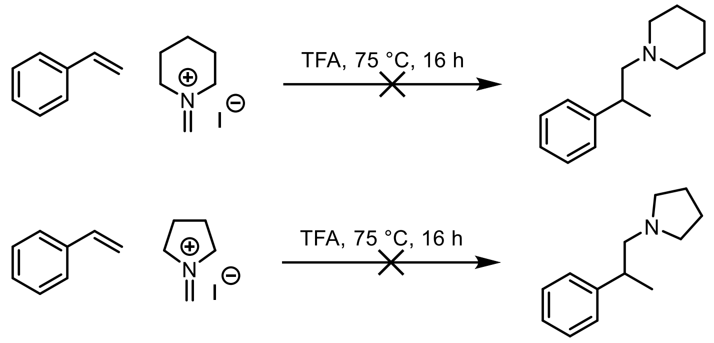

Investigation of other potential substrates

The use of β-methylstyrene, resulted in formation of only 16% of the desired product **SI-1**.

***N,N*-Dimethyl-2-phenylbutan-1-amine (SI-1)**

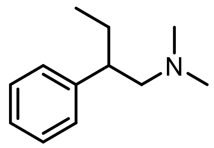

SI-1 was prepared according to general procedure **C** using styrene **2a**. Purification by column chromatography on silica gel (10% DMA-MIX to 50% DMA-MIX in DCM, Rf = 0.46 in 40% DMA-MIX) afforded compound **SI-1** in 16% yield as a yellow liquid.

^{1}H NMR (400 MHz, CDCl$_3$) δ 7.30 (t, *J* = 7.4 Hz, 2H), 7.22 – 7.15 (m, 3H), 2.69 – 2.60 (m, 1H), 2.50 (dd, *J* = 9.8, 4.8 Hz, 1H), 2.45 (dd, *J* = 9.8, 5.5 Hz, 1H), 2.21 (s, 6H), 1.82 (dqd, *J* = 14.9, 7.5, 4.5 Hz, 1H), 1.50 (ddq, *J* = 14.6, 9.7, 7.3 Hz, 1H), 0.77 (t, *J* = 7.4 Hz, 3H).**^{13}C NMR (100 MHz, CDCl$_3$)** δ 144.5, 128.5 (2C), 127.9 (2C), 126.3, 66.3, 46.1, 46.0 (2C), 27.6, 12.1. **IR (neat) ν_{max}:** 2964, 2931, 2765, 1494, 1454, 1037, 911, 736, 700; **HRMS (ESI$^+$):** exact mass calculated for $[M+H]^+$ ($C_{12}H_{20}N$) requires *m/z* 178.1590, found *m/z* 178.1586.

The following other compounds did not yield the desired aminomethylenated product, or gave yields lower than 30% under the general conditions (Scheme S1).

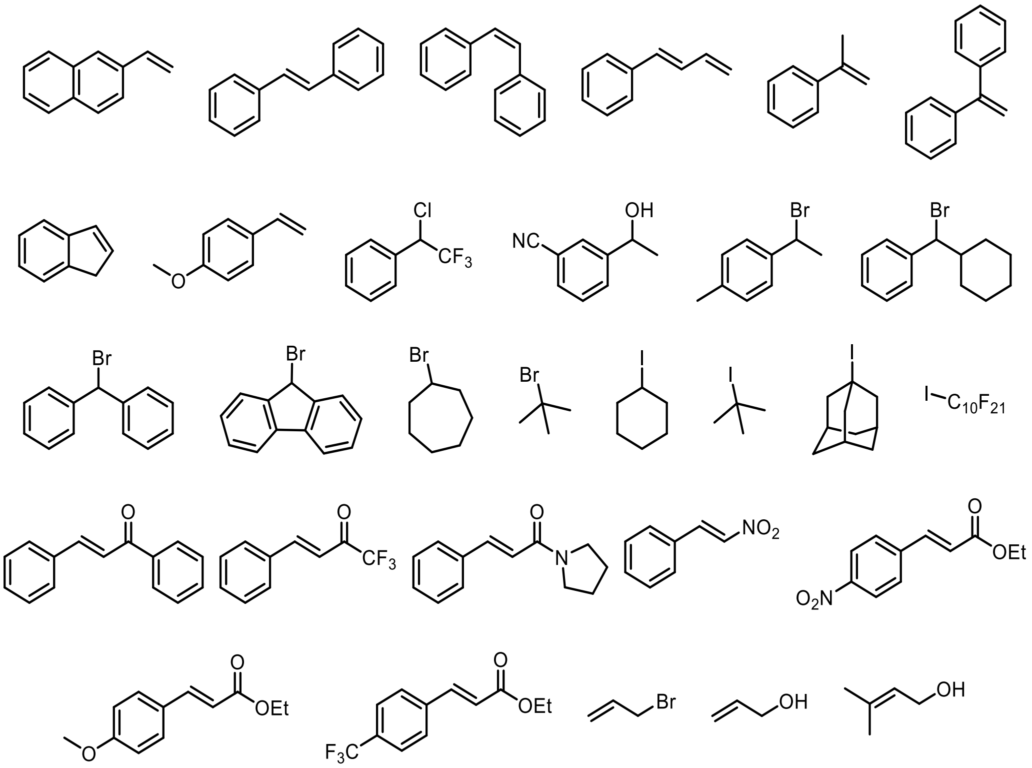

Scheme S1. Limitations of the presented method.

Mechanistic investigation

A) Counterion investigation

Experiments were performed according to general procedures **A** and **C** using iminium salts **1a–1c** (Scheme S2).

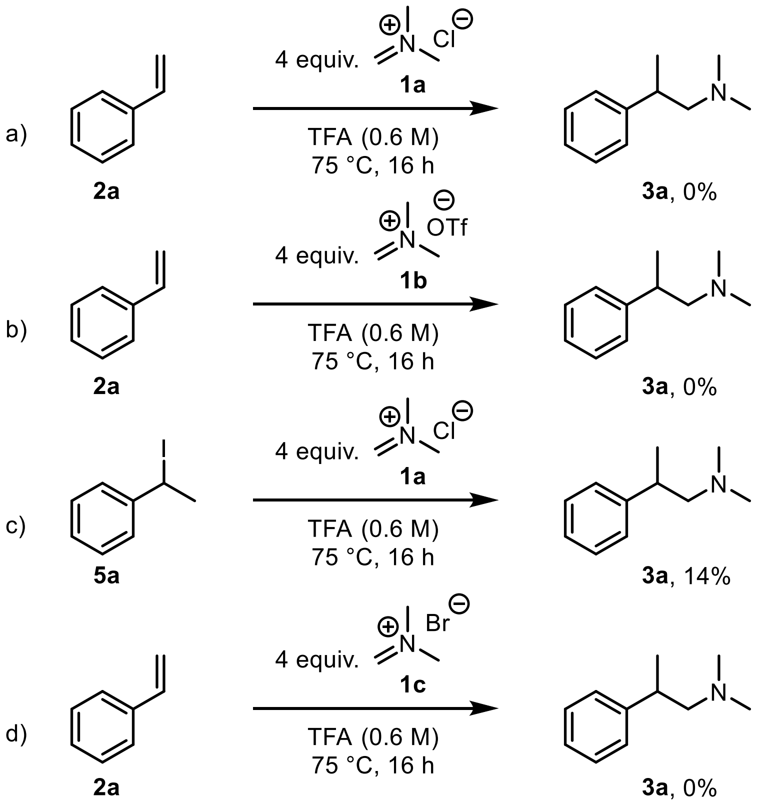

Scheme S2 Investigations on the effect of counterions on the reaction outcome.

While the reaction with iminium chloride, triflate or bromide did not yield product **3a** when using styrene, benzylic iodide **5a** successfully reacted with iminium chloride to form **3a**, albeit in low yield. This clearly indicates the need for an iodide counterion to act as a reductant in addition to generate the key benzylic iodide intermediate.

B) Experiment with deuterium labelled TFA

***N,N*-Dimethyl-2-phenylpropan-1-amine-3,3,3-d3 (3a-*d*3)**

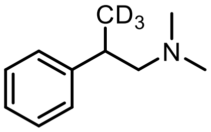

Was prepared according to general procedure **C** using styrene **2a** and deuterated trifluoroacetic acid. The product was purified via column chromatography on silica gel (10% DMA-MIX to 75% DMA-MIX in DCM, Rf = 0.25 in 40% DMA-MIX) to afford **3a-*d*3** as a colourless oil (16.8 mg, 51%).

^{1}H NMR (400 MHz, CDCl$_3$) δ 7.33 – 7.27 (m, 2H), 7.23 – 7.16 (m, 3H), 2.88 (t, *J* = 7.2 Hz, 1H), 2.40 (d, *J* = 7.3 Hz, 2H), 2.23 (s, 6H); **^{13}C NMR (100 MHz, CDCl$_3$)** δ 146.3, 128.5 (2C), 127.3 (2C), 126.3, 67.7, 46.0 (2C), 38.01, 29.9 (CD$_3$); **IR (neat) ν_{max}:** 3367, 3206, 956, 2920, 2851, 1659, 1634, 1464, 1377, 964, 888; **HRMS (ESI$^+$):** exact mass calculated for $[M+H]^+$ ($C_{11}H_{15}D_3N^+$) requires 167.1622, found 167.1620.

<u>C) Experiments with Ph-SS-Ph and TBAI</u>

The reaction in the presence of diphenyl disulfide and tetrabutylammonium iodide yielded sulfide **9a** indicating the formation of a benzylic radical intermediate (Scheme S3). Control reactions showed that 1. iodide is needed as a stoichiometric reductant, 2. not just as a phase transfer reagent and 3. does not reduce diphenyl disulfide during the reaction.

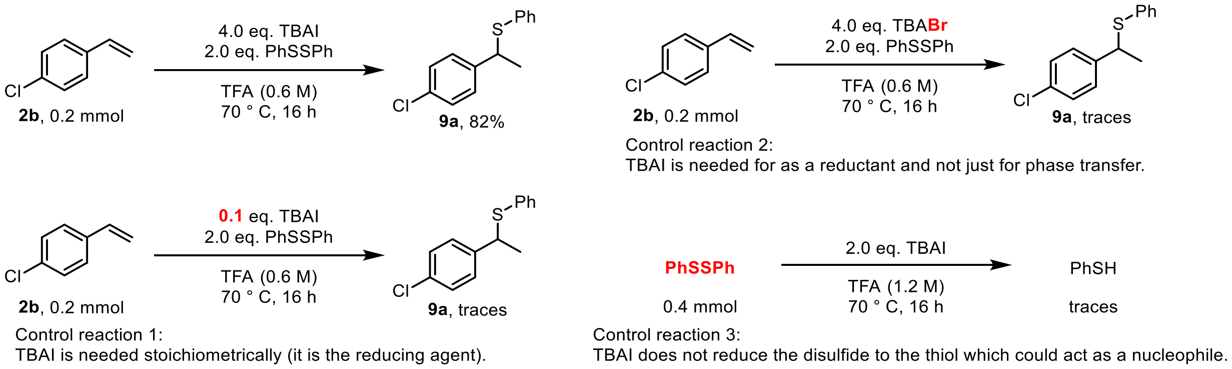

Scheme S3. The reaction of styrene 2b with TBAI and PhSSPh.

(1-(4-Chlorophenyl)ethyl)(phenyl)sulfane (9a)

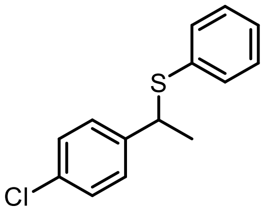

Diphenyl disulfide (87.3 mg, 0.40 mmol, 2.00 equiv.) and TBAI (295 mg, 0.80 mmol, 4.00 equiv.) were added to an oven-dried vial containing a magnetic stirrer bar. Then, 0.33 mL TFA (0.6 M) was added, followed by styrene **2b** (28.6 mg, 0.20 mmol, 1.00 equiv.), and the solution was stirred in a preheated oil bath at 70 °C for 16 h. Upon completion, the reaction was allowed to cool to room temperature and was diluted with Et$_2$O and washed with sat. NaHCO$_3$ (aq.) solution. The organic phase was dried over MgSO$_4$, and the solvents were removed under reduced pressure. Purification by column chromatography on silica gel (heptane) afforded compound **9a** in 82% (41.0 mg) yield as a white solid.

The spectroscopic data were in accordance with those reported.[38]

^{1}H NMR (400 MHz, CDCl$_3$): δ 7.30 – 7.18 (m, 8H), 4.29 (q, *J* = 7.0 Hz, 1H), 1.60 (d, *J* = 7.0 Hz, 3H).

D) TEMPO trapping experiments

Experiments were performed according to general procedure **A** using HFIP instead of TFA as the solvent. The TEMPO was added alongside either Eschenmoser´s salt **1** or TBAI as required (Scheme S4).

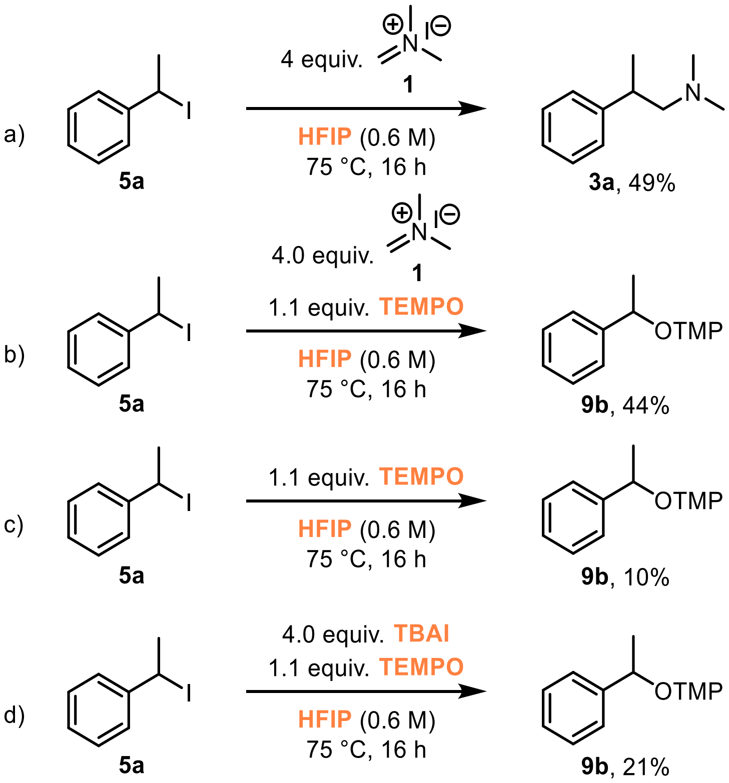

Scheme S4 TEMPO trapping experiments.

a) Due to the sensitivity of TEMPO to acidic conditions, the reaction was performed in HFIP, proceeding in 49% yield. b) Performing the reaction in presence of TEMPO provided similar yields of TEMPO-adduct **9b**, which unambiguously proves the formation of benzylic radical. c) Next, a reaction with TEMPO without Eschenmoser´s salt was performed providing 10% yield of TEMPO-adduct **9b**. d) The yield is, however, increased in presence of TBAI as an iodide source. This suggests that the iodide counter anion facilitates homolysis of the benzylic C–I bond.

E) Using TEMPO adduct **9c** as the staring material

When TEMPO adduct **9c** (synthesized according to Ref. 39) was subjected to the reaction conditions (Scheme S5), product **3b** formed in 30% yield, showcasing that thermally produced radicals undergo the aminomethylenation reaction.

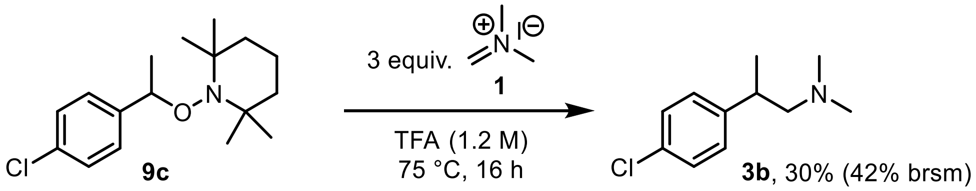

Scheme S5. TEMPO adduct 9c as a precursor of a benzylic radical.

F) Kinetics for cinnamates

A kinetic measurement was performed (Scheme S6), revealing the formation of the iodo-Michael adduct (in pink). Interestingly, consumption of the starting material (in blue) occurs only in the first hour. The rest of the time, it appears that the formed iodo-Michael adduct is transformed into the product (in orange).

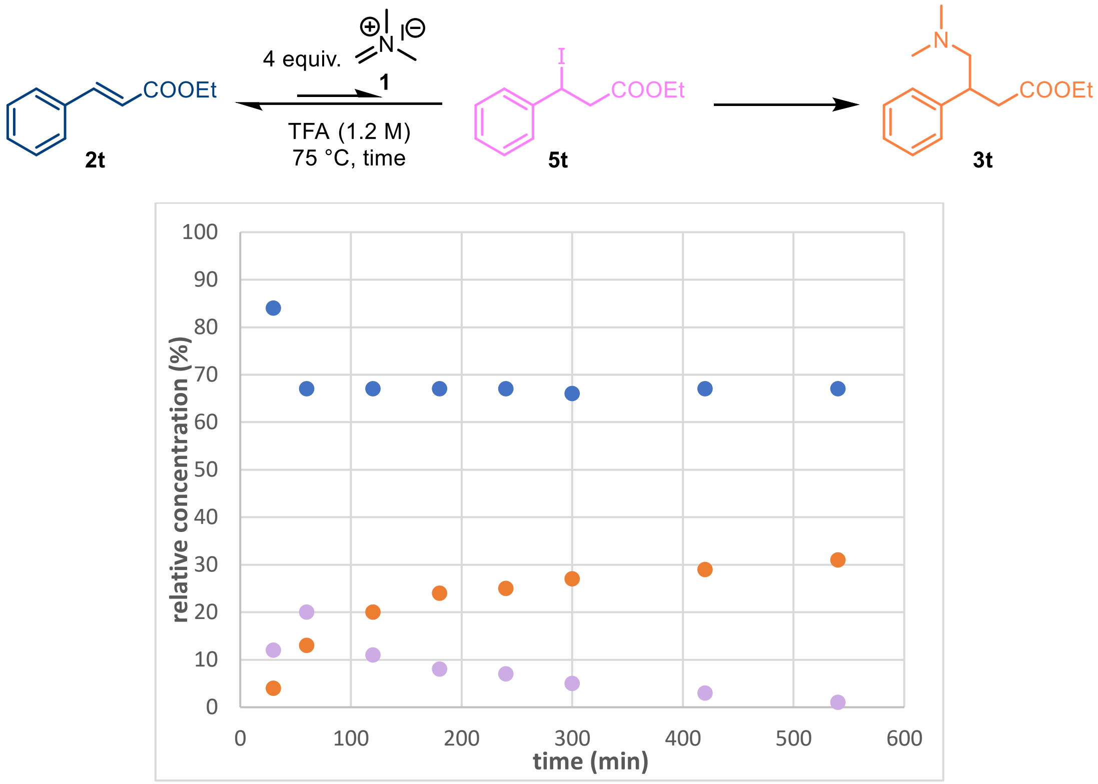

Scheme S6 The reaction profile of cinnamate 2t and Eschenmoser's salt.

To find out the relationship between the iodide, product and starting cinnamate (Scheme S7), the iodo-Michael adduct was subjected again to the same conditions, providing 64% of product and 30% of ethyl cinnamate. Similarly, exposure of the iodo-Michael adduct to 1.2 M TFA showed formation of 22% ethyl cinnamate after one hour, meaning there is an equilibrium between the iodo-Michael adduct and cinnamate with HI.

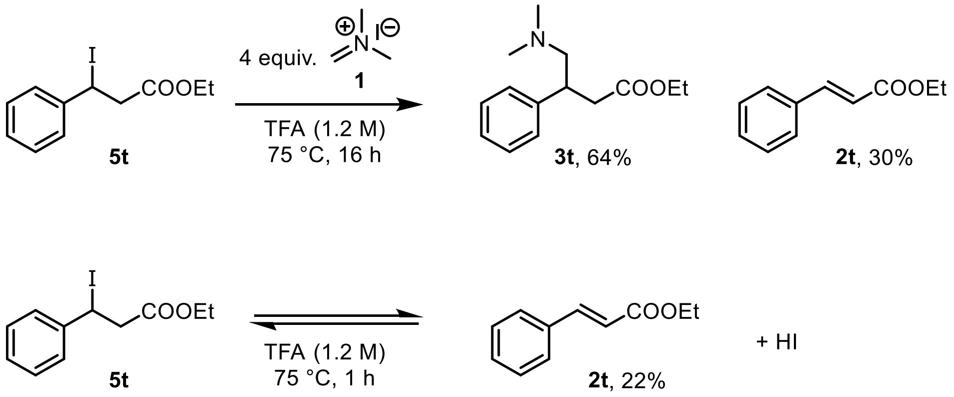

Scheme S7 The behavior of benzylic iodide 5t under the reaction conditions.

G) Aminals with iodide sources

To probe whether addition of iodide to the reaction described in reference 13 could trigger a reversal of product selectivity, a series of reactions was conducted.

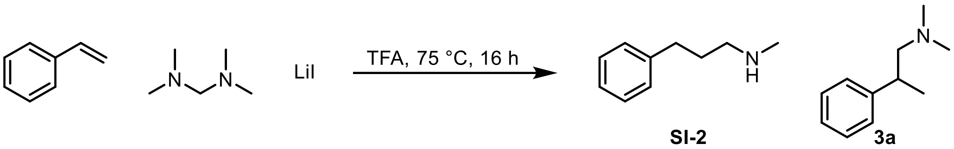

			**SI-2**	**3a**
4 eq.	4 eq.		85% NMR yield	6% NMR yield
	KI		sole product	not observed
	TBAI		sole product	not observed

Scheme S8 Addition of iodide sources to a protocol optimized for linear hydroaminomethylation.

Whereas potassium iodide and tetrabutylammonium iodide did not lead to the formation of any branched product (**3a**), the addition of lithium iodide to a reaction containing styrene, tetramethylmethylenediamine and TFA led to the formation of small amounts of **3a** (6% NMR yield), with the major species being the linear hydroaminomethylation product **SI-2** (Scheme S8).

Procedure:

A round-bottom flask charged with *N,N,N′,N′*-tetramethyldiaminomethane (4 equiv.) and a magnetic stir-bar under argon-atmosphere was cooled to 0 °C. After this, trifluoroacetic acid (TFA, 0.6 M with respect to the alkene) was added slowly, maintaining the low temperature of the contents of the flask. After completed addition of TFA, styrene **2a** (1.0 equiv., 0.5 mmol) and lithium iodide (4 equiv., 2.0 mmol) were added in one portion, the flask was sealed and placed in an oil bath at 75 °C. The reaction was vigorously stirred at this temperature for 16 h, after which it was allowed to cool to room temperature. Subsequently, volatile components were removed under reduced pressure. The crude mixture was then treated with aqueous sodium hydroxide (1 M – 2 mL/1 mmol substrate) and dichloromethane (1 mL/1 mmol substrate) and stirred vigorously at room temperature for 1 h. After this time, aqueous sodium hydroxide (5 M) was added until the reaction mixture reached pH 12. The resulting biphasic mixture was separated, and the aqueous phase was extracted with dichloromethane (3 x 200 mL). The combined organic phases were then dried over anhydrous sodium sulfate and filtered. The filtrate was concentrated under reduced pressure to afford the crude product mixture, containing the secondary amine **SI-2** (85 % NMR yield) and compound **3a** (6% NMR yield).

Formation of I_2 during the reaction

Iodine crystals forming in a successful reaction

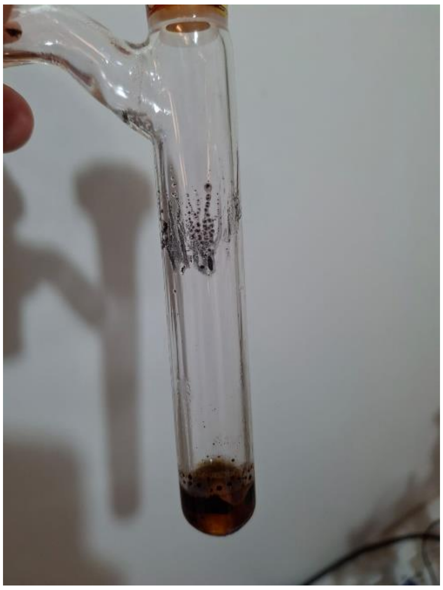

Spatula tip of obtained iodine crystals (left) and commercial iodine (right) in heptane

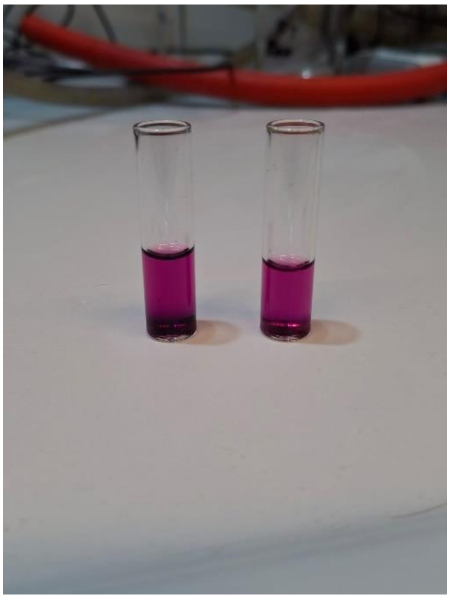

Spatula tip of obtained iodine crystals (left) and commercial iodine (right) and a spatula tip of cornstarch in H_2O

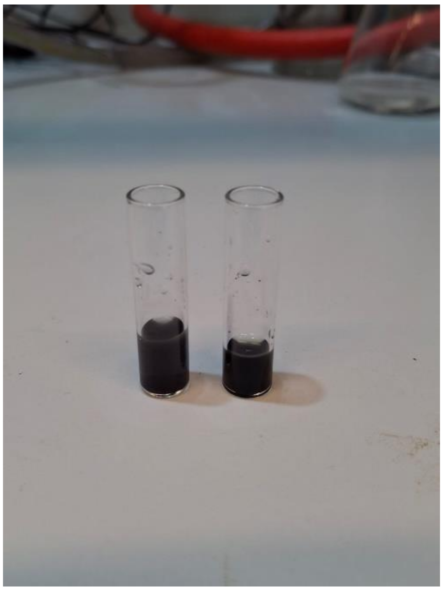

NMR Spectra

Styrenes

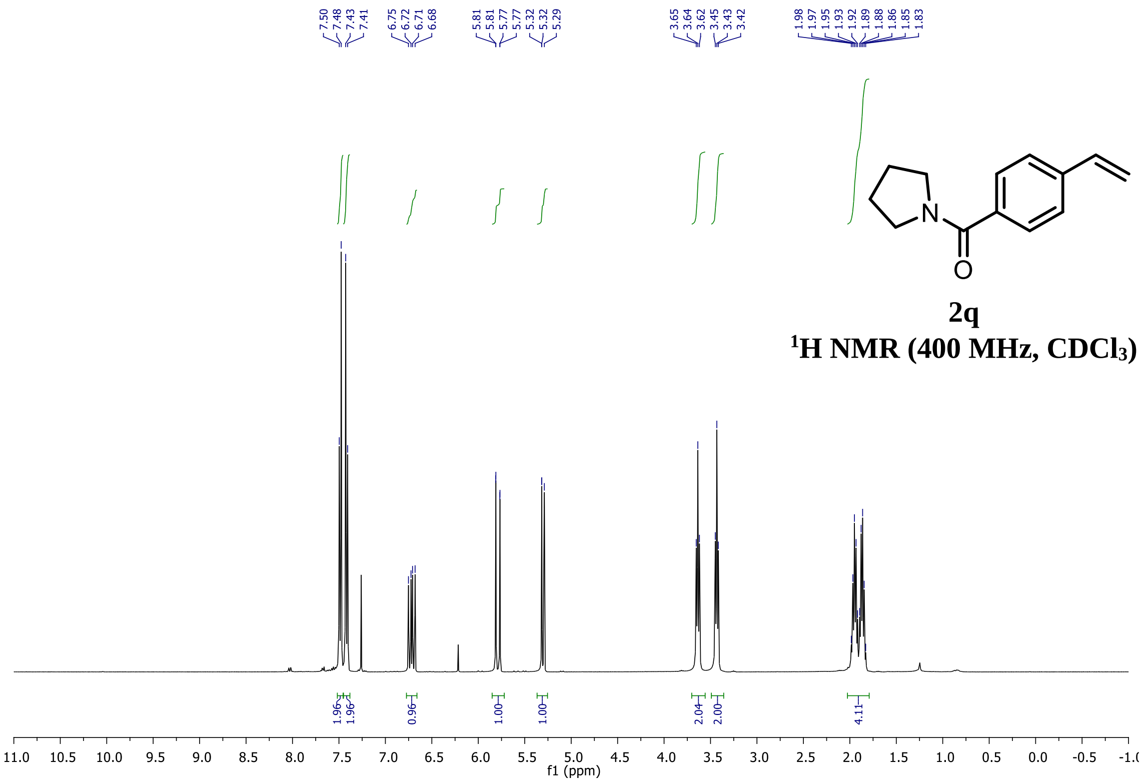

2q
^{1}H NMR (400 MHz, $CDCl_3$)

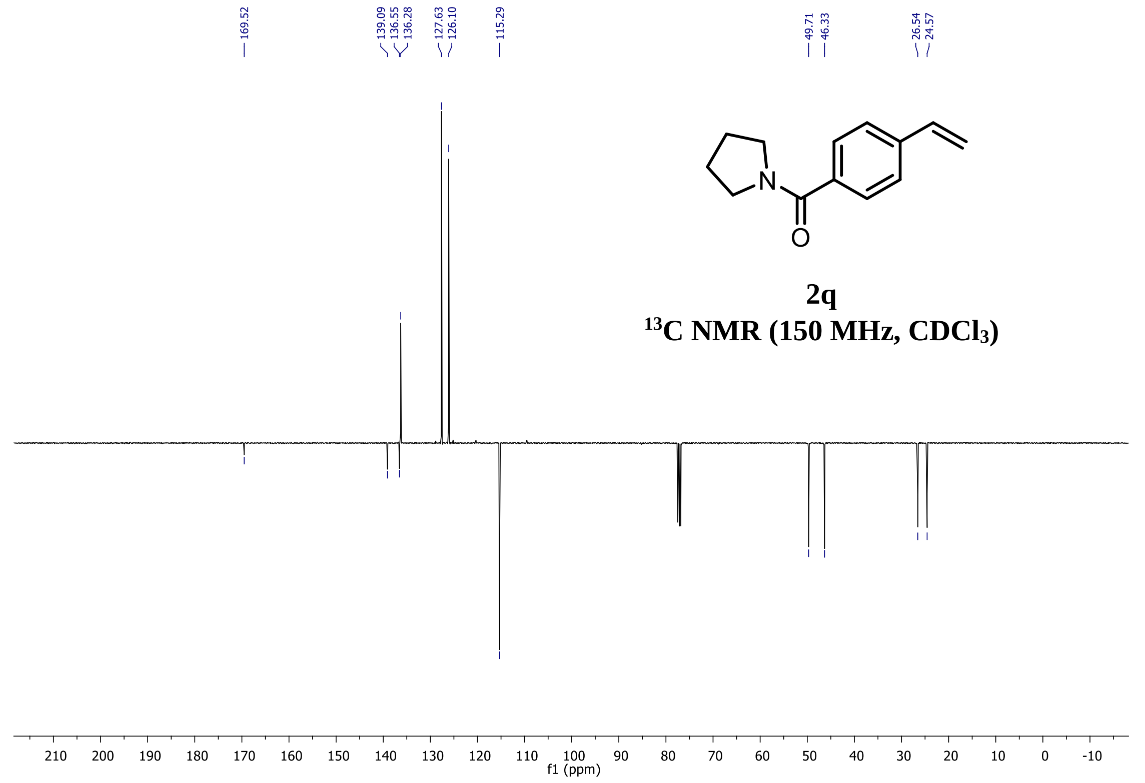

2q
^{13}C NMR (150 MHz, $CDCl_3$)

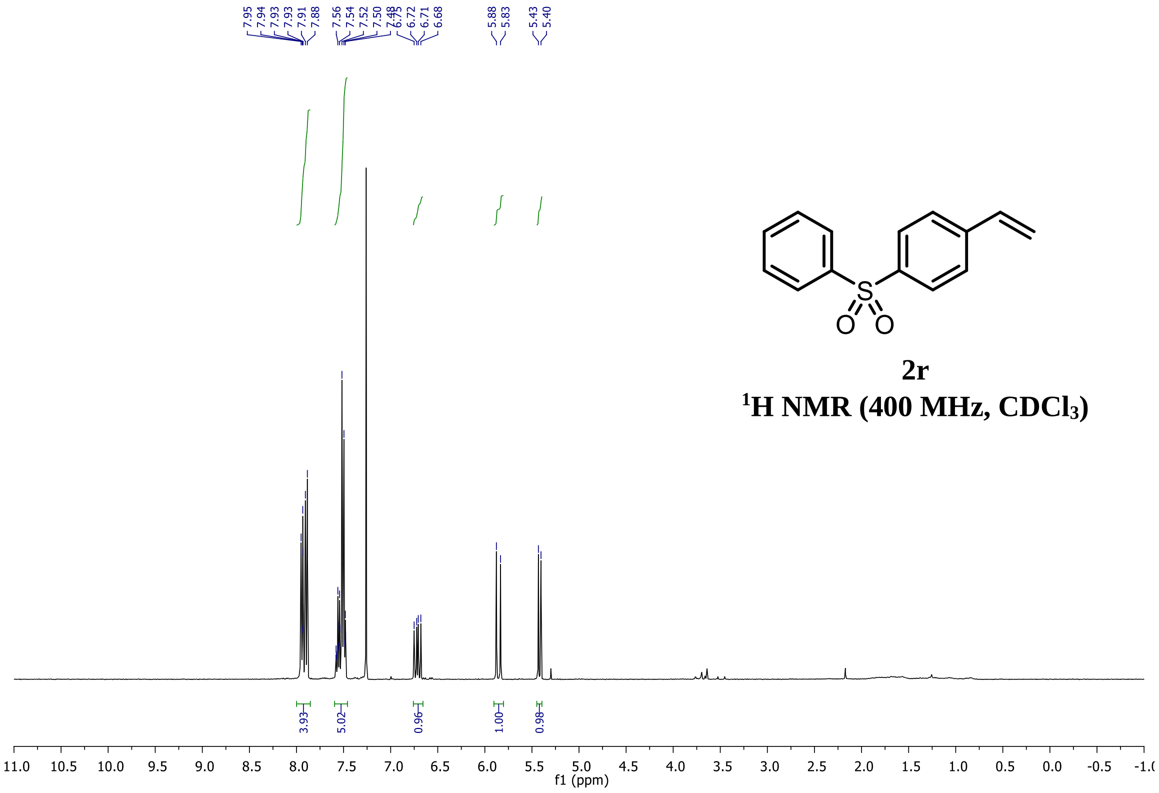
2r
^{1}H NMR (400 MHz, $CDCl_3$)
3.93
5.02
0.96
1.00
0.98
f1 (ppm)

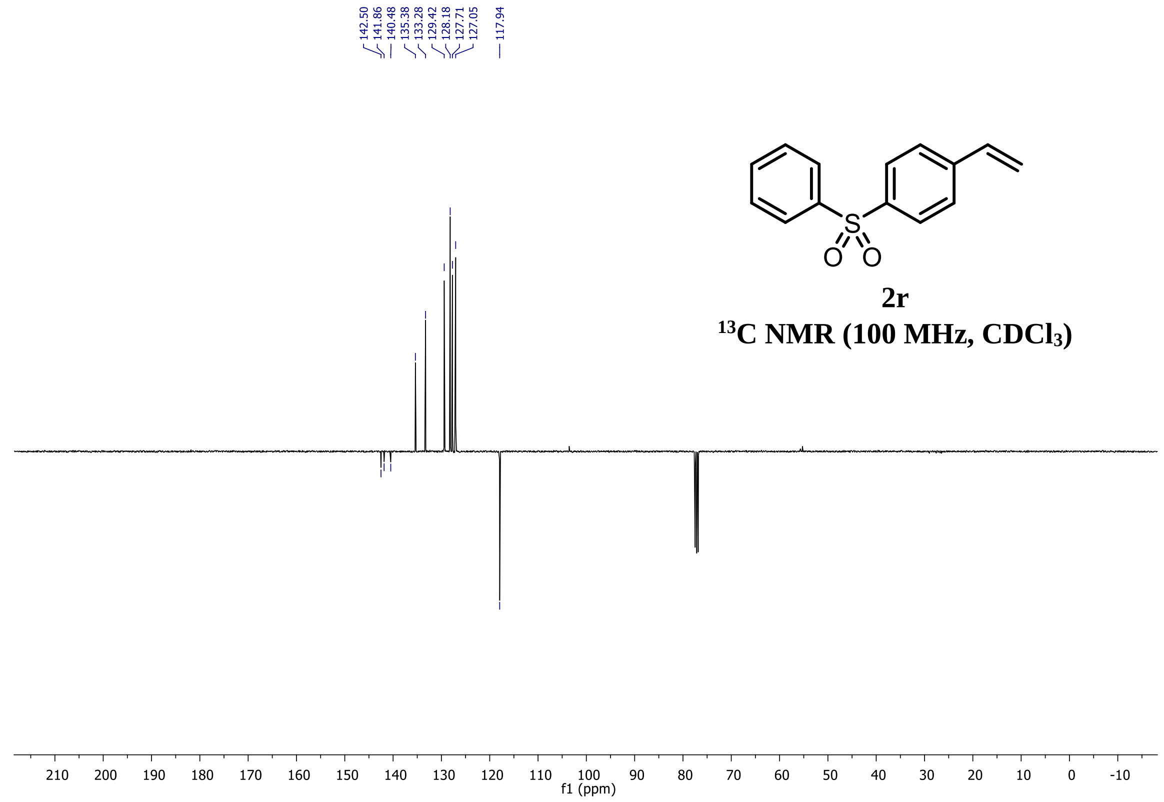
2r
^{13}C NMR (100 MHz, $CDCl_3$)
f1 (ppm)

Benzyl halides

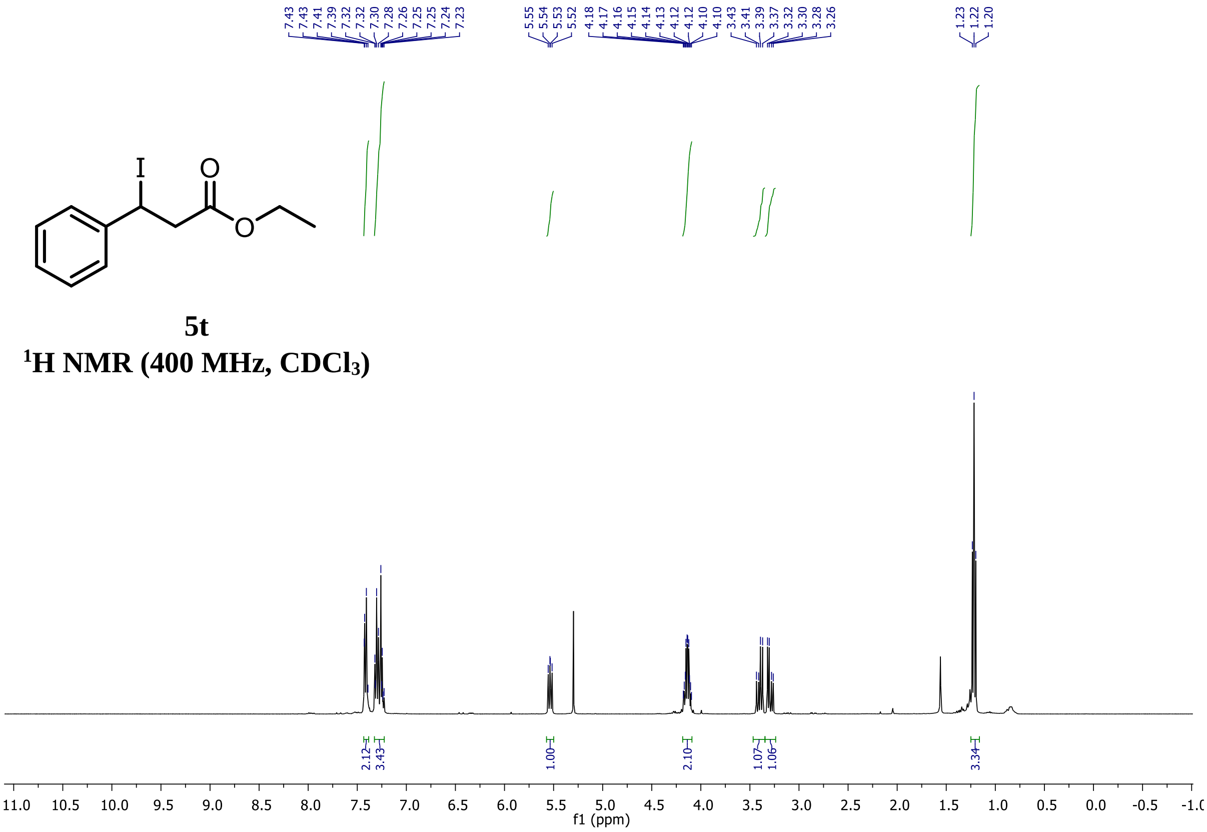

5t
^{1}H NMR (400 MHz, $CDCl_3$)

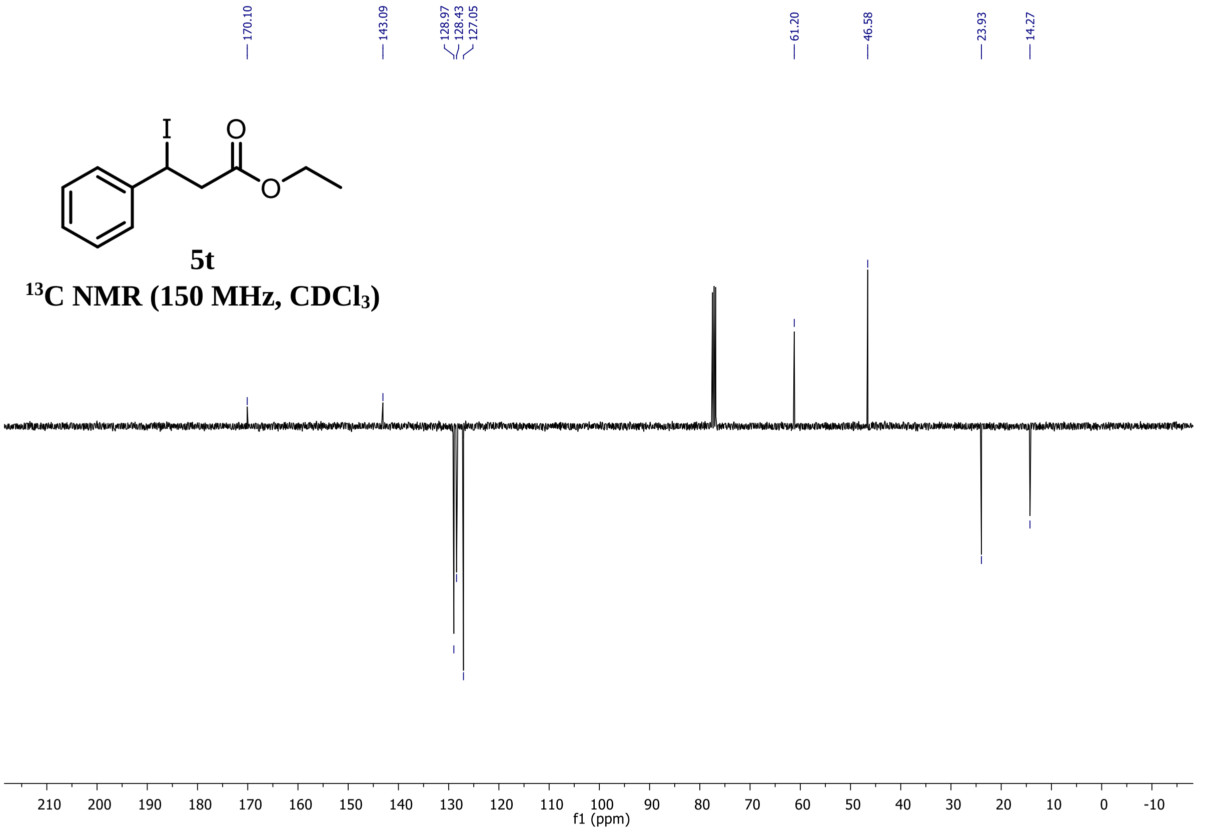

5t
^{13}C NMR (150 MHz, $CDCl_3$)

Hydroaminomethylation of styrenes and aminomethylation of benzylic halides or alcohols

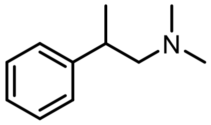

3a

^{1}H NMR (400 MHz, CDCl$_3$)

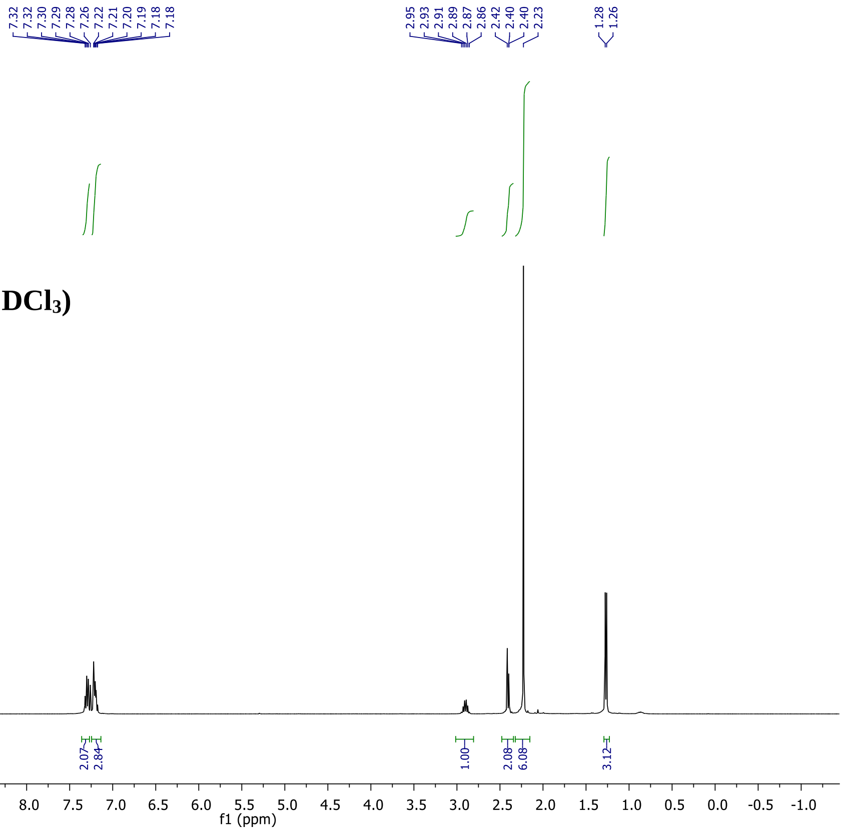

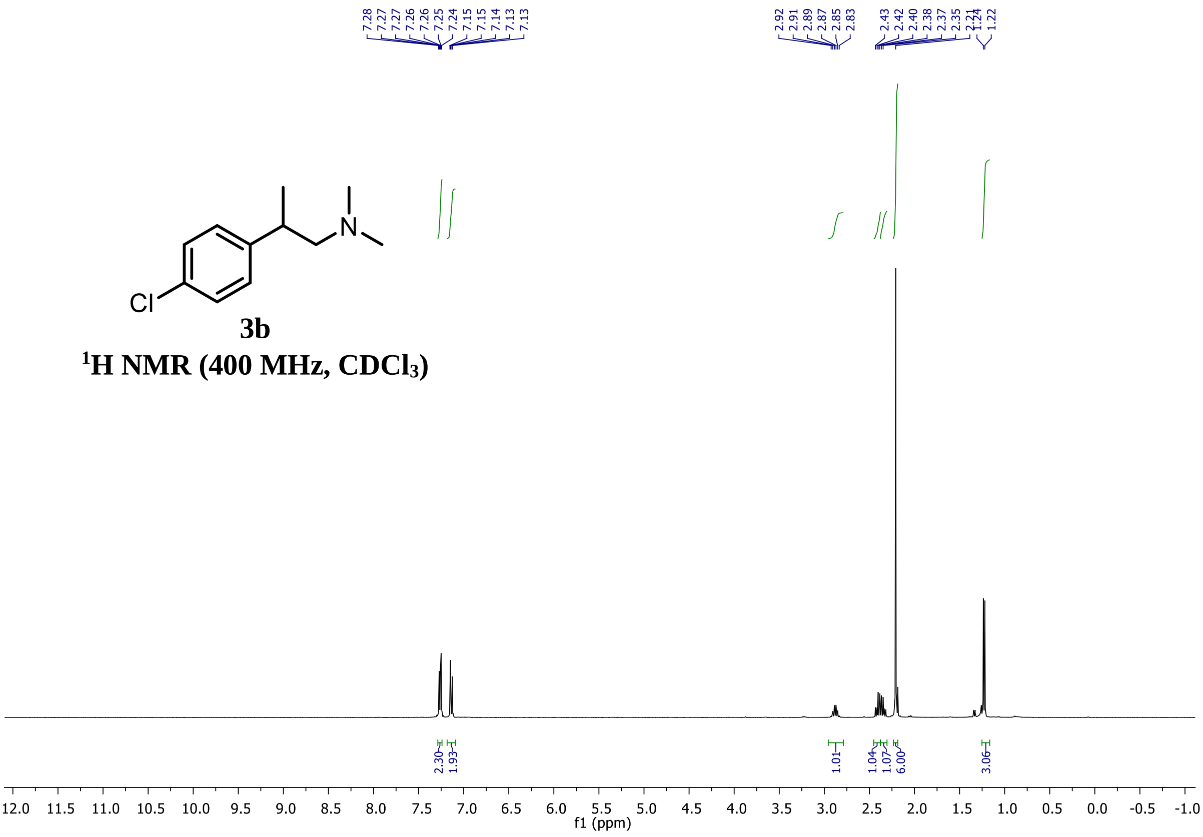
Cl
N
3b
¹H NMR (400 MHz, CDCl₃)
7.28 7.27 7.27 7.26 7.25 7.24 7.15 7.15 7.14 7.13 7.13
2.92 2.91 2.89 2.87 2.85 2.83
2.43 2.42 2.40 2.38 2.37 2.35 2.21 1.24 1.22
2.30 1.93 1.01 1.04 1.07 6.00 3.06
12.0 11.5 11.0 10.5 10.0 9.5 9.0 8.5 8.0 7.5 7.0 6.5 6.0 5.5 5.0 4.5 4.0 3.5 3.0 2.5 2.0 1.5 1.0 0.5 0.0 -0.5 -1.0
f1 (ppm)

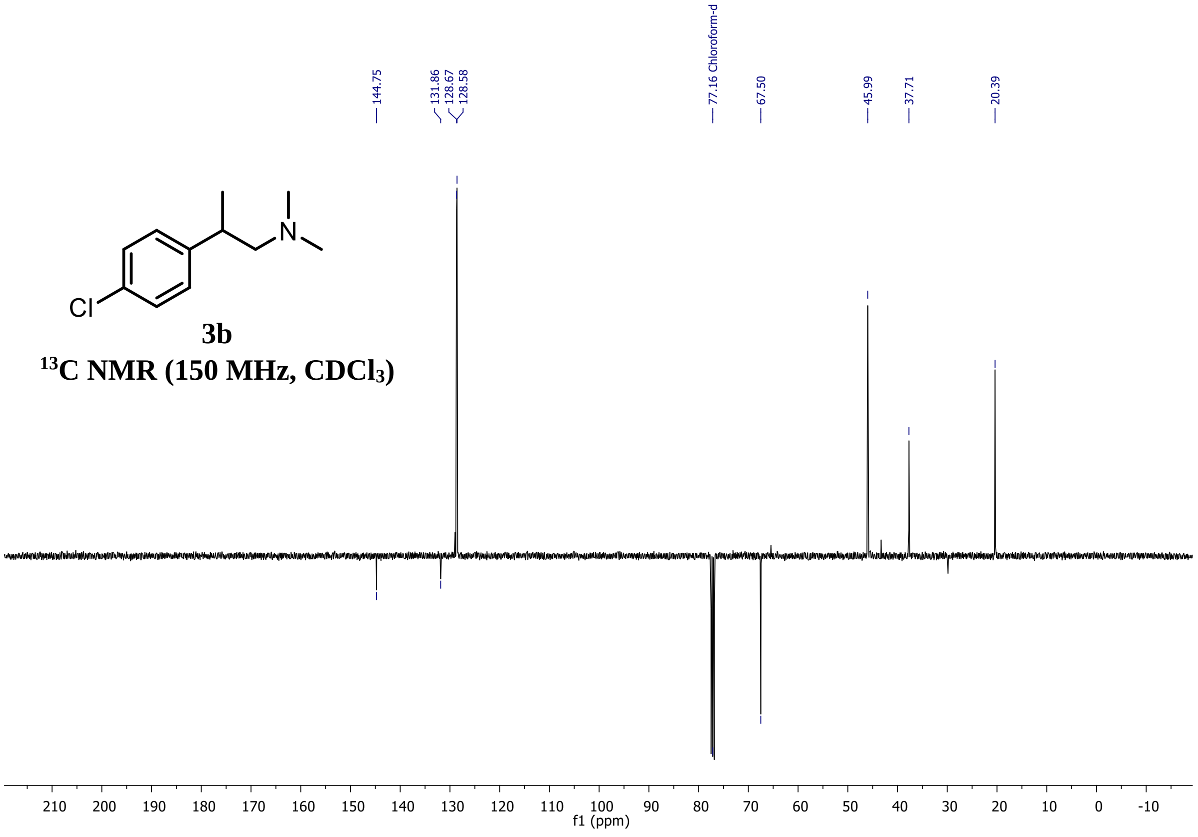
Cl
N
3b
¹³C NMR (150 MHz, CDCl₃)
144.75
131.86 128.67 128.58
77.16 Chloroform-d
67.50
45.99
37.71
20.39
210 200 190 180 170 160 150 140 130 120 110 100 90 80 70 60 50 40 30 20 10 0 -10
f1 (ppm)

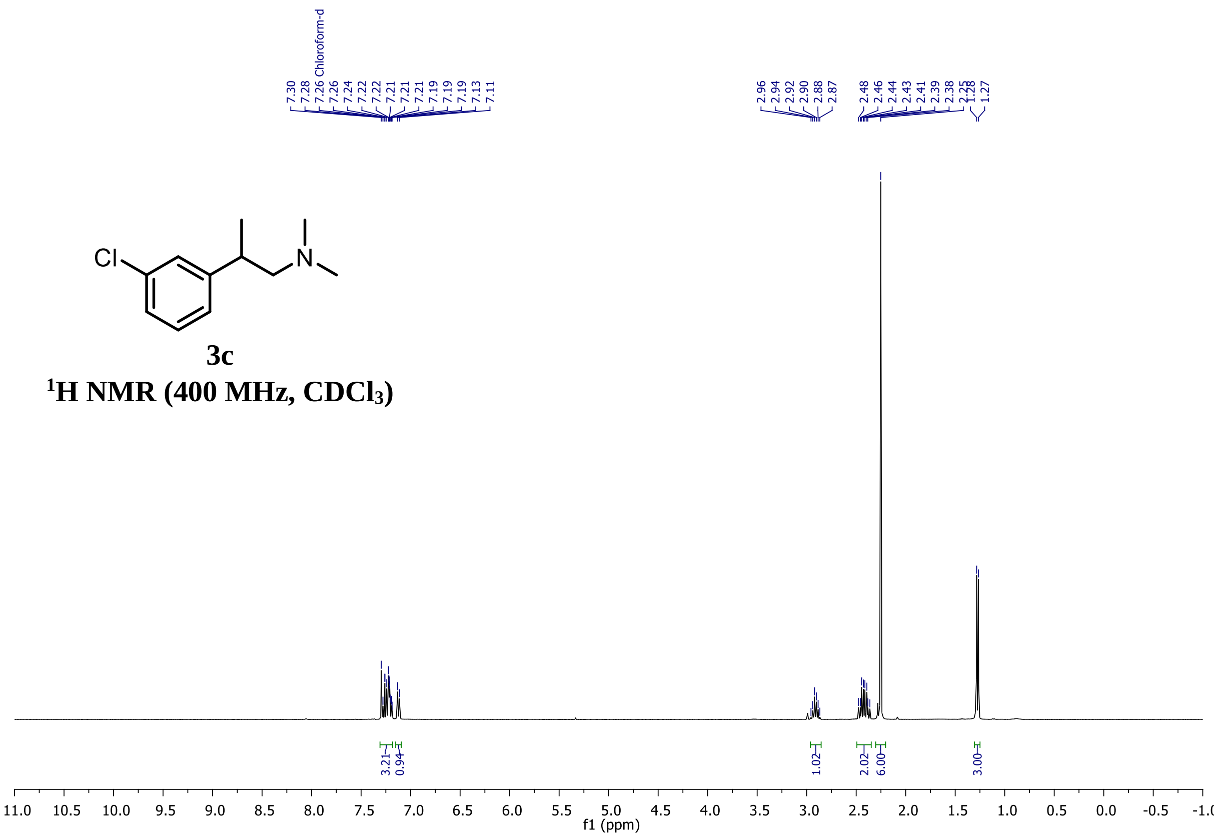

3c

^{1}H NMR (400 MHz, $CDCl_3$)

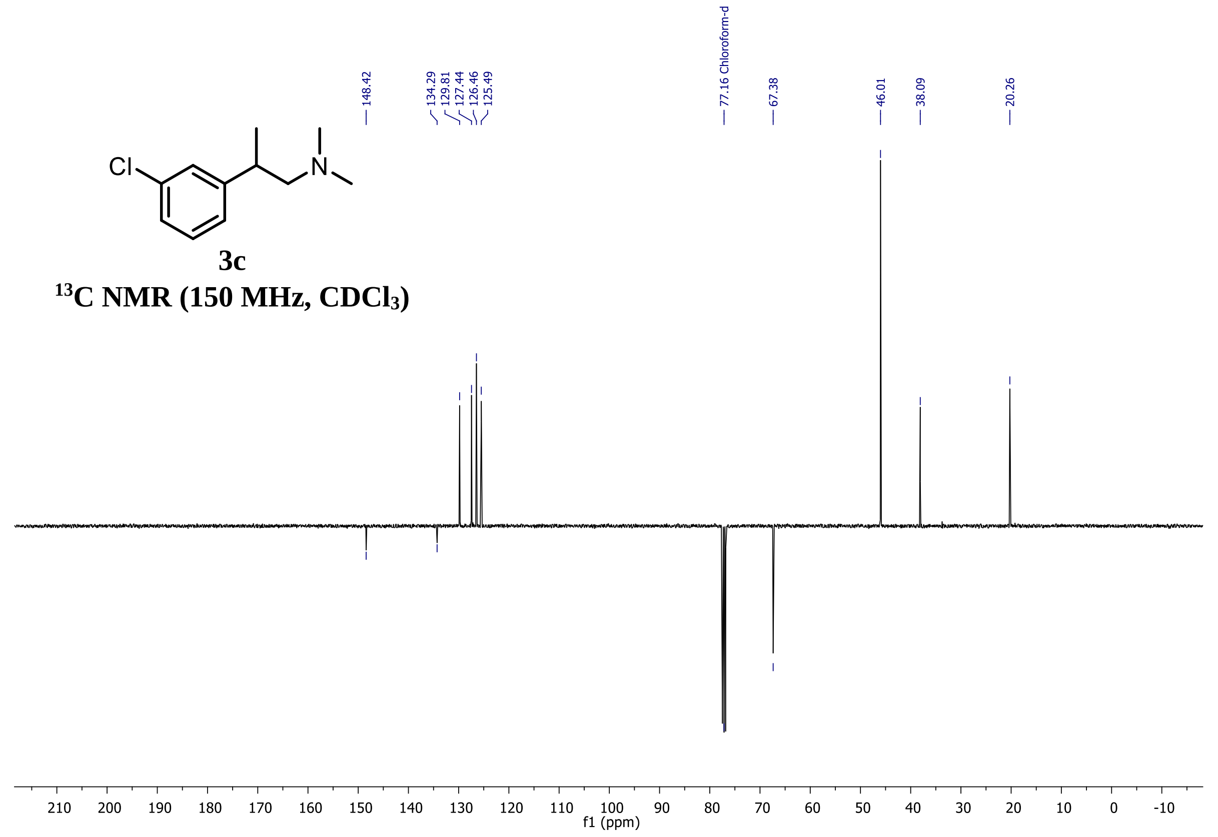

3c

^{13}C NMR (150 MHz, $CDCl_3$)

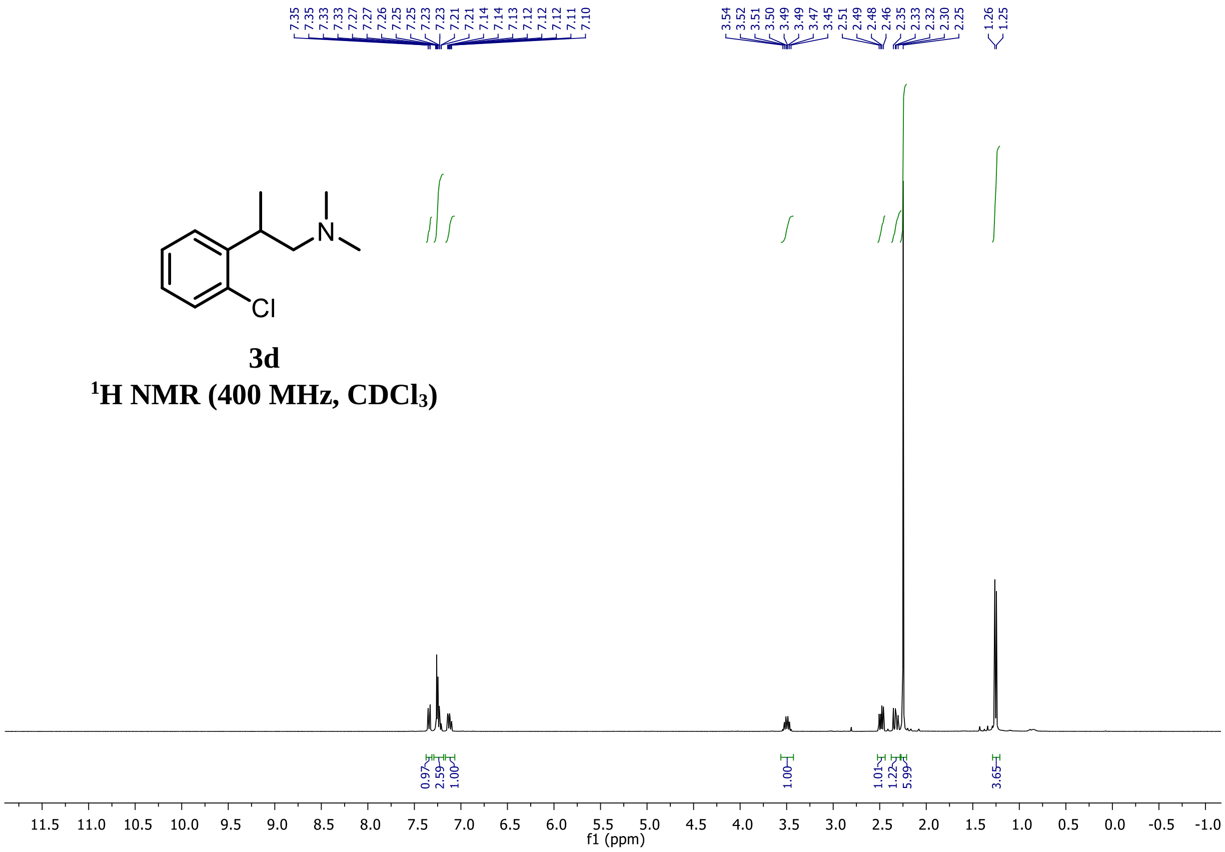
3d
^{1}H NMR (400 MHz, $CDCl_3$)
Cl
N
f1 (ppm)

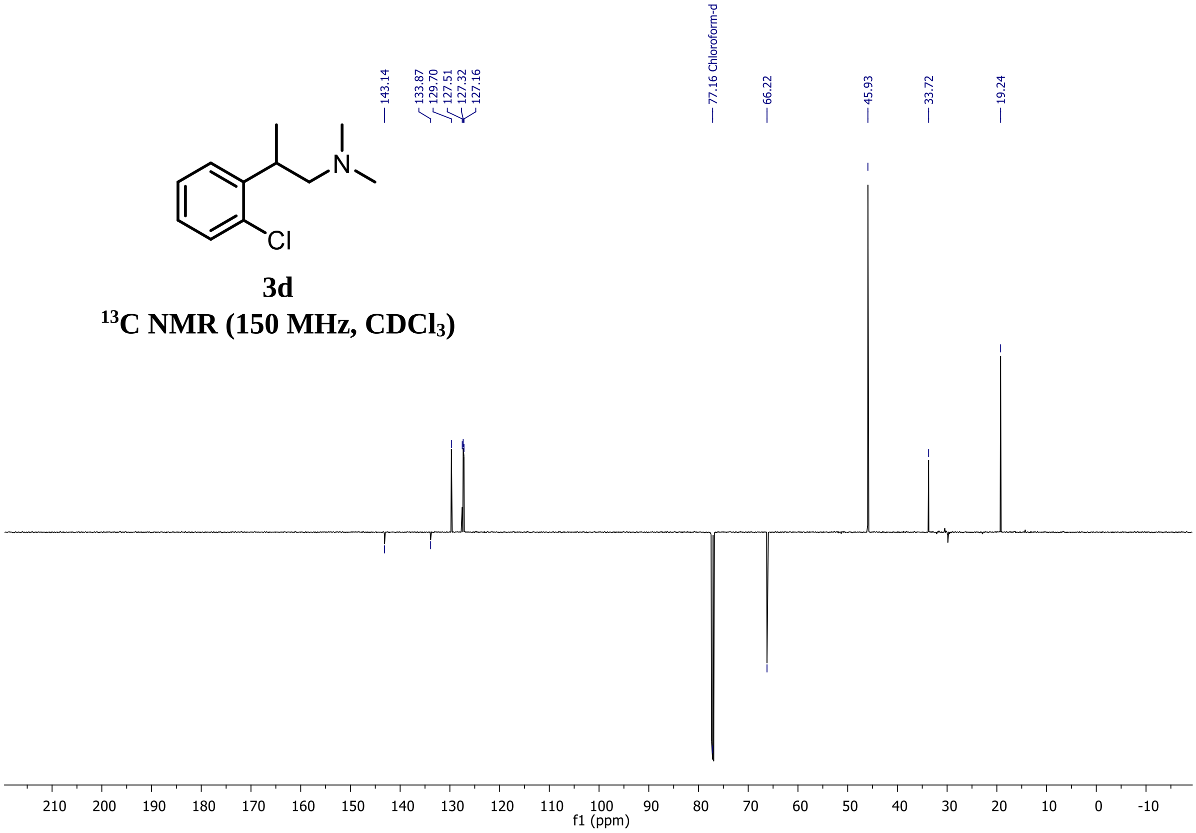
3d
^{13}C NMR (150 MHz, $CDCl_3$)
Cl
N
77.16 Chloroform-d
f1 (ppm)

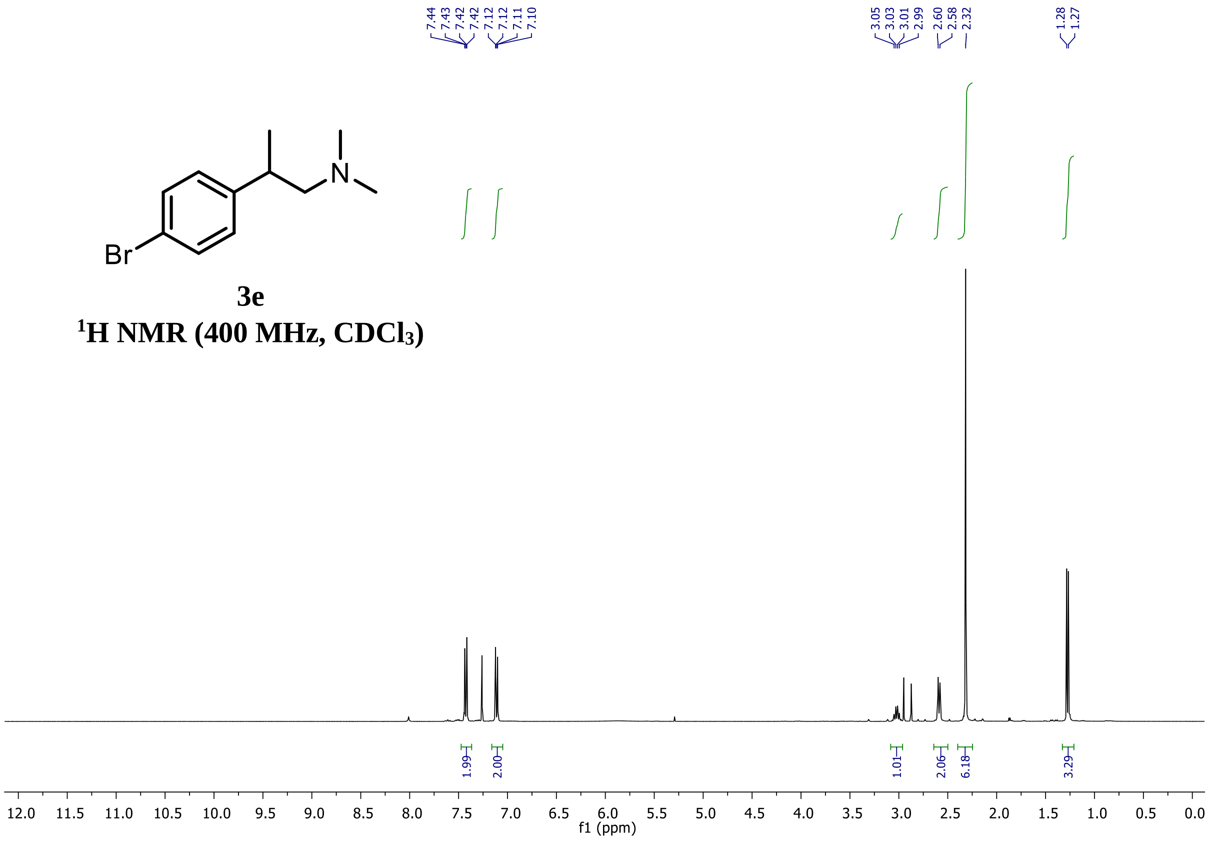

3e

^{1}H NMR (400 MHz, CDCl$_3$)

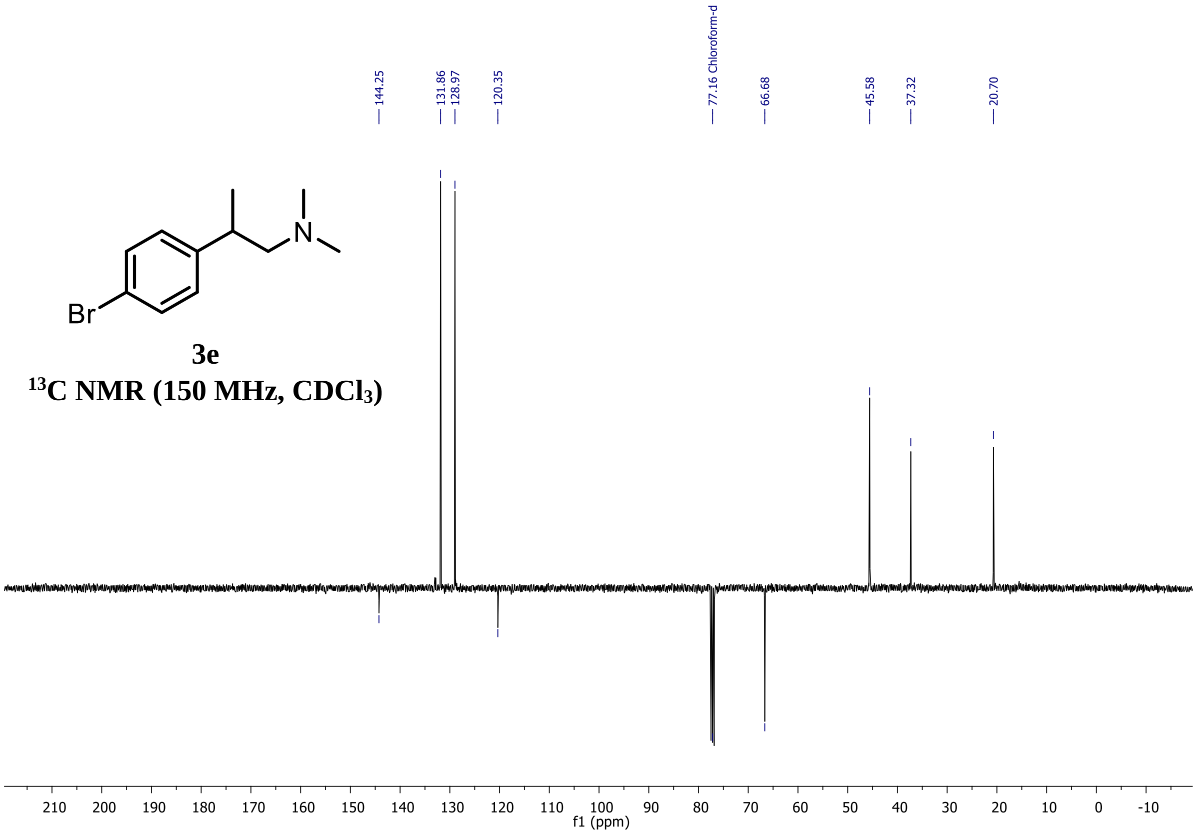

3e

^{13}C NMR (150 MHz, CDCl$_3$)

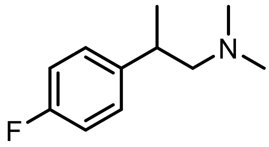

3f

^{1}H NMR (400 MHz, CDCl$_3$)

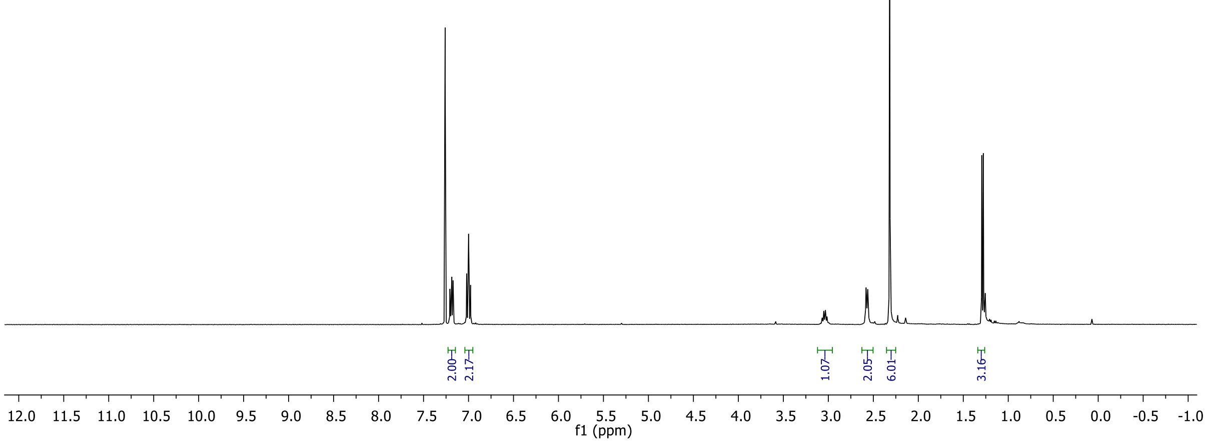

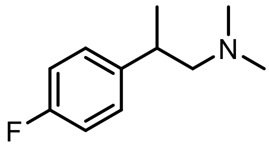

3f

^{13}C NMR (150 MHz, CDCl$_3$)

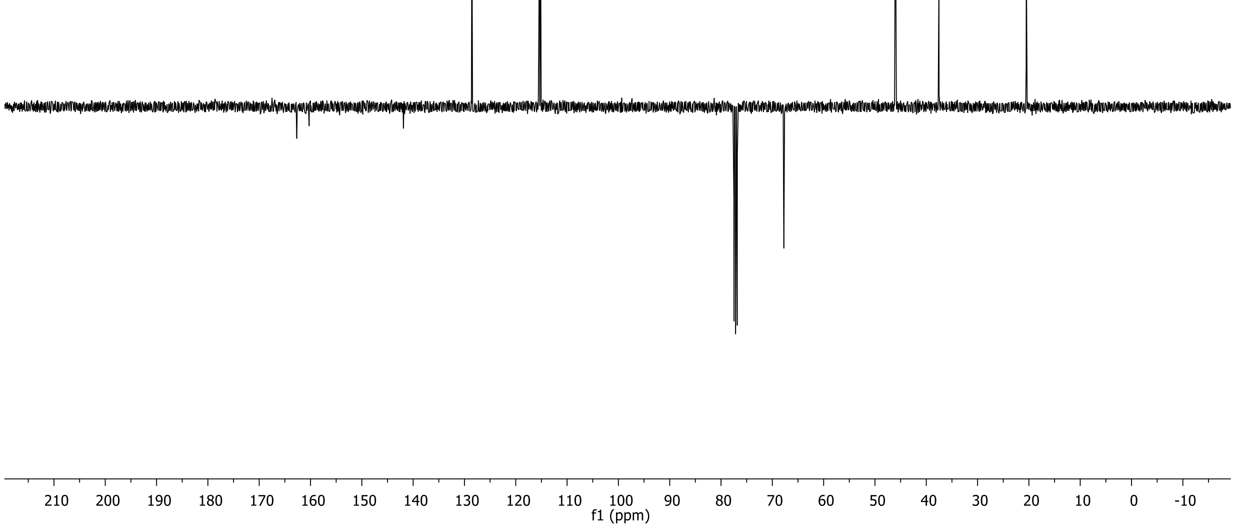

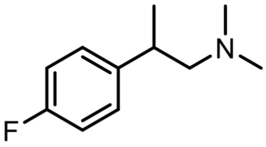

3f

^{19}F NMR (659 MHz, CDCl$_3$)

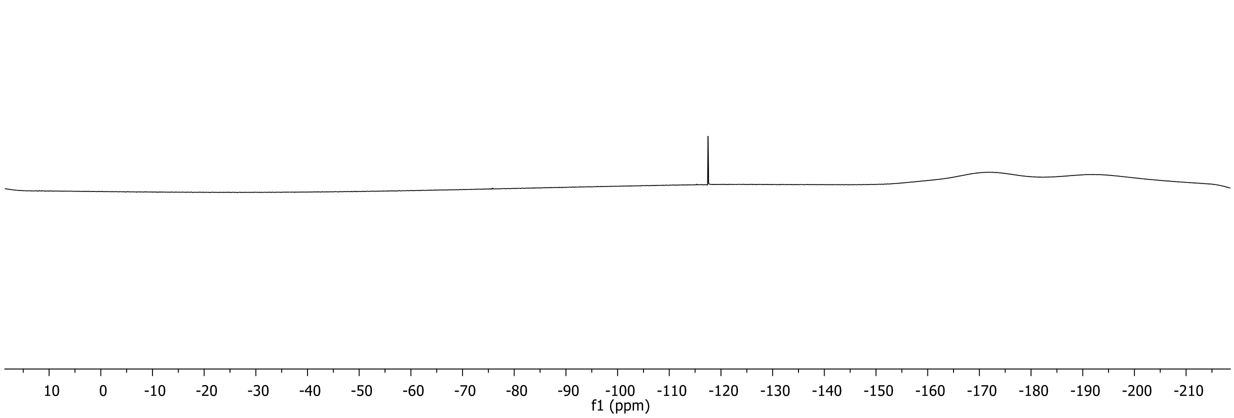

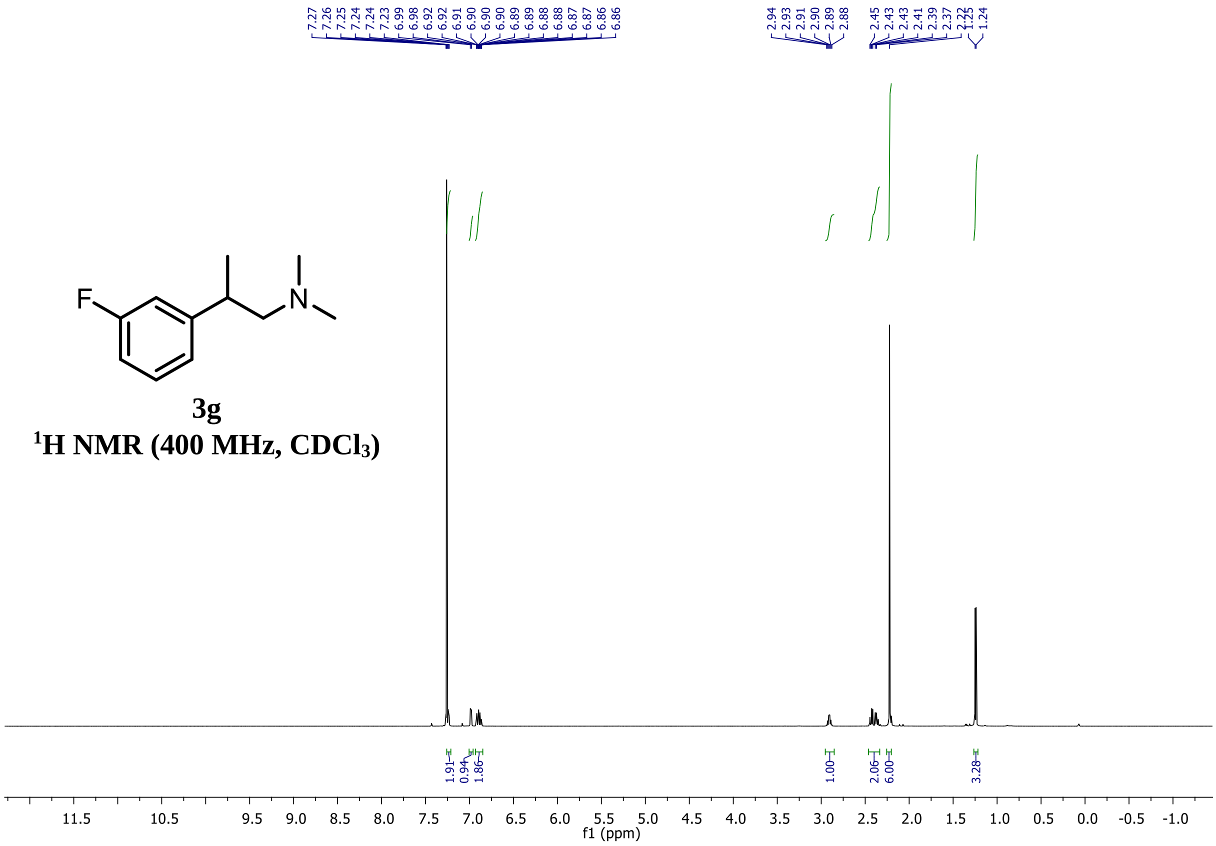

3g

^{1}H NMR (400 MHz, CDCl$_3$)

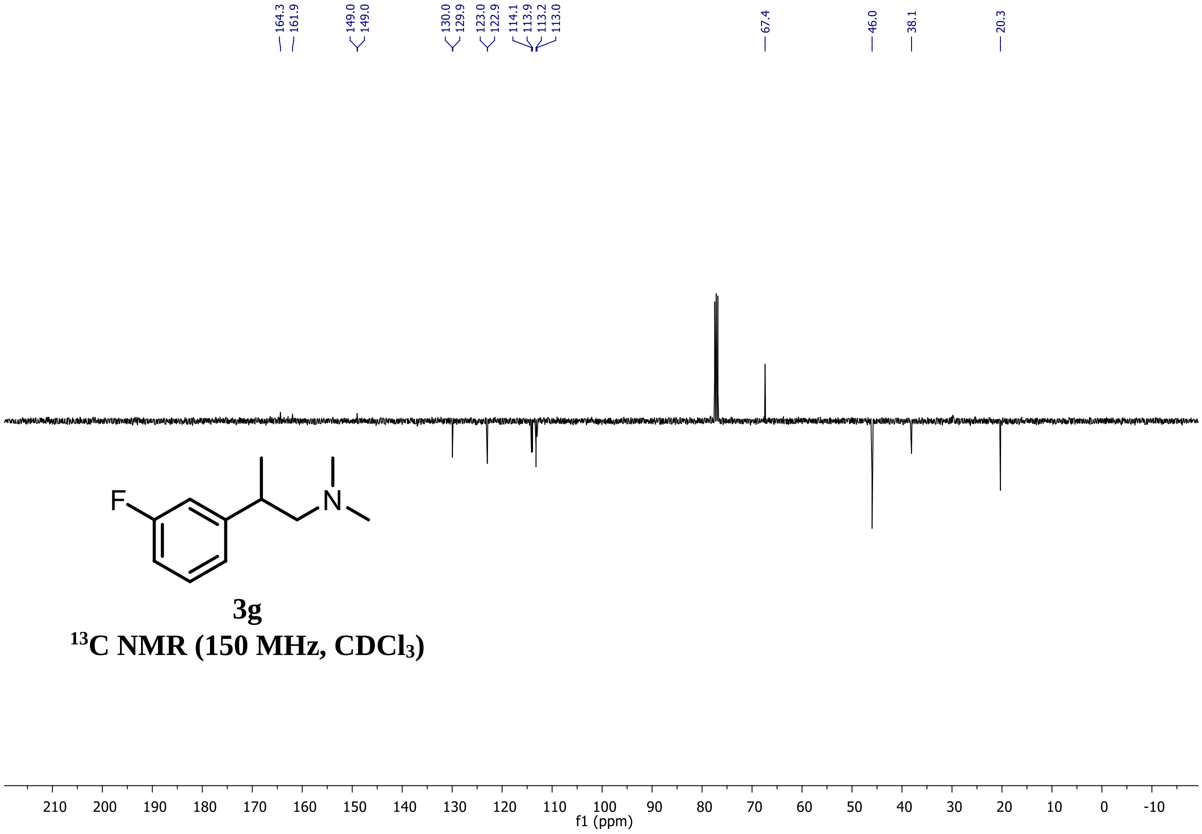

3g

^{13}C NMR (150 MHz, CDCl$_3$)

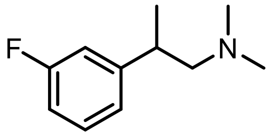

3g

^{19}F NMR (659 MHz, CDCl$_3$)

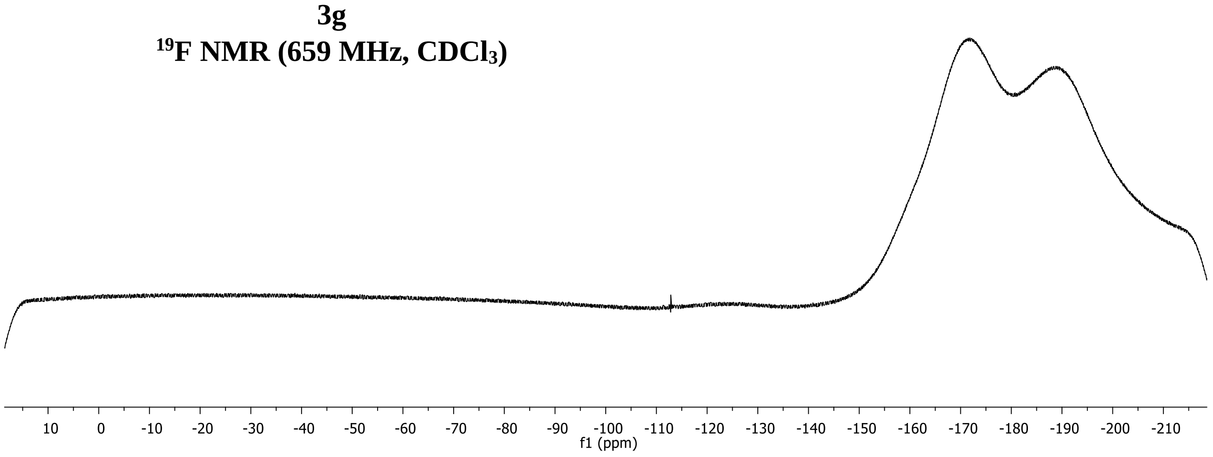

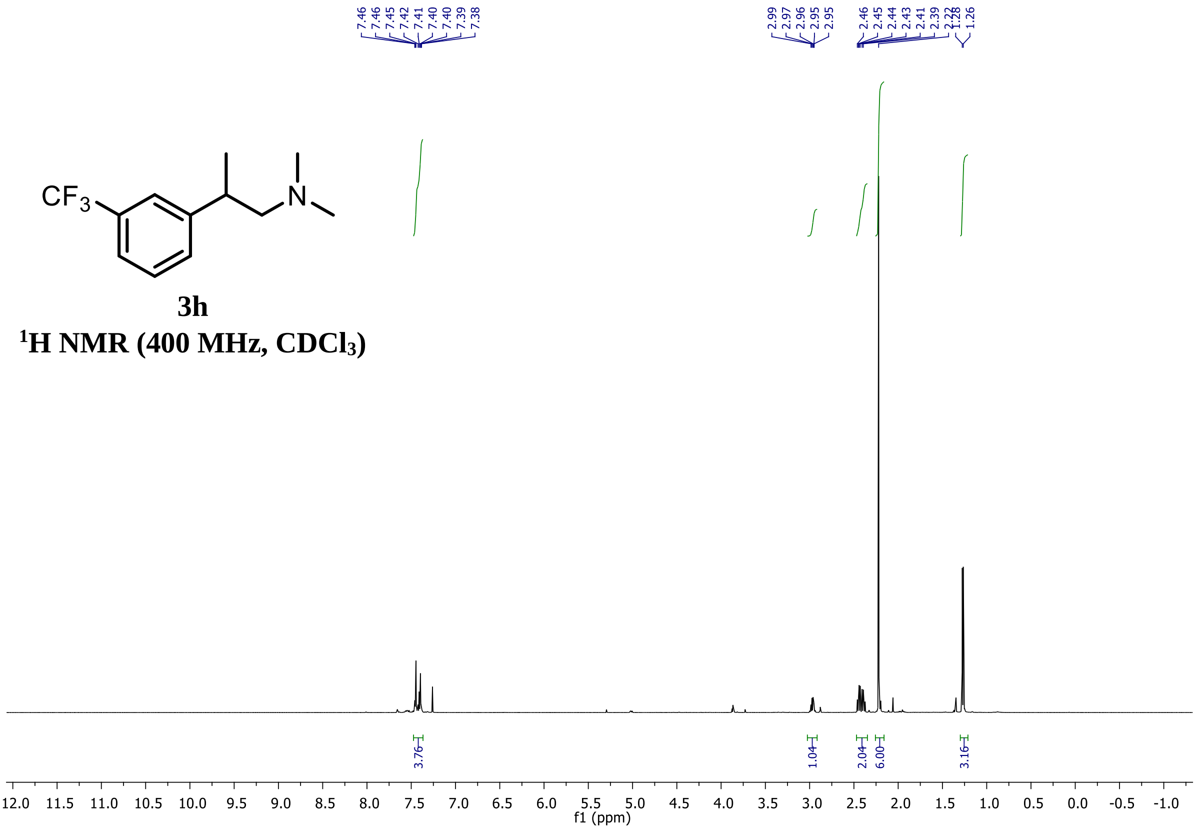

3h

^{1}H NMR (400 MHz, $CDCl_3$)

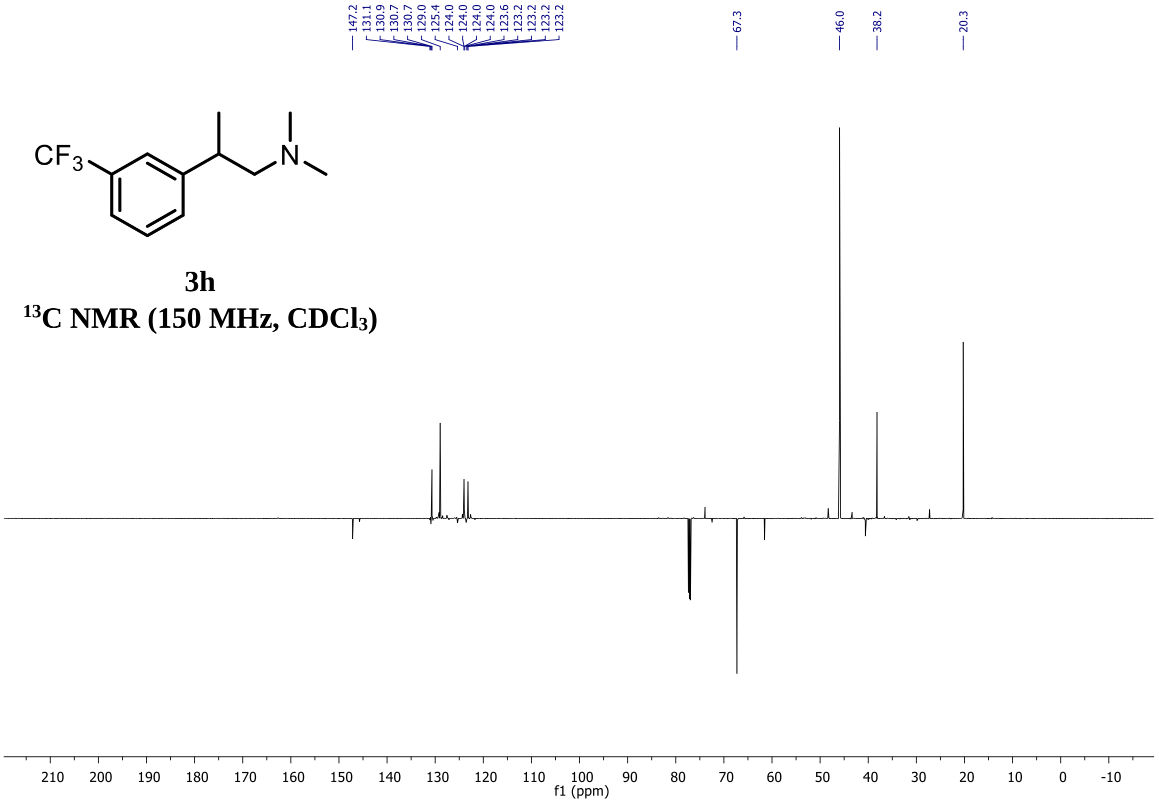

3h

^{13}C NMR (150 MHz, $CDCl_3$)

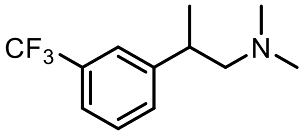

3h

^{19}F NMR (659 MHz, $CDCl_3$)

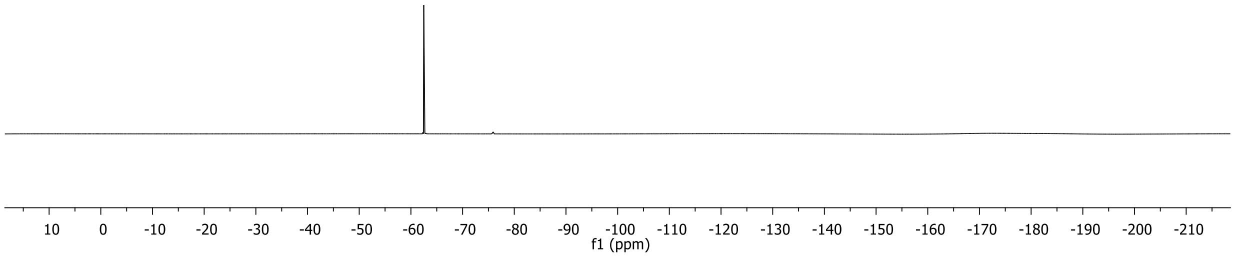

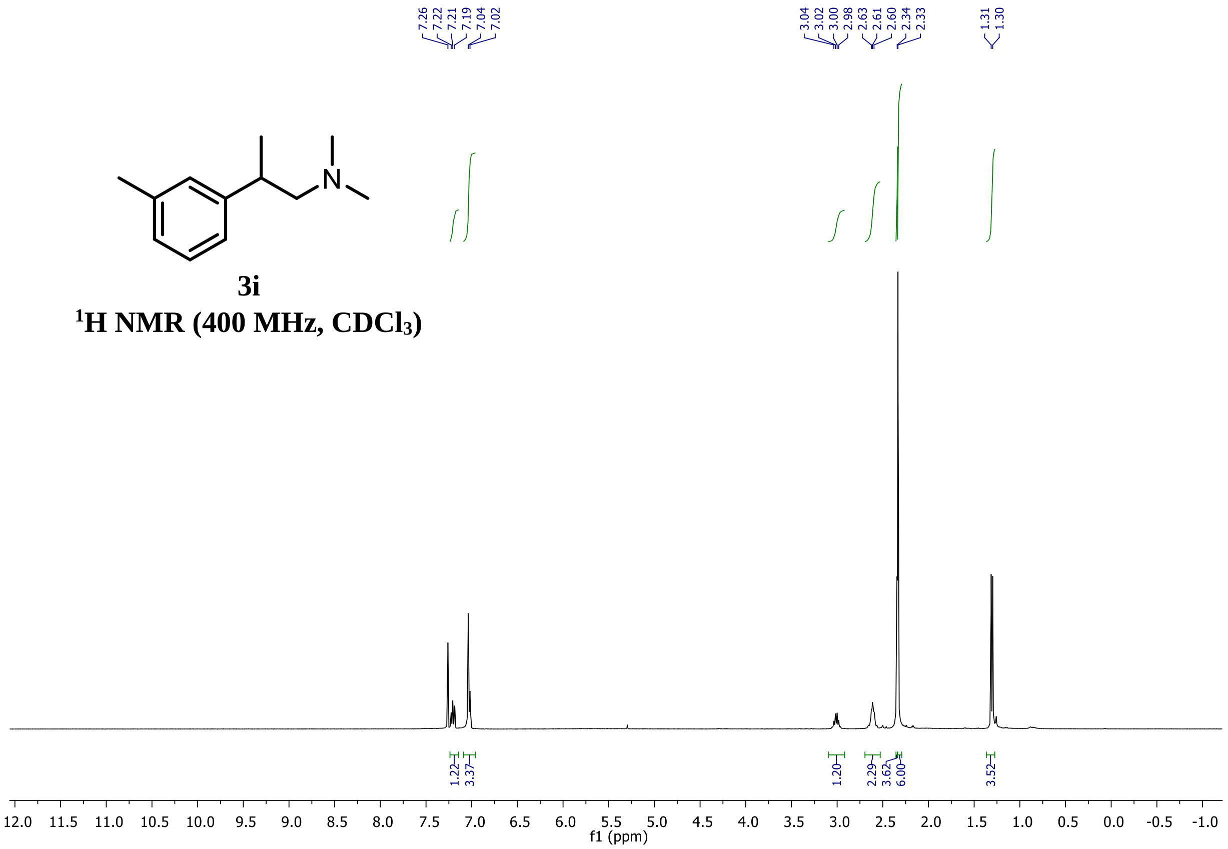

3i

^{1}H NMR (400 MHz, CDCl$_3$)

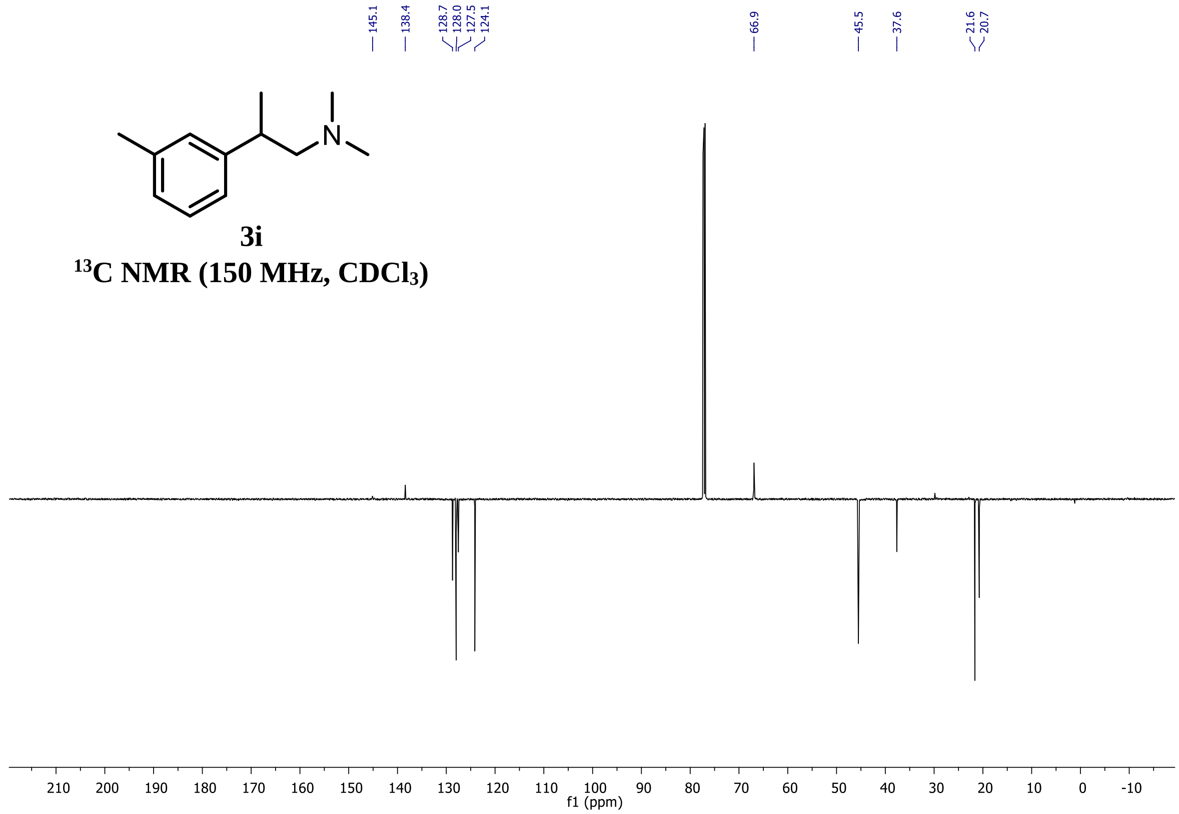

3i

^{13}C NMR (150 MHz, CDCl$_3$)

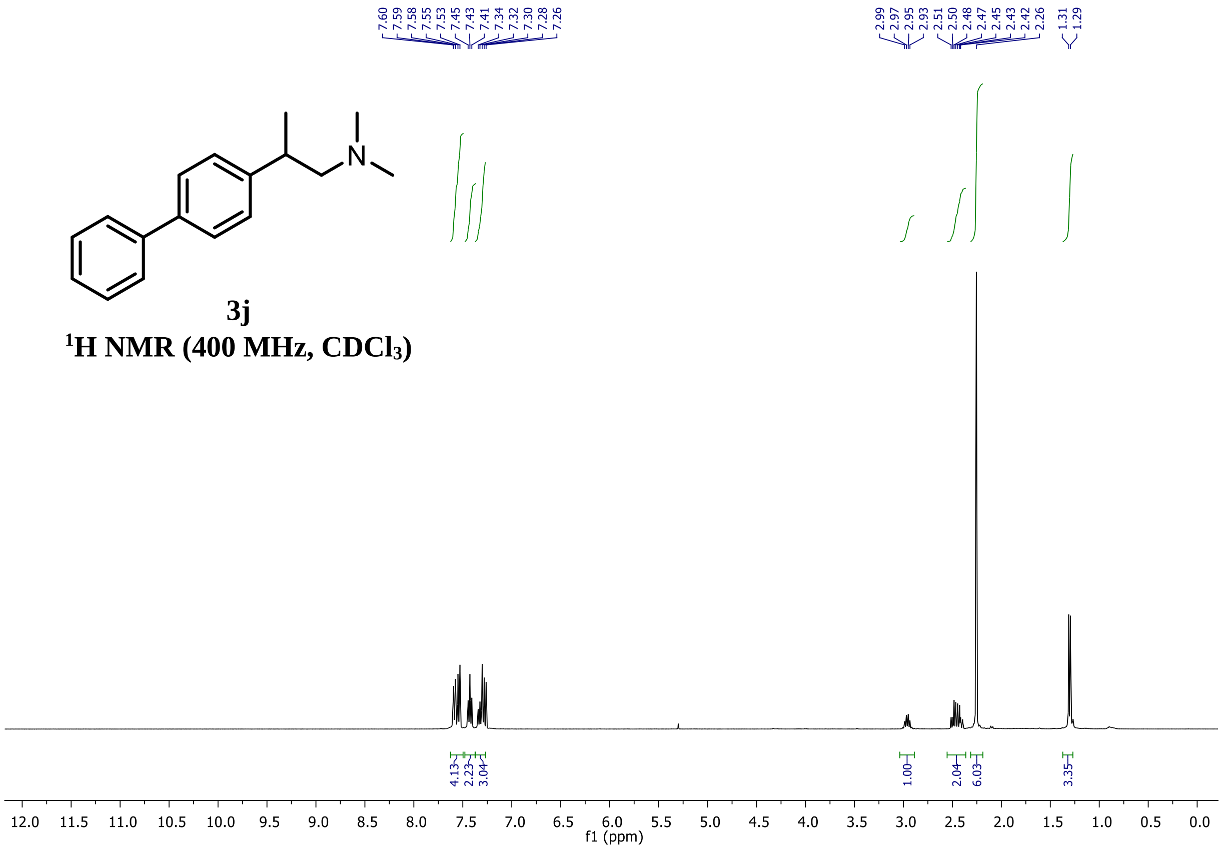

3j

^{1}H NMR (400 MHz, CDCl$_3$)

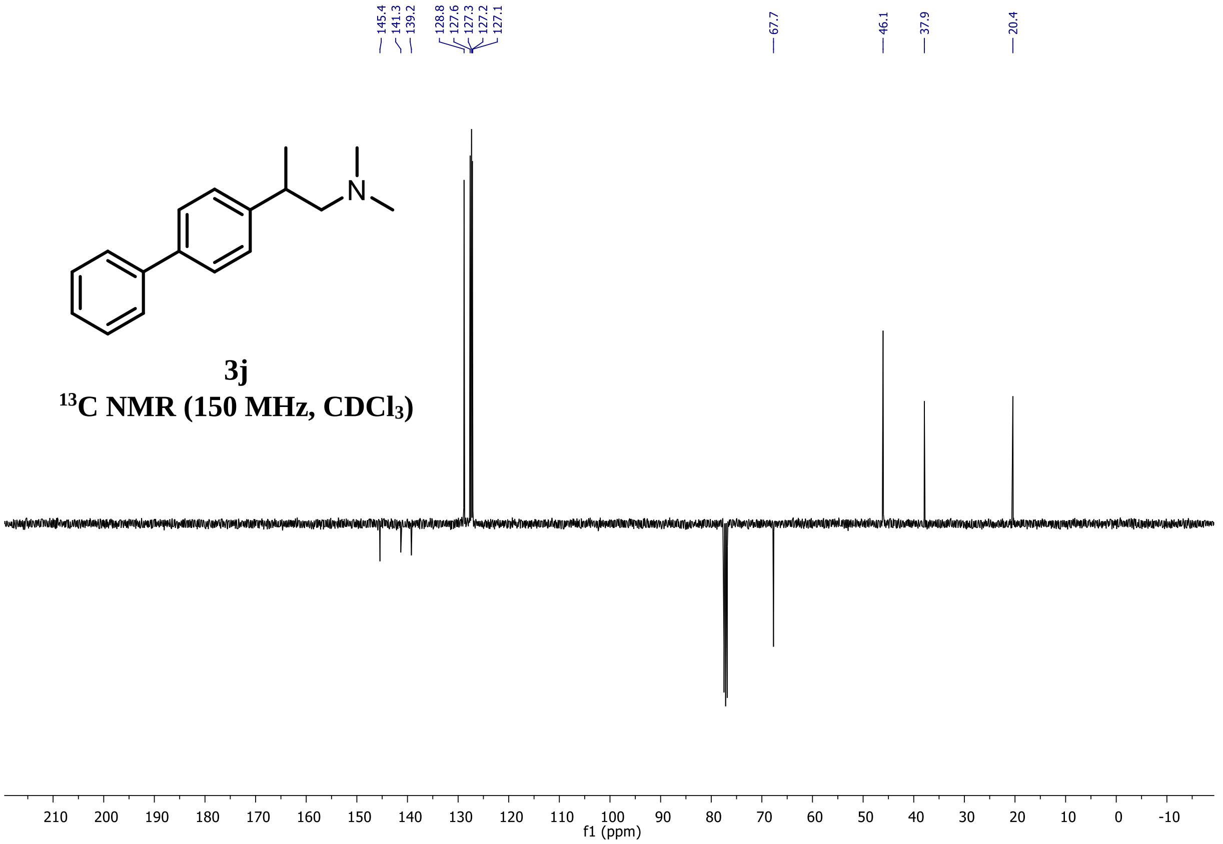

3j

^{13}C NMR (150 MHz, CDCl$_3$)

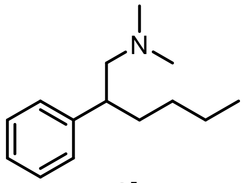

3k

^{1}H NMR (400 MHz, CDCl$_3$)

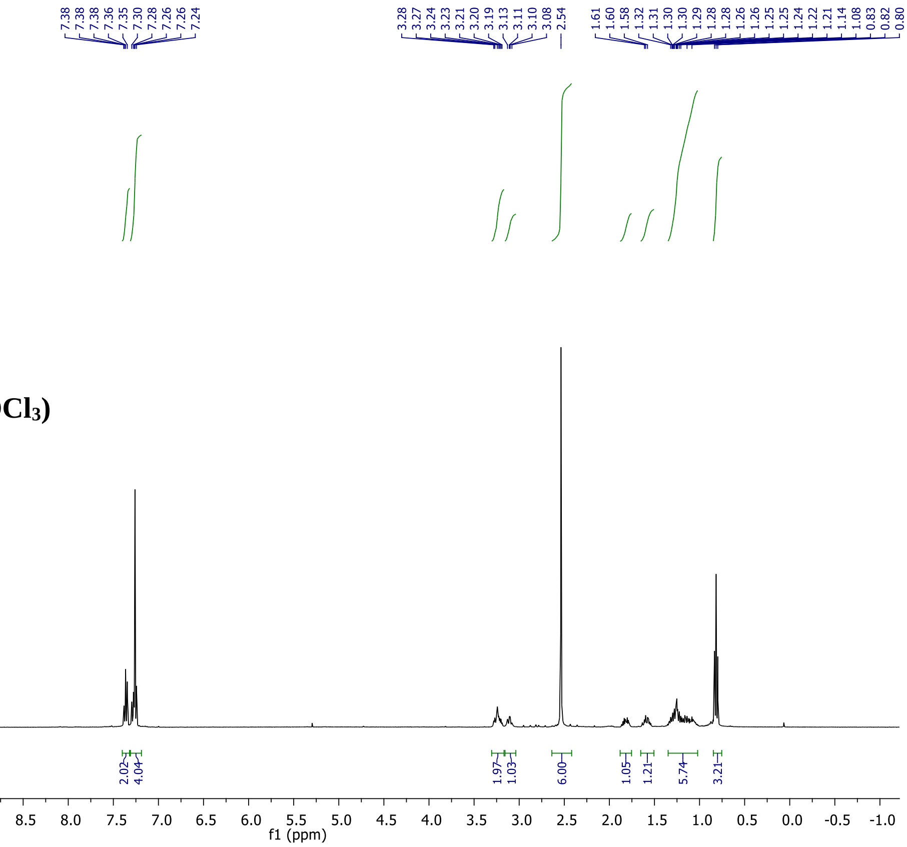

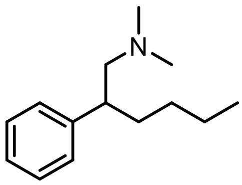

3k

^{13}C NMR (150 MHz, CDCl$_3$)

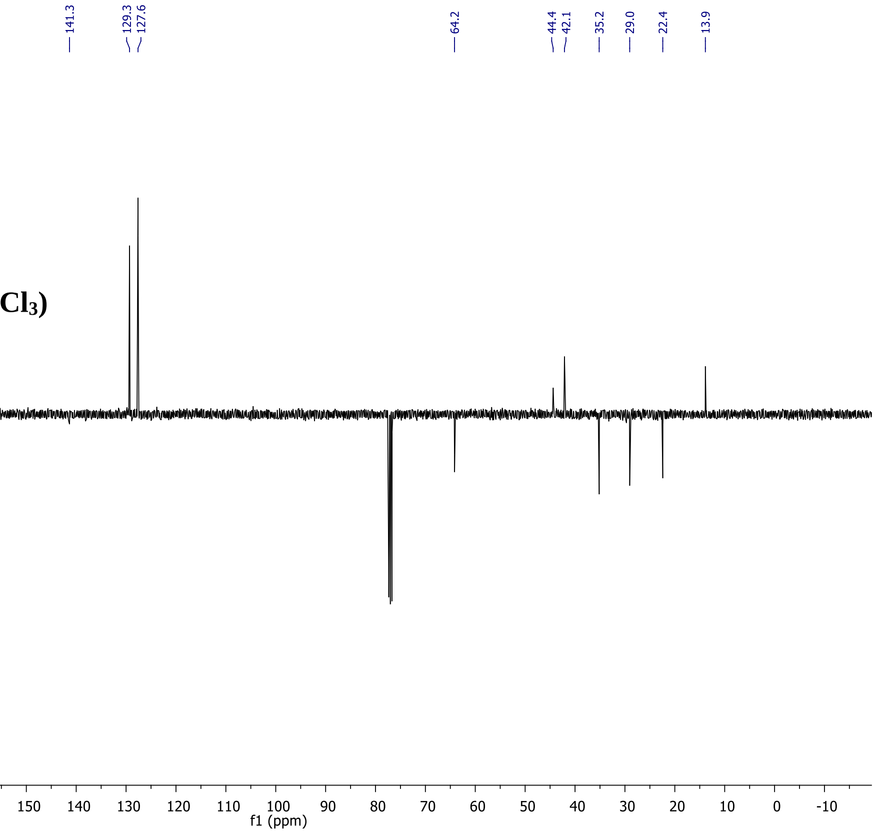

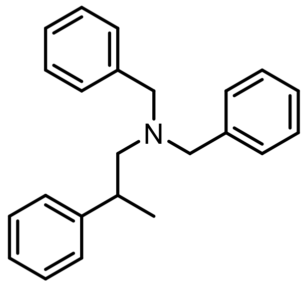

3l

^{1}H NMR (400 MHz, $CDCl_3$)

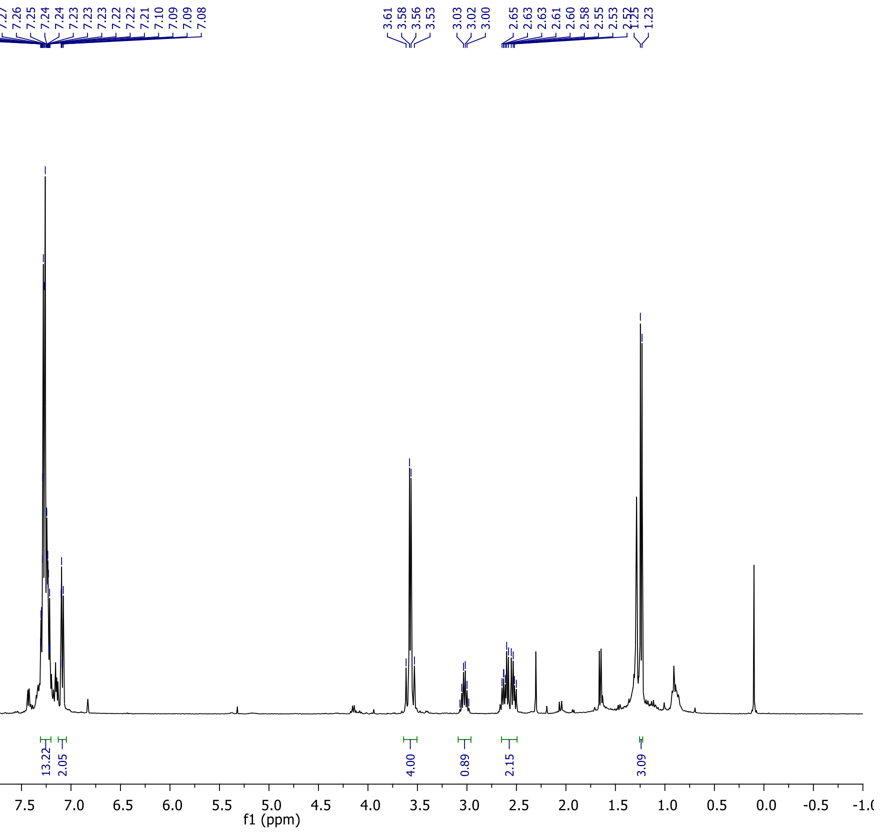

3m
^{1}H NMR (400 MHz, $CDCl_3$)

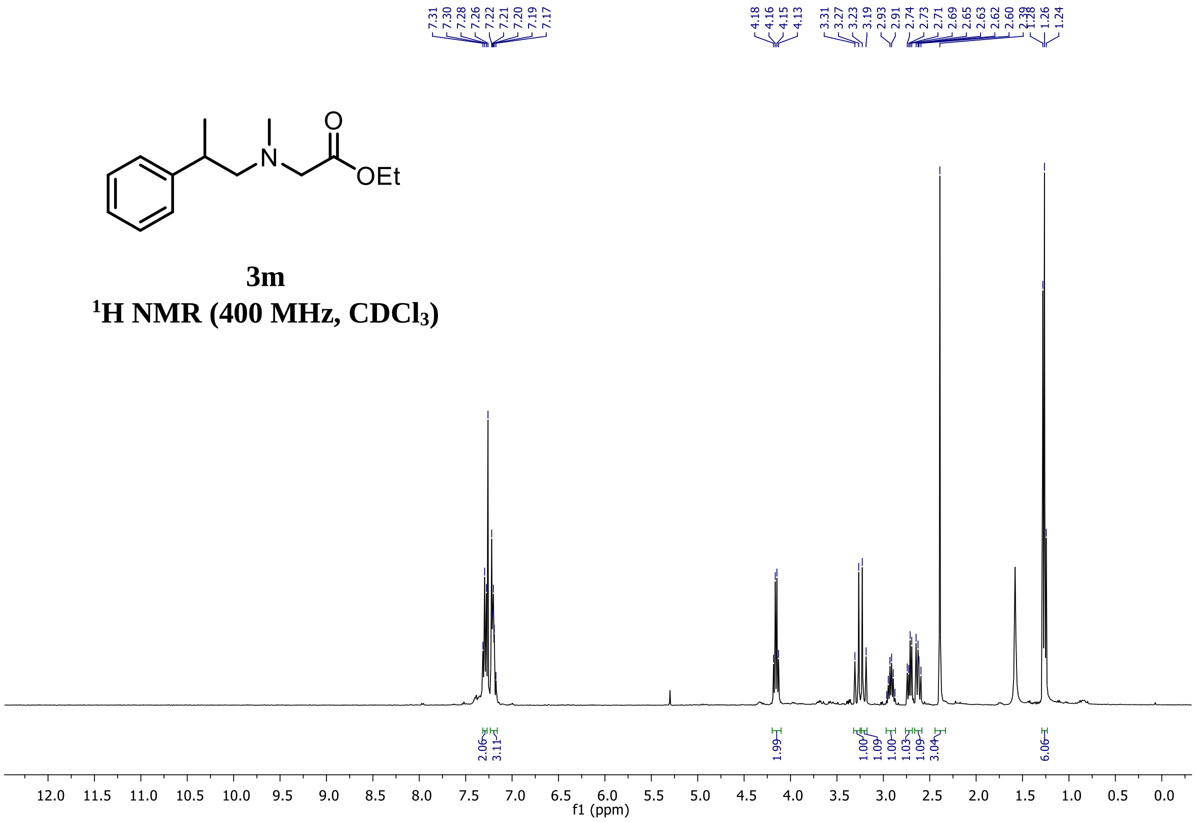

3m
^{13}C NMR (100 MHz, $CDCl_3$)

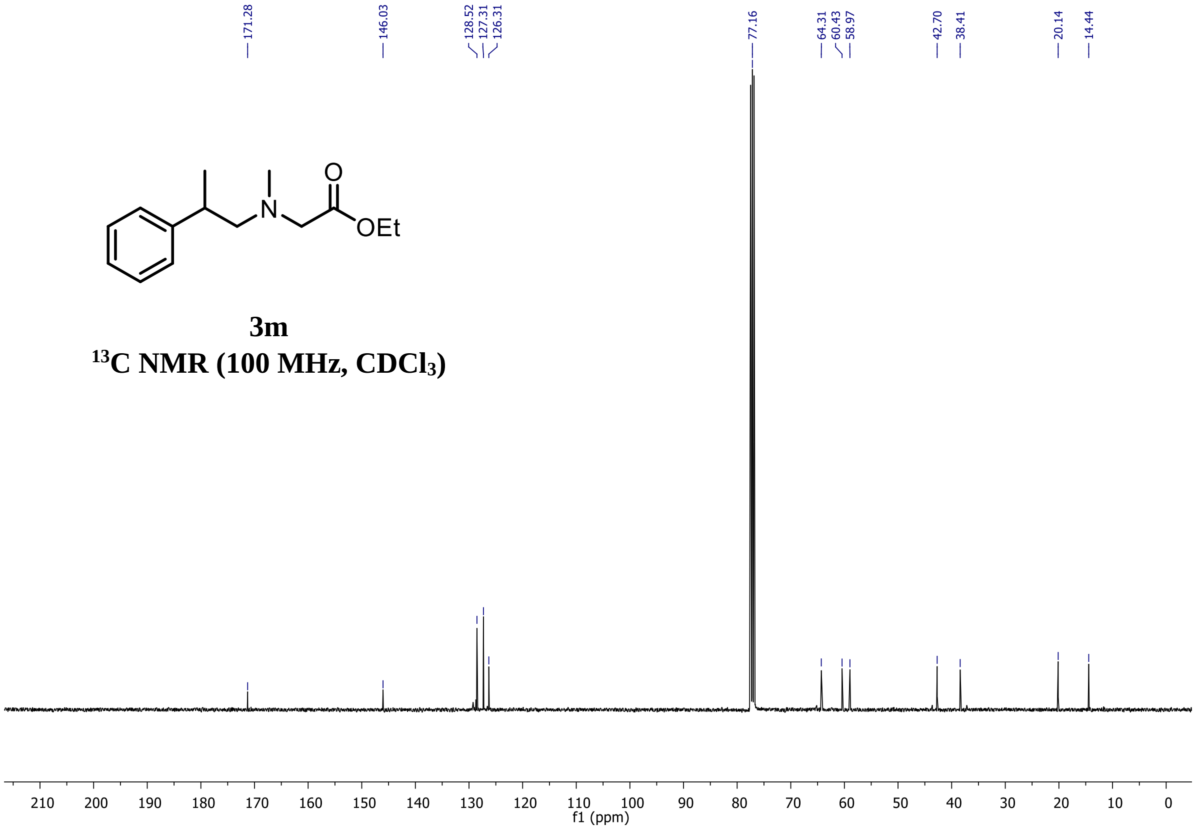

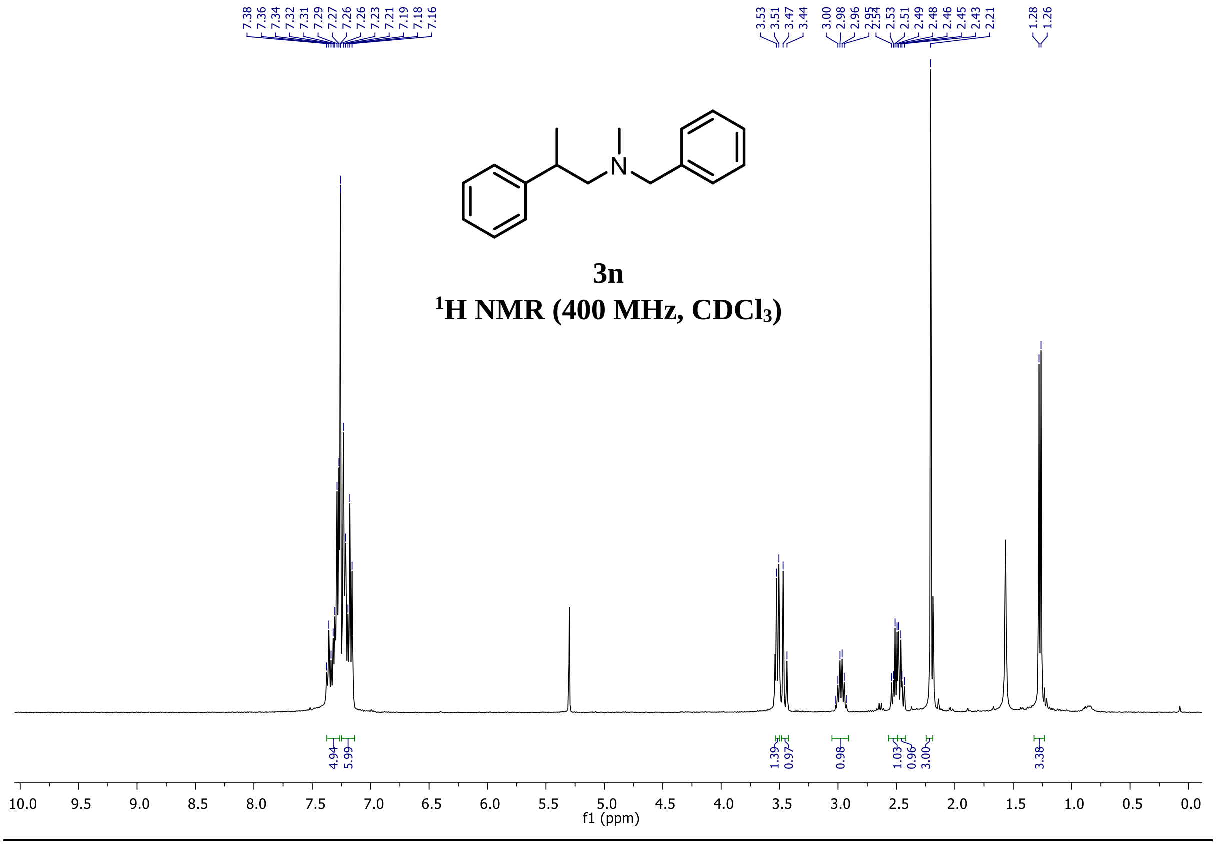

3n

^{1}H NMR (400 MHz, $CDCl_3$)

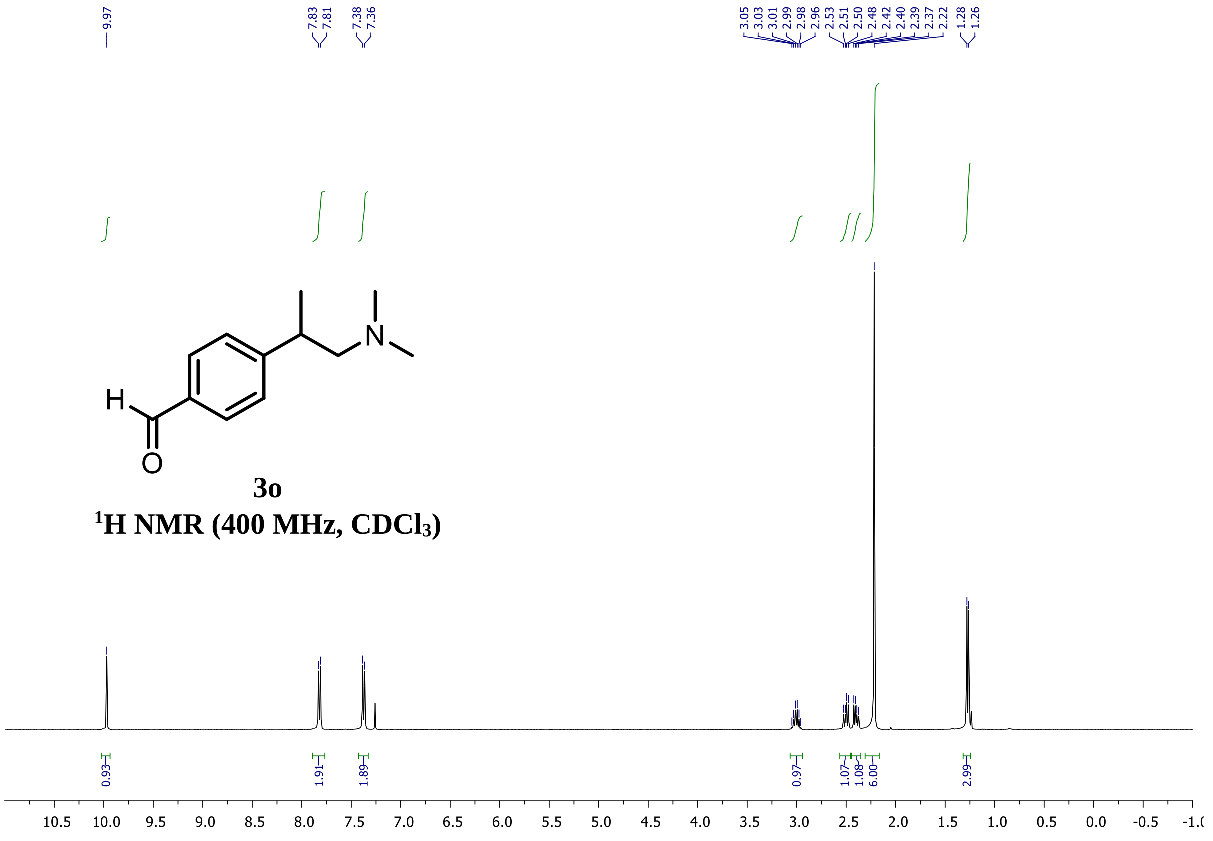

9.97
7.83
7.81
7.38
7.36
3.05
3.03
3.01
2.99
2.98
2.96
2.53
2.51
2.50
2.48
2.42
2.40
2.39
2.37
2.22
1.28
1.26
H
O
N
3o
¹H NMR (400 MHz, CDCl₃)
0.93
1.91
1.89
0.97
1.07
1.08
6.00
2.99

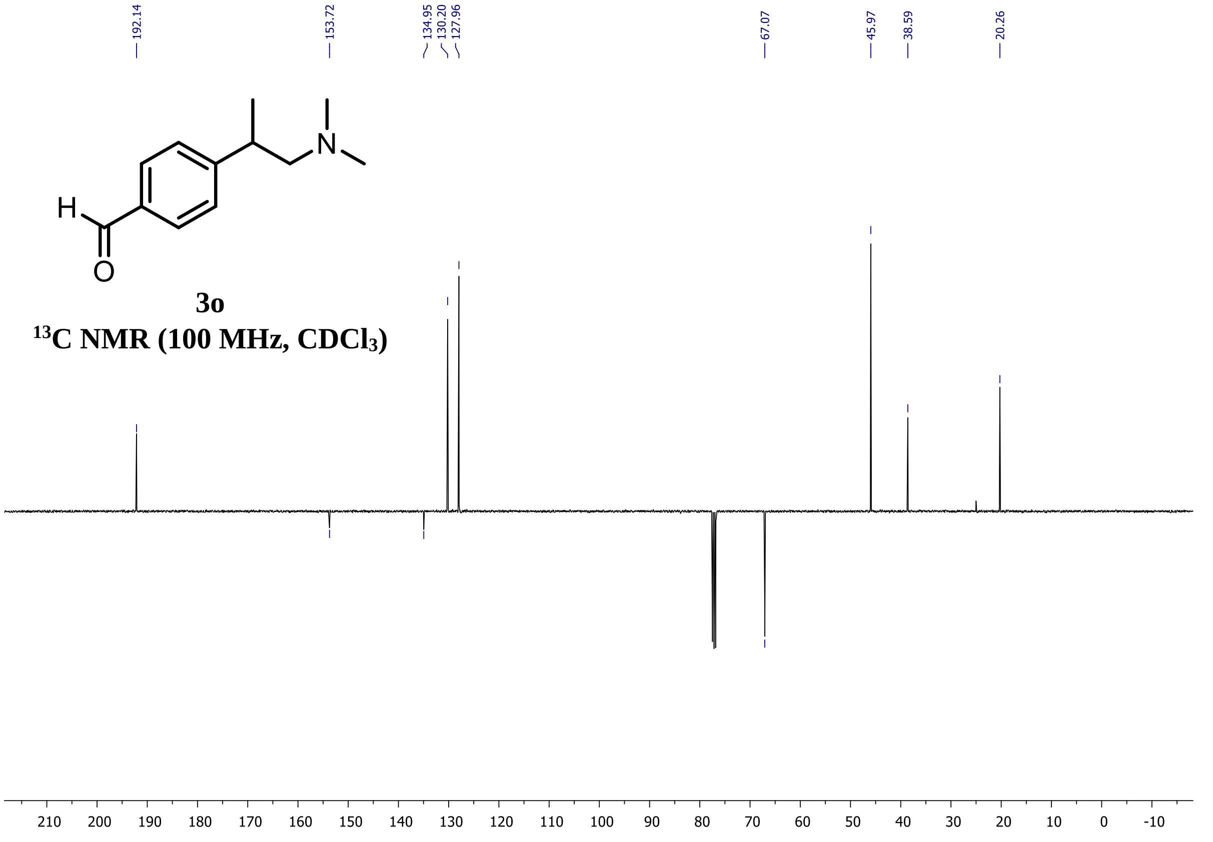

192.14
153.72
134.95
130.20
127.96
67.07
45.97
38.59
20.26
H
O
N
3o
¹³C NMR (100 MHz, CDCl₃)

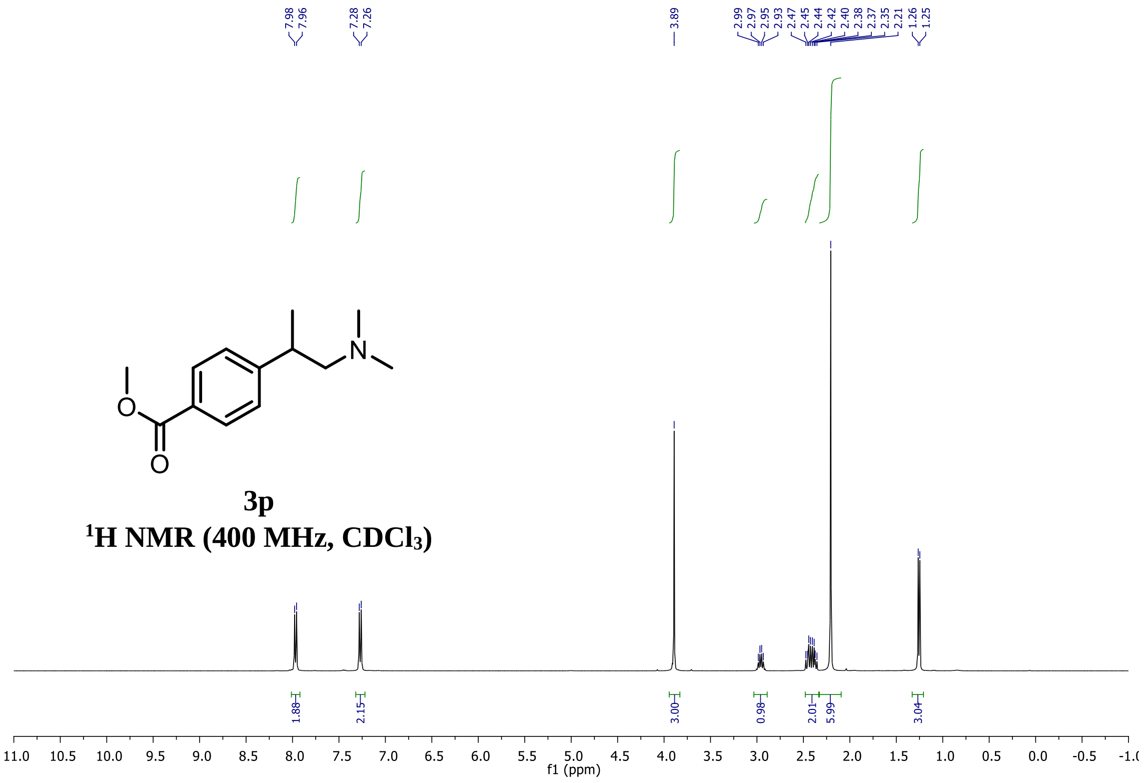

3p
^{1}H NMR (400 MHz, CDCl$_3$)
7.98
7.96
7.28
7.26
3.89
2.99
2.97
2.95
2.93
2.47
2.45
2.44
2.42
2.40
2.38
2.37
2.35
2.21
1.26
1.25
1.88
2.15
3.00
0.98
2.01
5.99
3.04
f1 (ppm)

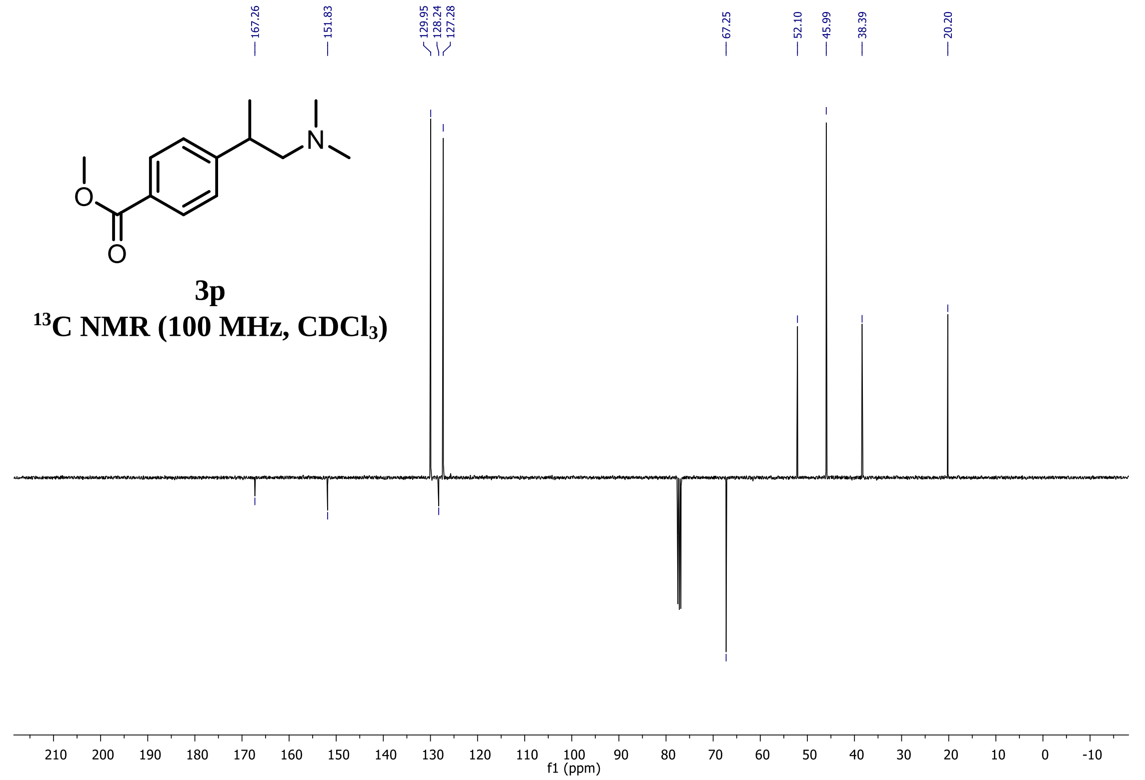

3p
^{13}C NMR (100 MHz, CDCl$_3$)
167.26
151.83
129.95
128.24
127.28
67.25
52.10
45.99
38.39
20.20
f1 (ppm)

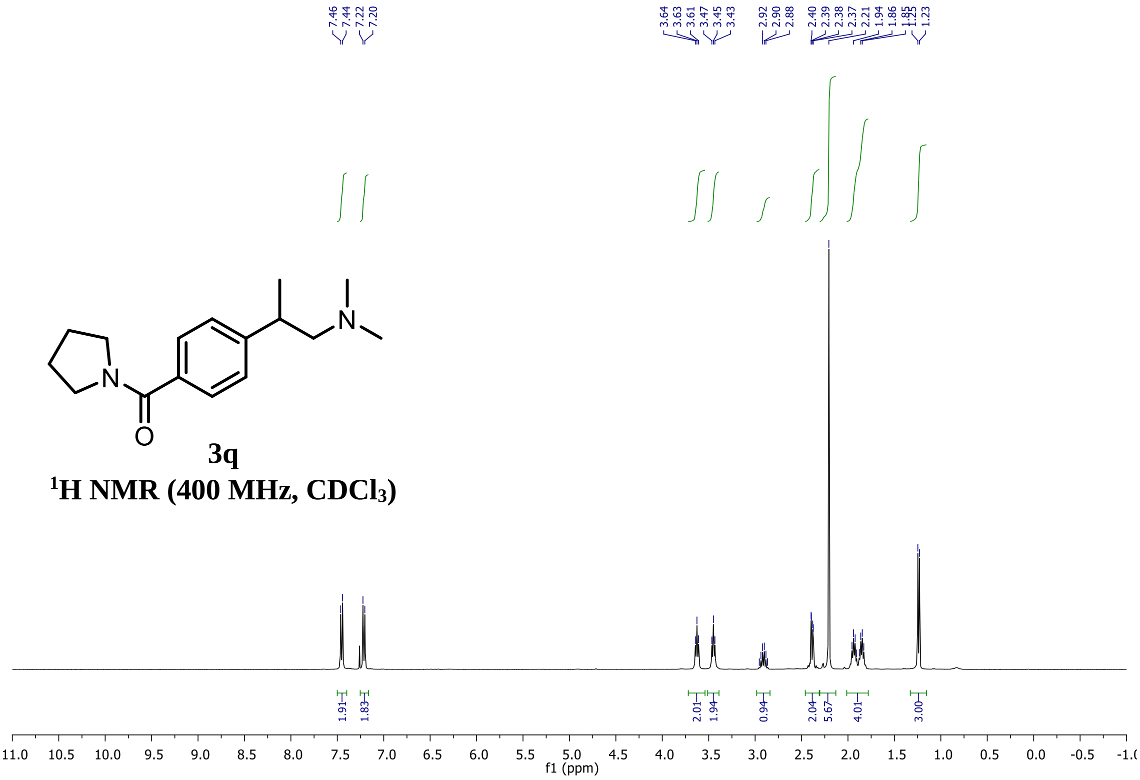

3q

^{1}H NMR (400 MHz, $CDCl_3$)

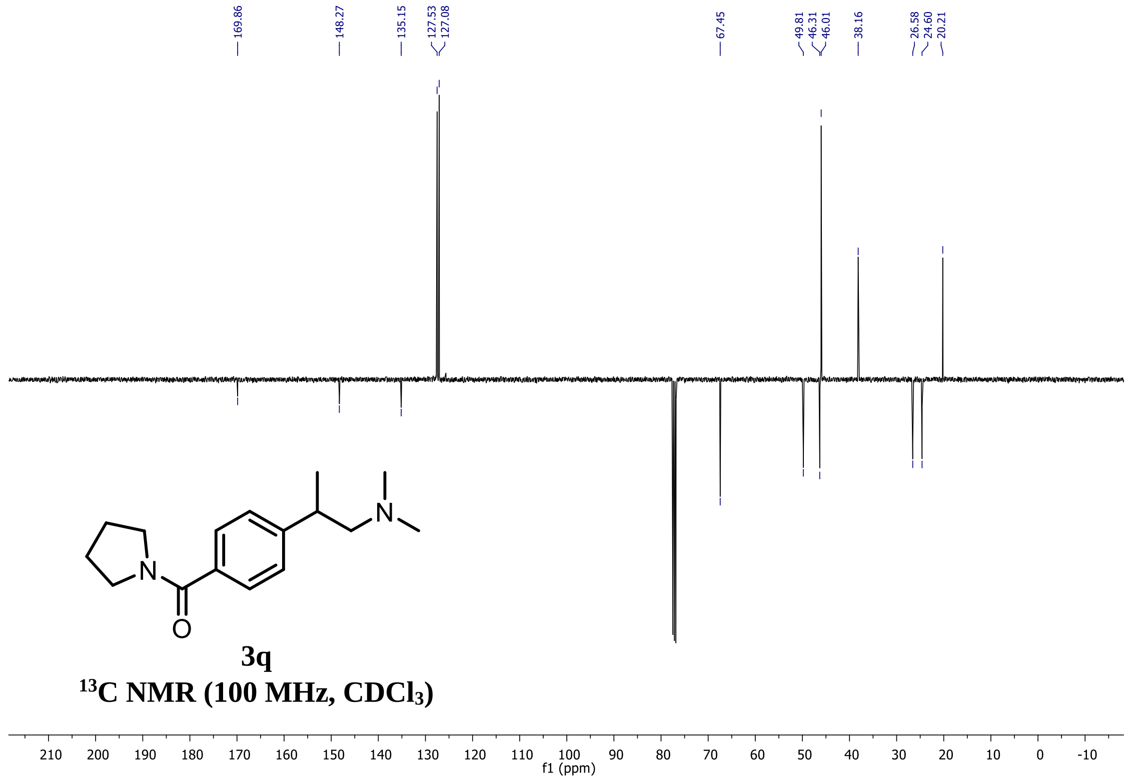

3q

^{13}C NMR (100 MHz, $CDCl_3$)

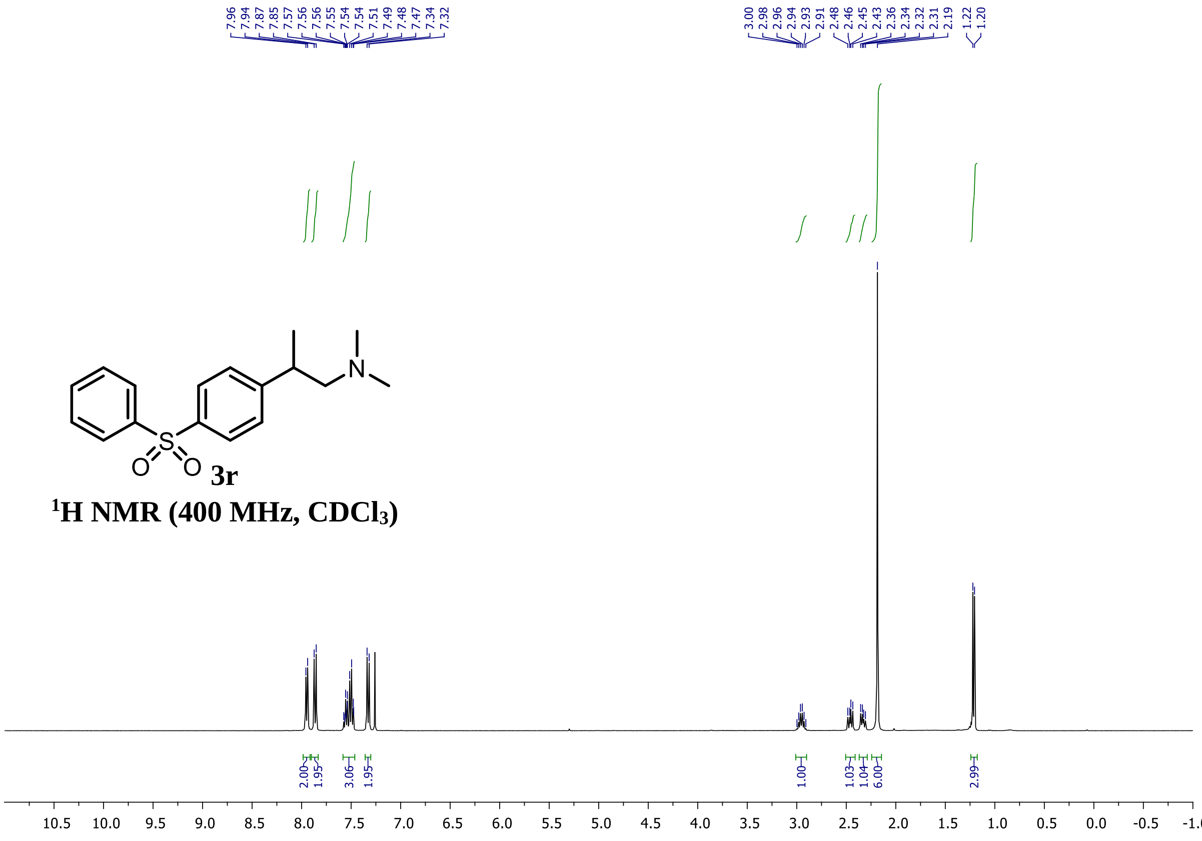
7.96
7.94
7.87
7.85
7.57
7.56
7.56
7.55
7.54
7.54
7.51
7.49
7.48
7.47
7.34
7.32
3.00
2.98
2.96
2.94
2.93
2.91
2.48
2.46
2.45
2.43
2.36
2.34
2.32
2.31
2.19
1.22
1.20
2.00
1.95
3.06
1.95
1.00
1.03
1.04
6.00
2.99
3r
^{1}H NMR (400 MHz, CDCl$_3$)

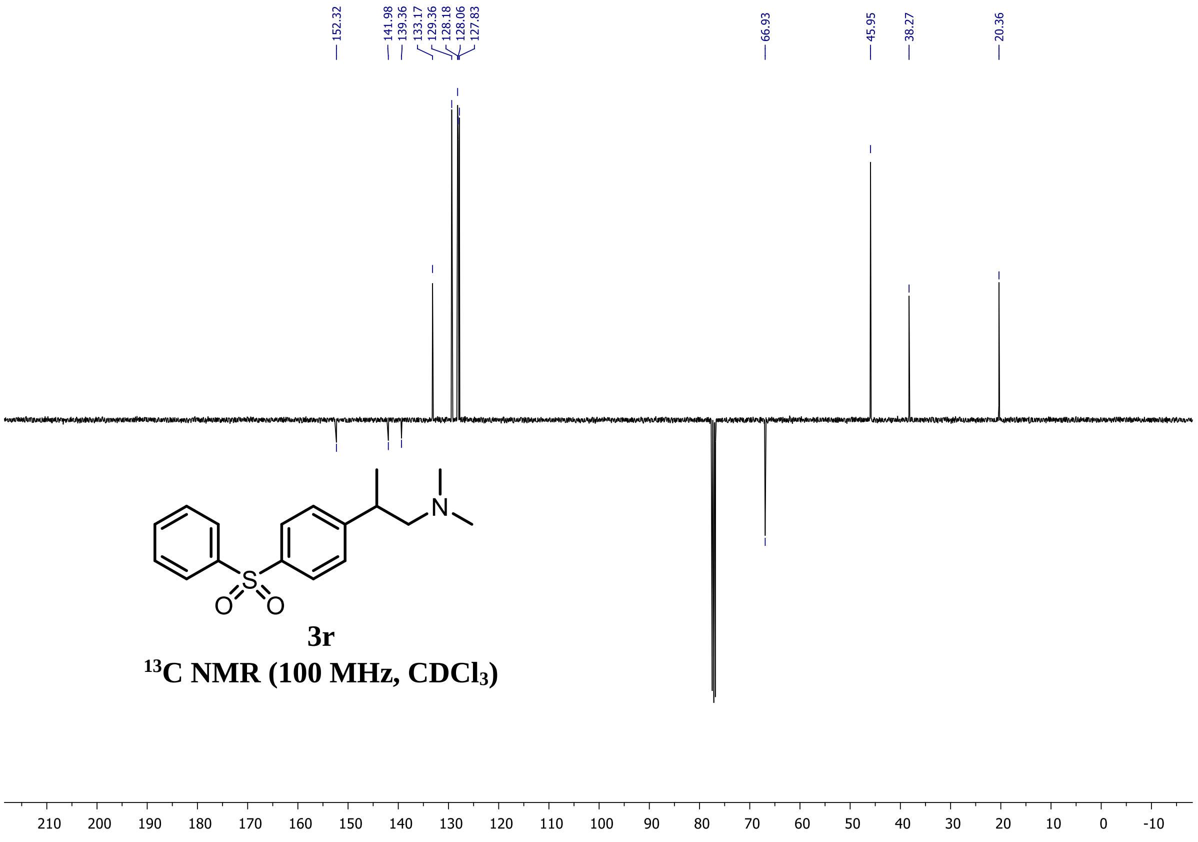
152.32
141.98
139.36
133.17
129.36
128.18
128.06
127.83
66.93
45.95
38.27
20.36
3r
^{13}C NMR (100 MHz, CDCl$_3$)

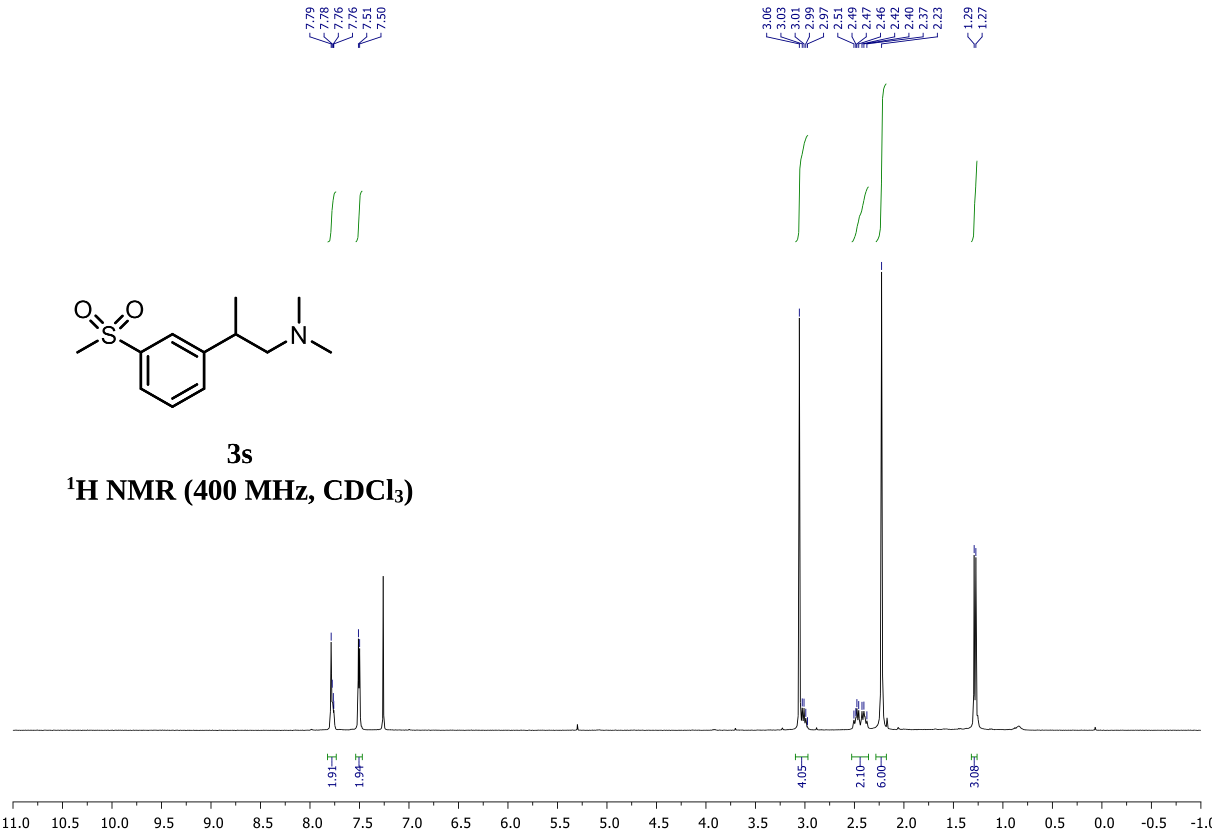

3s

^{1}H NMR (400 MHz, $CDCl_3$)

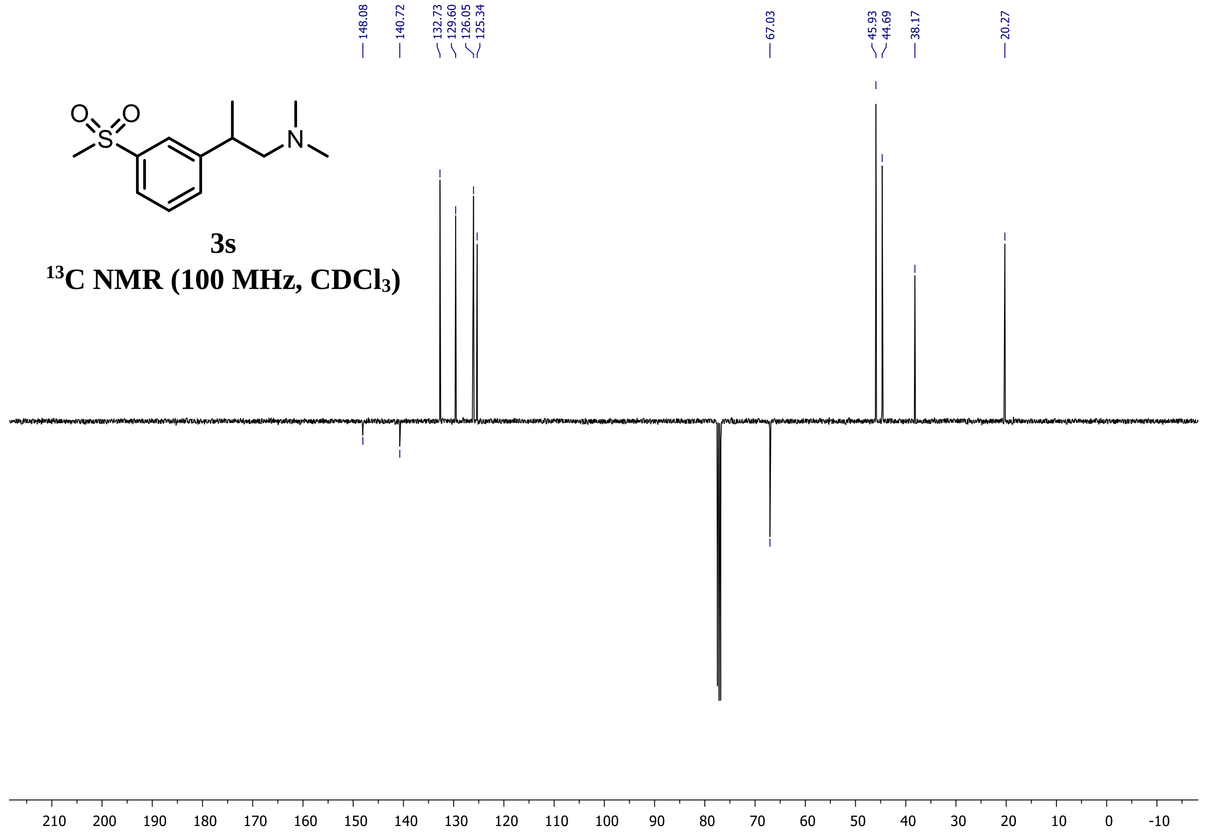

3s

^{13}C NMR (100 MHz, $CDCl_3$)

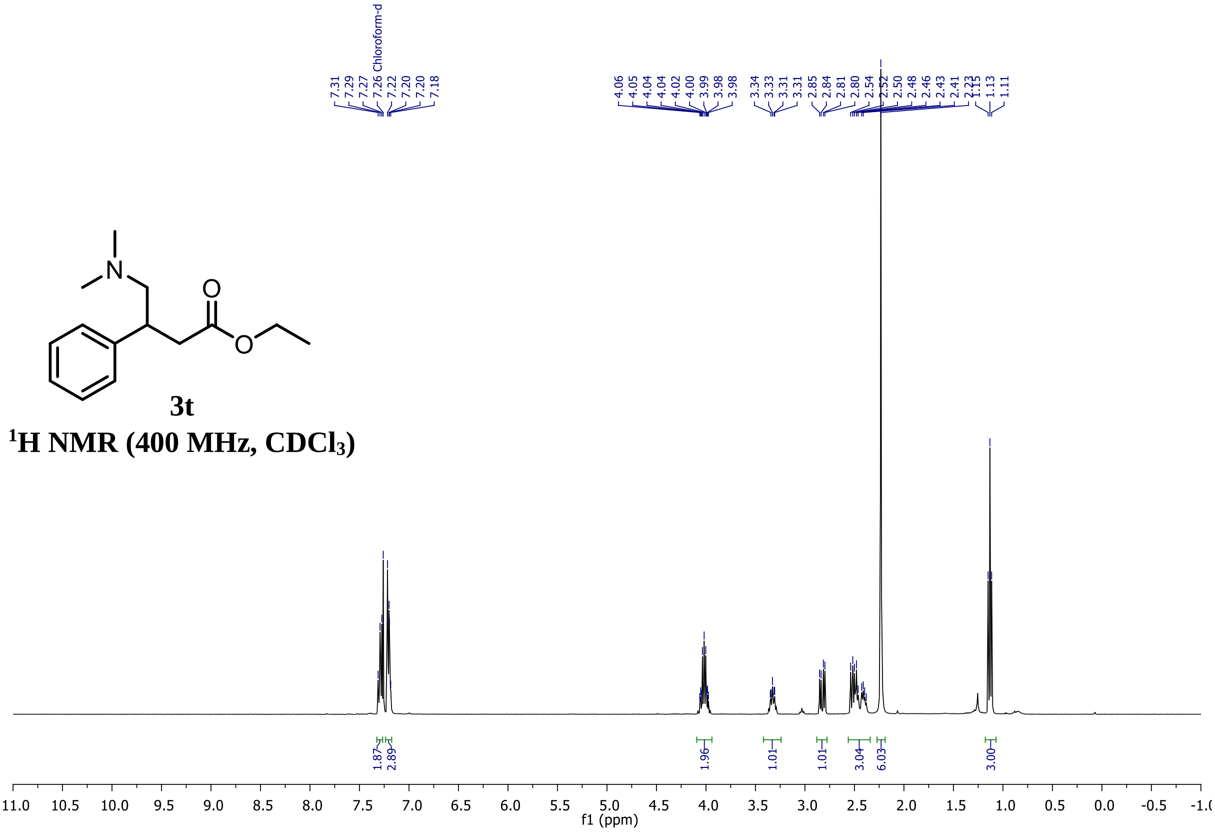
3t
^{1}H NMR (400 MHz, CDCl$_3$)
7.31 7.29 7.27 7.26 Chloroform-d 7.22 7.20 7.20 7.18
4.06 4.05 4.04 4.04 4.02 4.00 3.99 3.98 3.98
3.34 3.33 3.31 3.31 2.85 2.84 2.81 2.80 2.54 2.52 2.50 2.48 2.46 2.43 2.41 2.23 1.15 1.13 1.11
1.87 2.89 1.96 1.01 1.01 3.04 6.03 3.00
f1 (ppm)

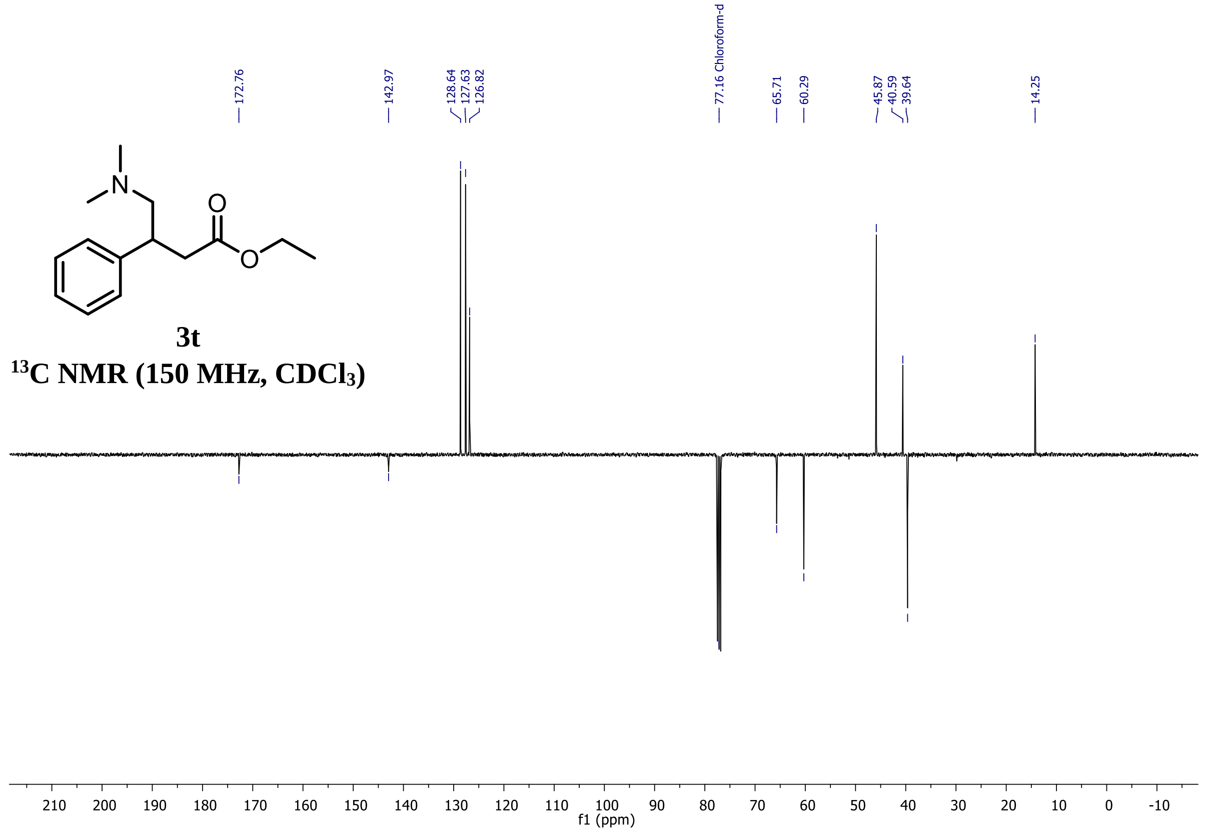
3t
^{13}C NMR (150 MHz, CDCl$_3$)
172.76 142.97 128.64 127.63 126.82 77.16 Chloroform-d 65.71 60.29 45.87 40.59 39.64 14.25
f1 (ppm)

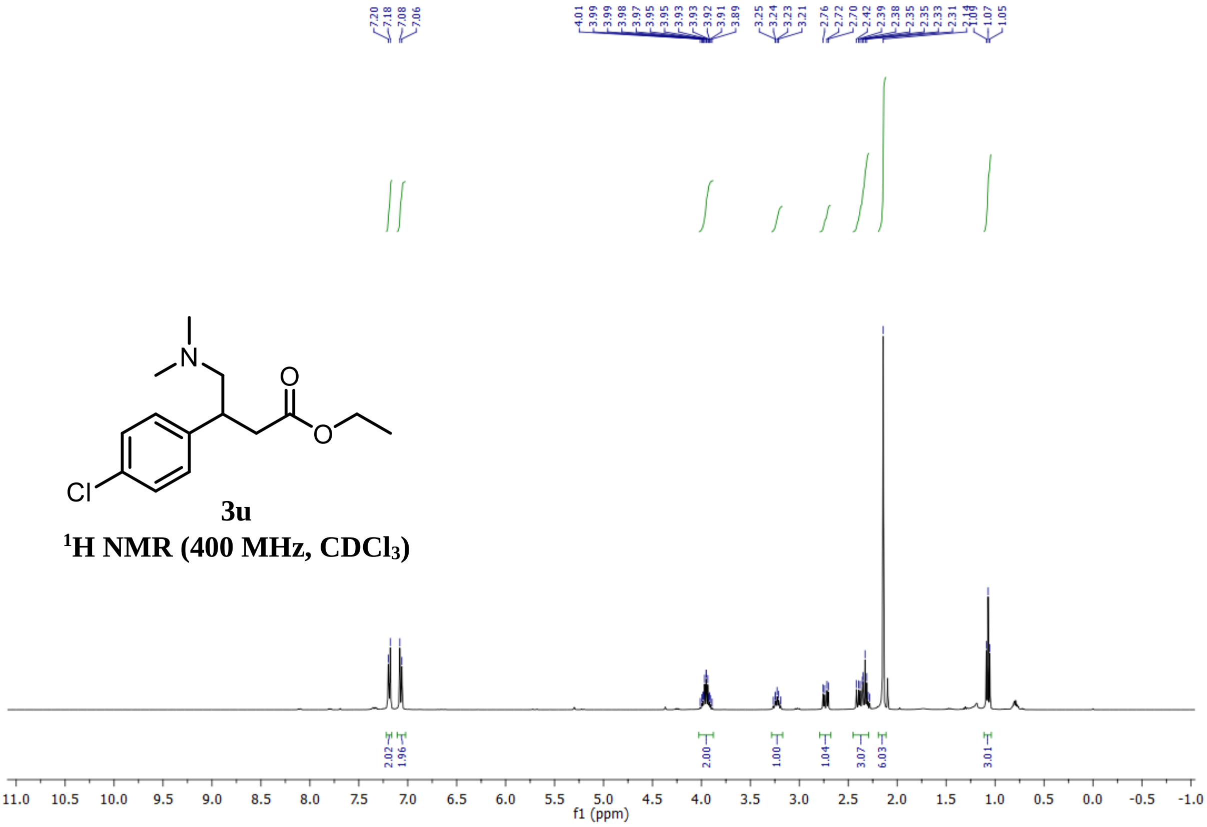
7.20
7.18
7.08
7.06
4.01
3.99
3.99
3.98
3.97
3.95
3.95
3.93
3.93
3.92
3.91
3.89
3.25
3.24
3.23
3.21
2.76
2.72
2.70
2.42
2.39
2.38
2.35
2.35
2.33
2.31
1.09
1.07
1.05
N
O
O
Cl
3u
1H NMR (400 MHz, CDCl3)
2.02
1.96
2.00
1.00
1.04
3.07
6.03
3.01
11.0
10.5
10.0
9.5
9.0
8.5
8.0
7.5
7.0
6.5
6.0
5.5
5.0
4.5
4.0
3.5
3.0
2.5
2.0
1.5
1.0
0.5
0.0
-0.5
-1.0
f1 (ppm)

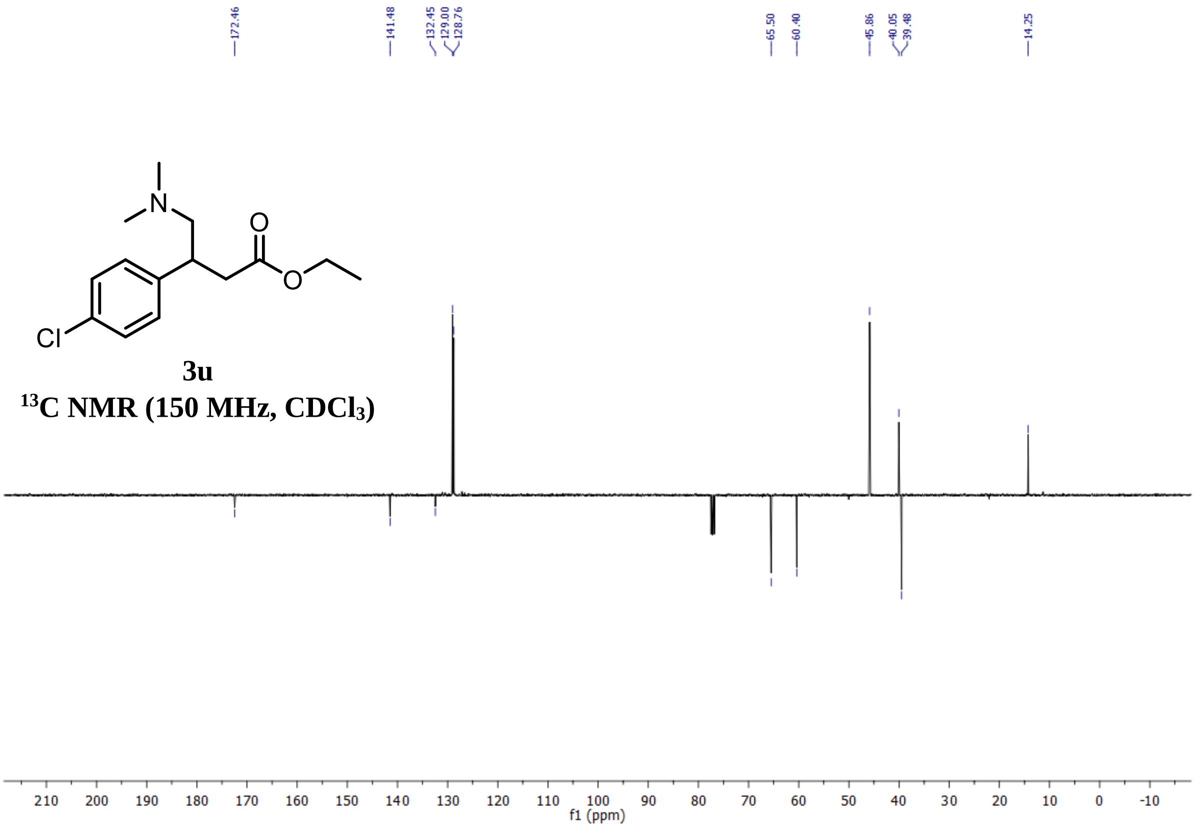
172.46
141.48
132.45
129.00
128.76
65.50
60.40
45.86
40.05
39.48
14.25
N
O
O
Cl
3u
13C NMR (150 MHz, CDCl3)
210
200
190
180
170
160
150
140
130
120
110
100
90
80
70
60
50
40
30
20
10
0
-10
f1 (ppm)

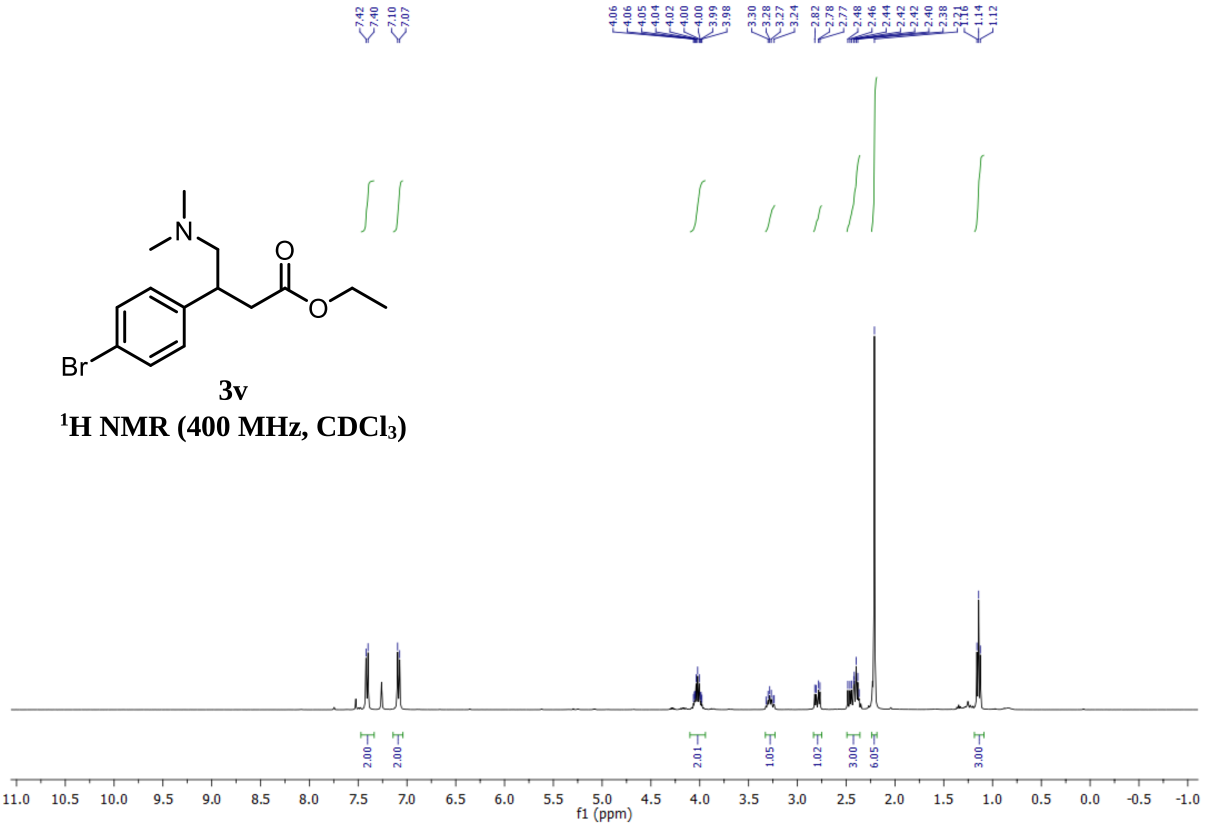
7.42
7.40
7.10
7.07
4.06
4.06
4.05
4.04
4.02
4.00
4.00
3.99
3.98
3.30
3.28
3.27
3.24
2.82
2.78
2.77
2.48
2.46
2.44
2.42
2.42
2.40
2.38
2.21
1.16
1.14
1.12
N
O
O
Br
3v
1H NMR (400 MHz, CDCl3)
2.00
2.00
2.01
1.05
1.02
3.00
6.05
3.00
f1 (ppm)

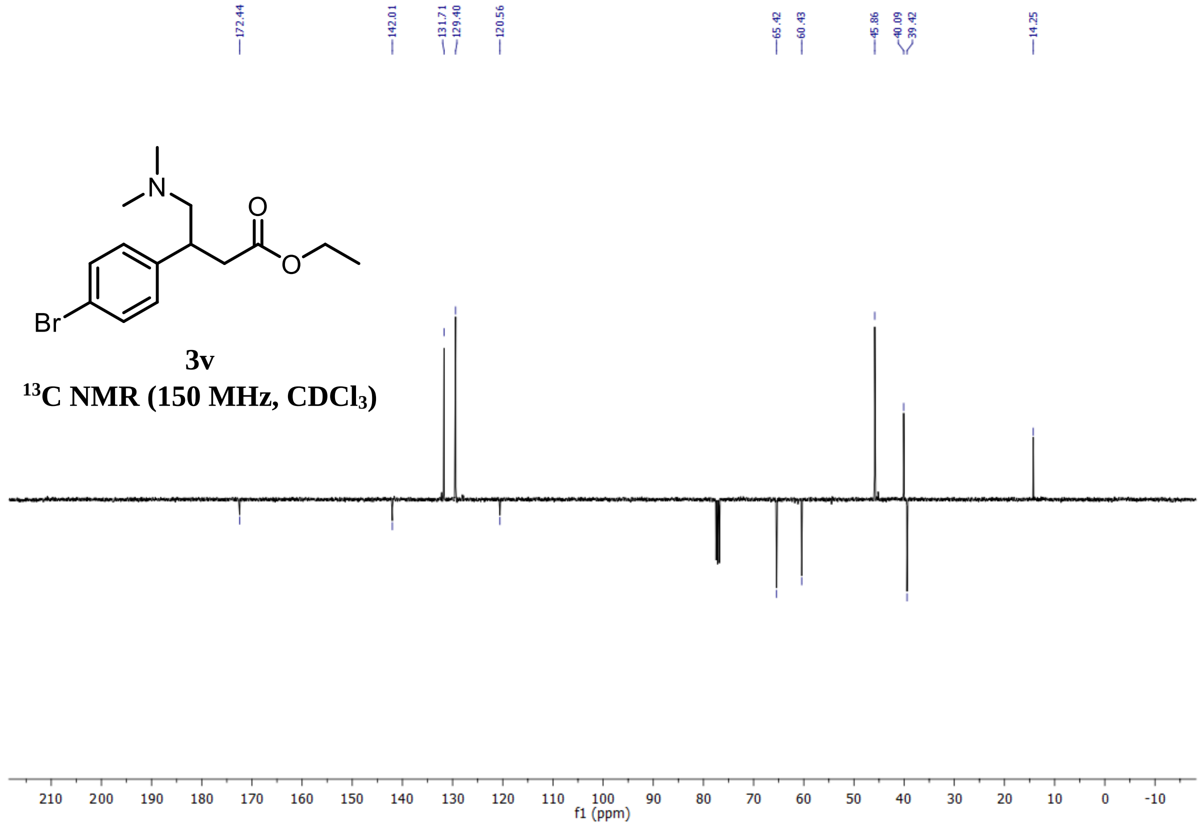
172.44
142.01
131.71
129.40
120.56
65.42
60.43
45.86
40.09
39.42
14.25
N
O
O
Br
3v
13C NMR (150 MHz, CDCl3)
f1 (ppm)

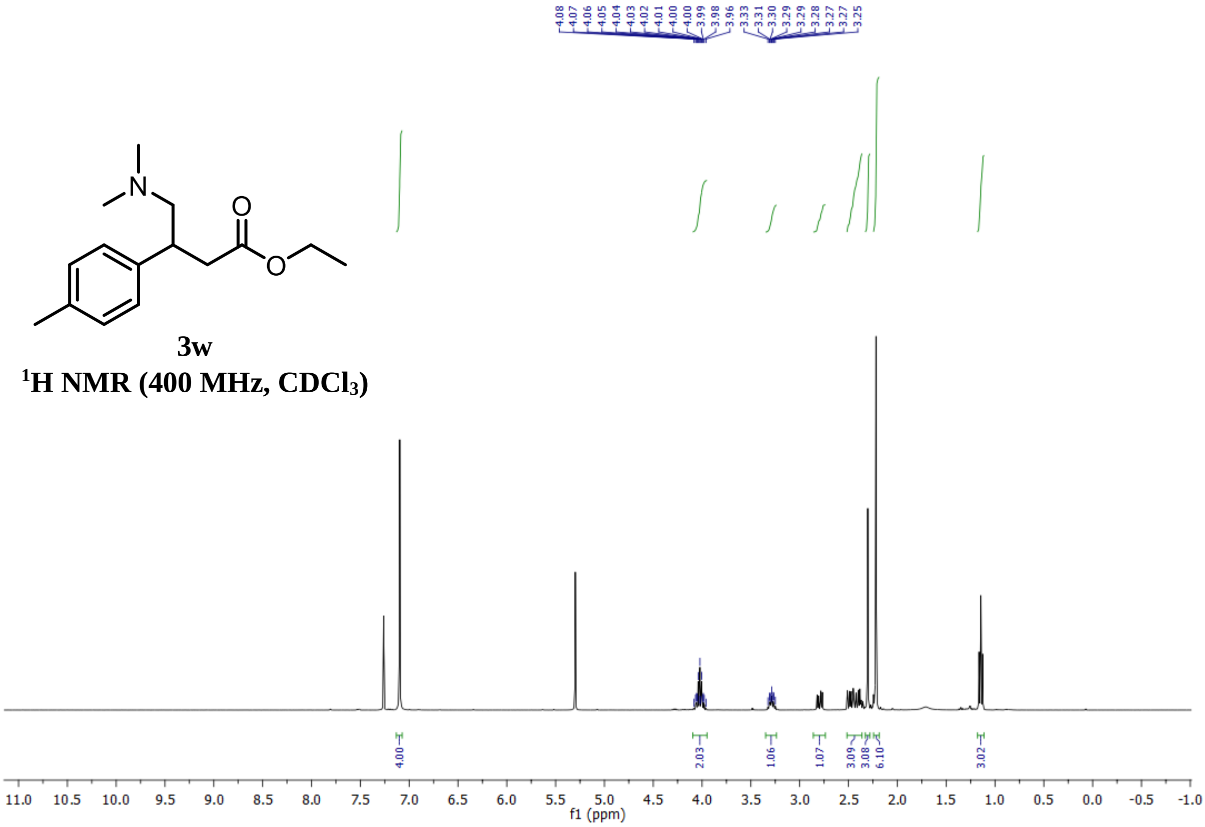
3w
^{1}H NMR (400 MHz, CDCl$_3$)
4.00
2.03
1.06
1.07
3.09
3.08
6.10
3.02
f1 (ppm)

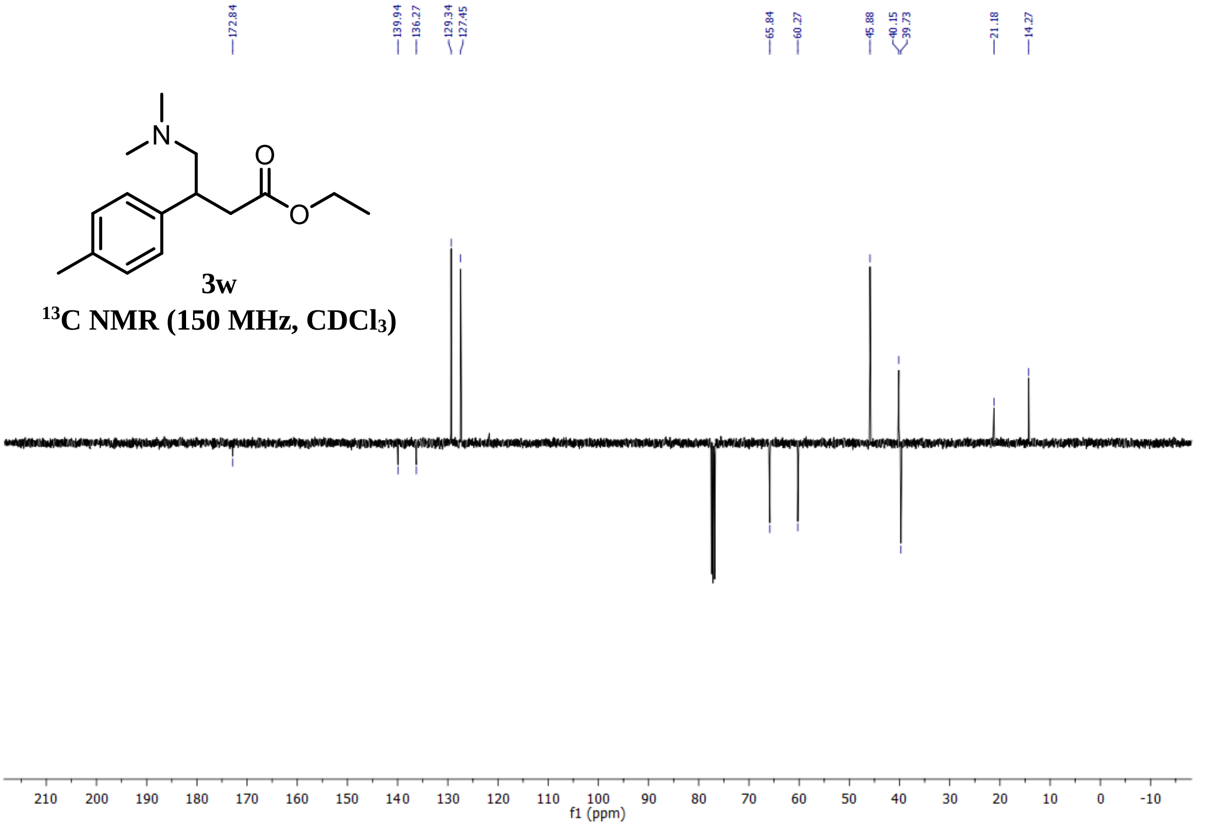
3w
^{13}C NMR (150 MHz, CDCl$_3$)
172.84
139.94
136.27
129.34
127.45
65.84
60.27
45.88
40.15
39.73
21.18
14.27
f1 (ppm)

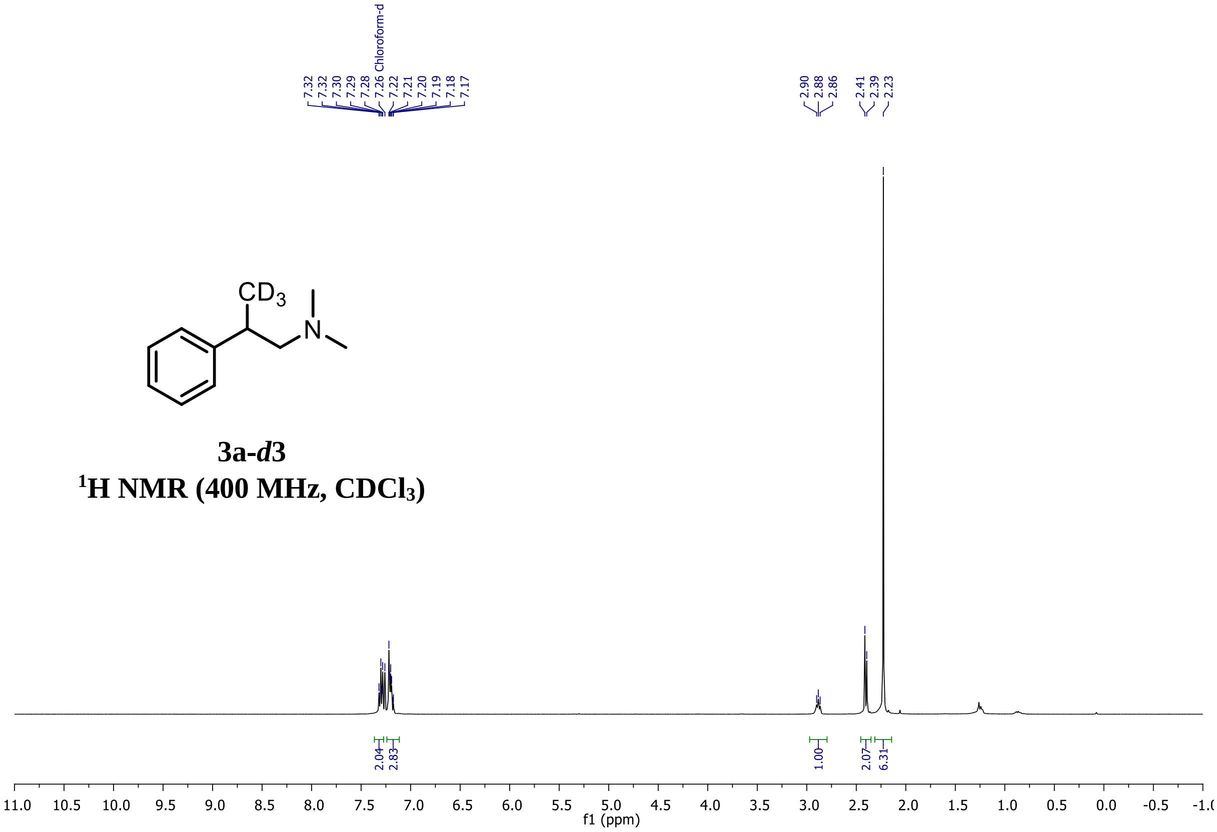
7.32 7.32 7.30 7.29 7.28 7.26 Chloroform-d 7.22 7.21 7.20 7.19 7.18 7.17
2.90 2.88 2.86 2.41 2.39 2.23
CD3
N
3a-d3
1H NMR (400 MHz, CDCl3)
2.04 2.83 1.00 2.07 6.31
11.0 10.5 10.0 9.5 9.0 8.5 8.0 7.5 7.0 6.5 6.0 5.5 5.0 4.5 4.0 3.5 3.0 2.5 2.0 1.5 1.0 0.5 0.0 -0.5
f1 (ppm)

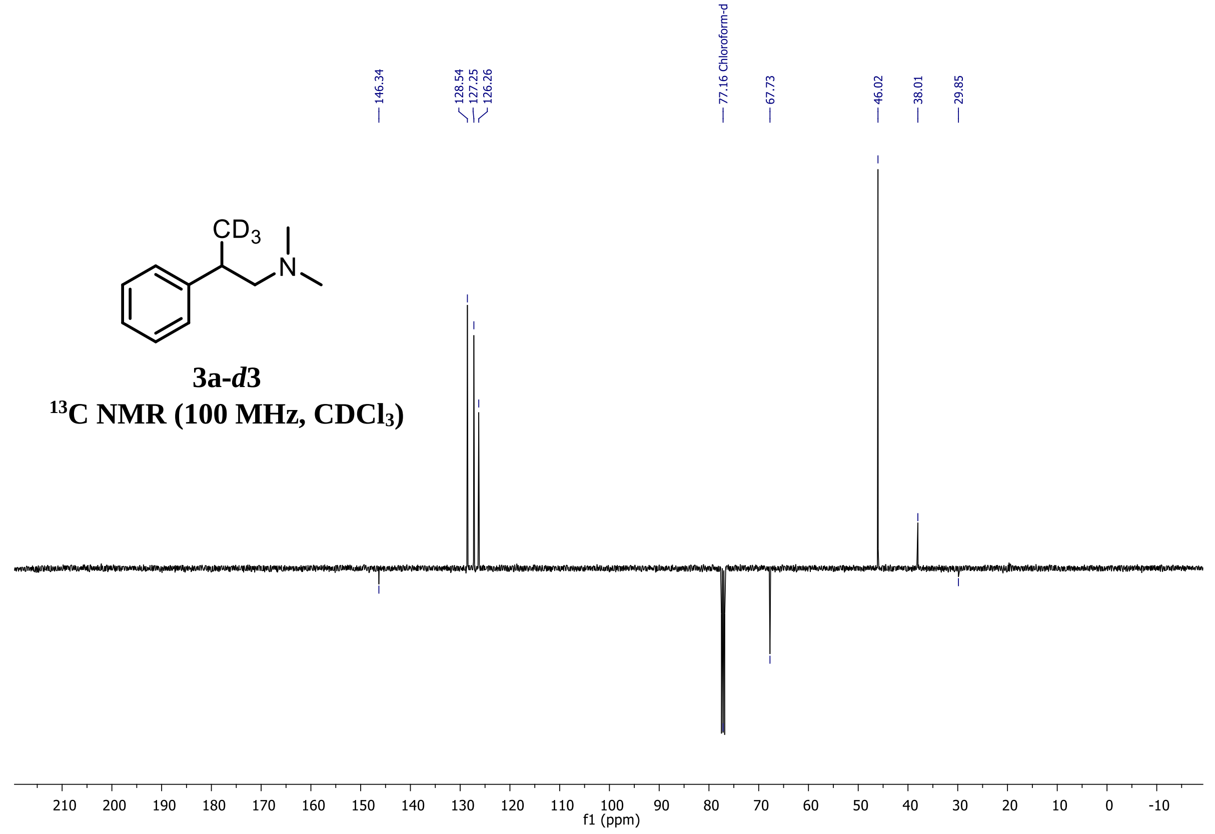
146.34
128.54 127.25 126.26
77.16 Chloroform-d
67.73
46.02
38.01
29.85
CD3
N
3a-d3
13C NMR (100 MHz, CDCl3)
210 200 190 180 170 160 150 140 130 120 110 100 90 80 70 60 50 40 30 20 10 0 -10
f1 (ppm)

Reaction of styrenes with disulfide and tetrabutylammonium iodide

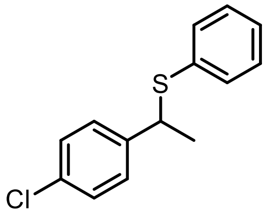

9a

^{1}H NMR (400 MHz, CDCl$_3$)

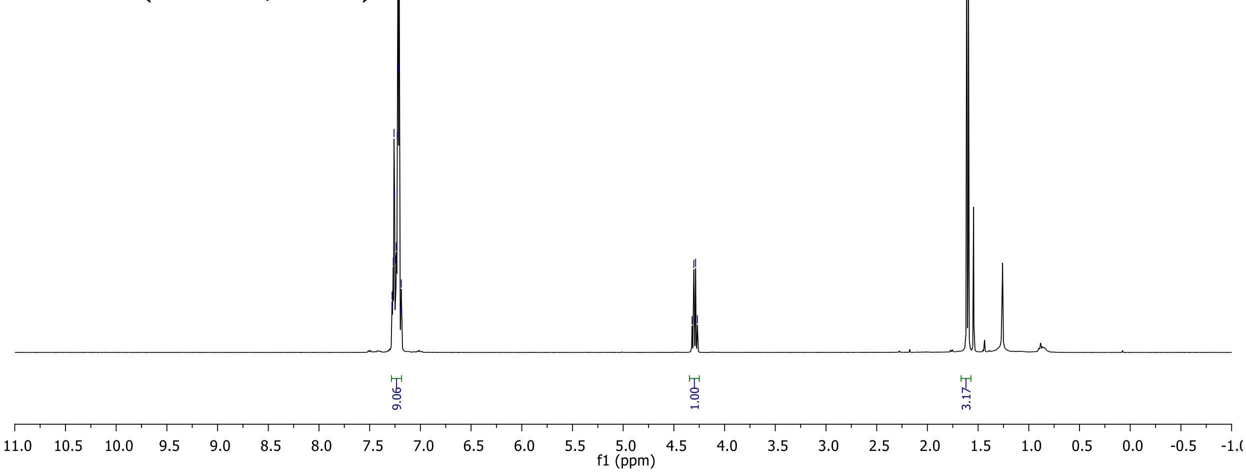

Reduction of styrenes by tetrabutylammonium iodide

10a

^{1}H NMR (400 MHz, CDCl$_3$)

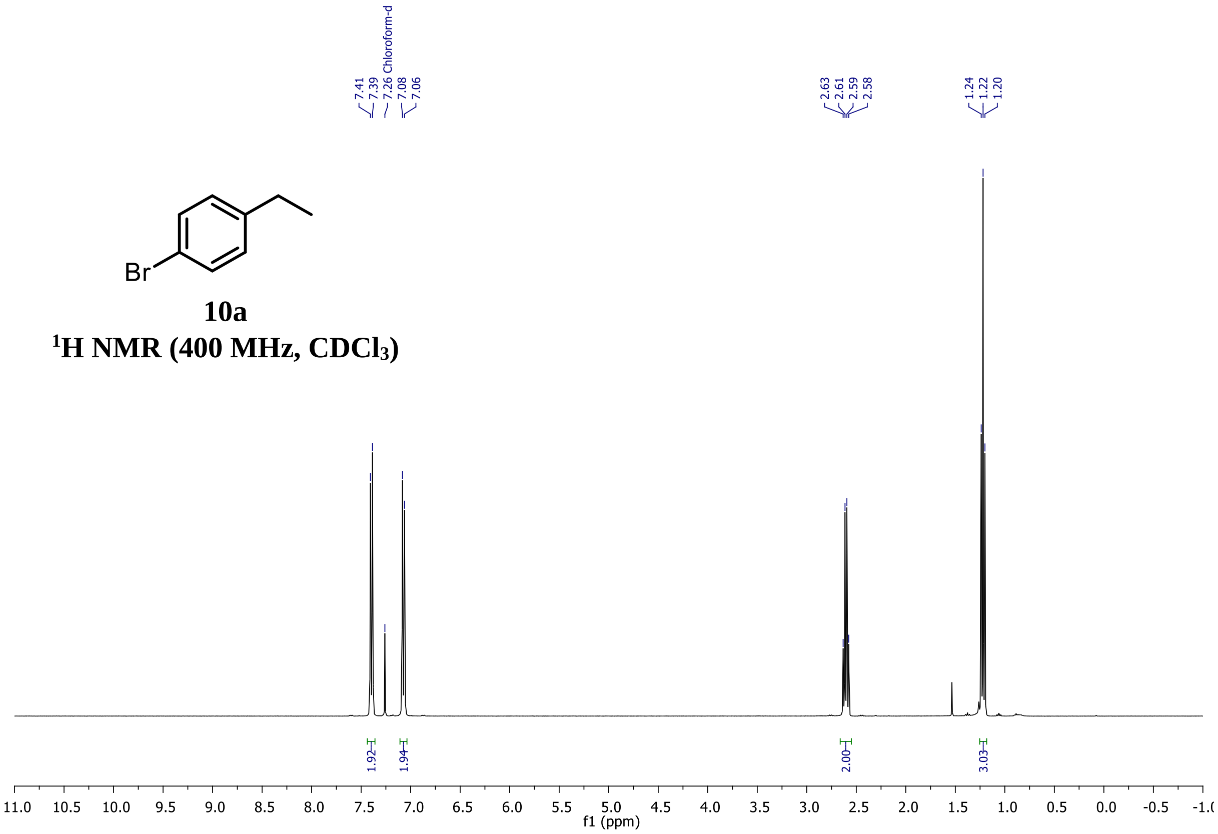

10b

^{1}H NMR (400 MHz, CDCl$_3$)

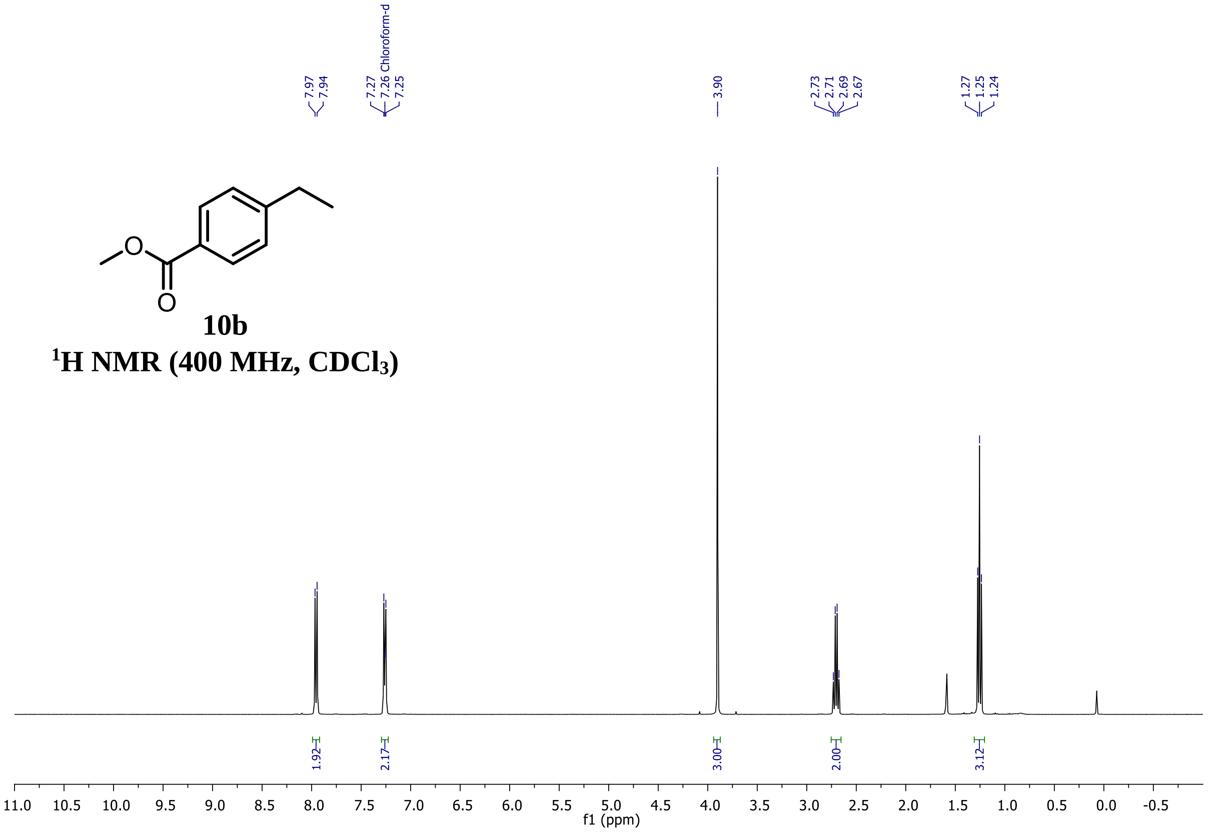

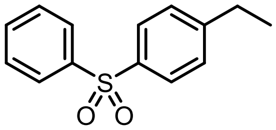

10c

^{1}H NMR (400 MHz, CDCl$_3$)

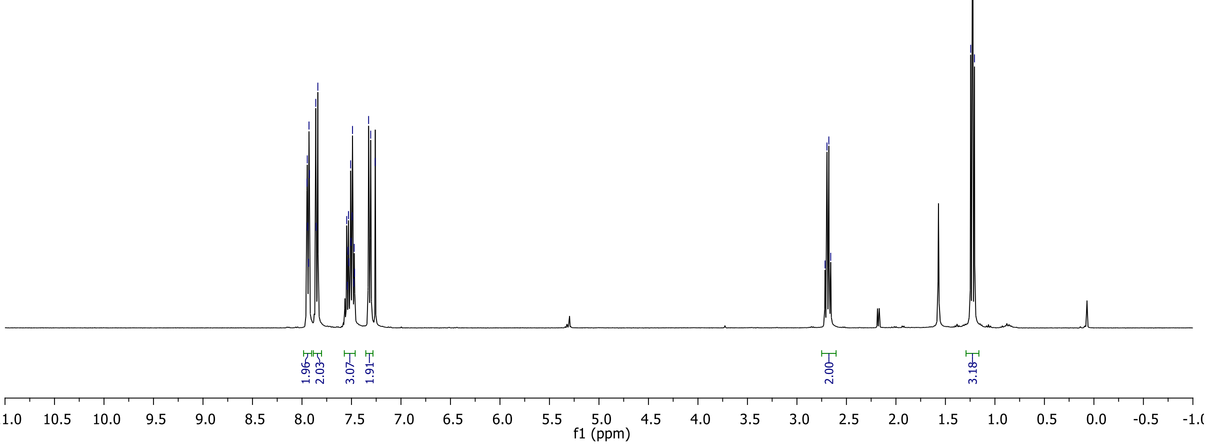

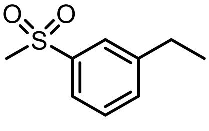

10d

^{1}H NMR (400 MHz, CDCl$_3$)

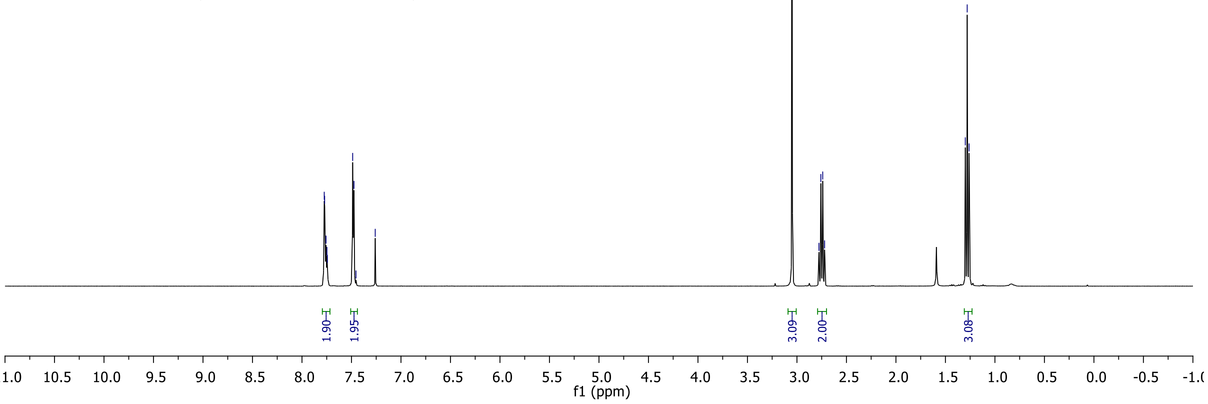

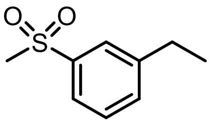

10d

^{13}C NMR (150 MHz, $CDCl_3$)

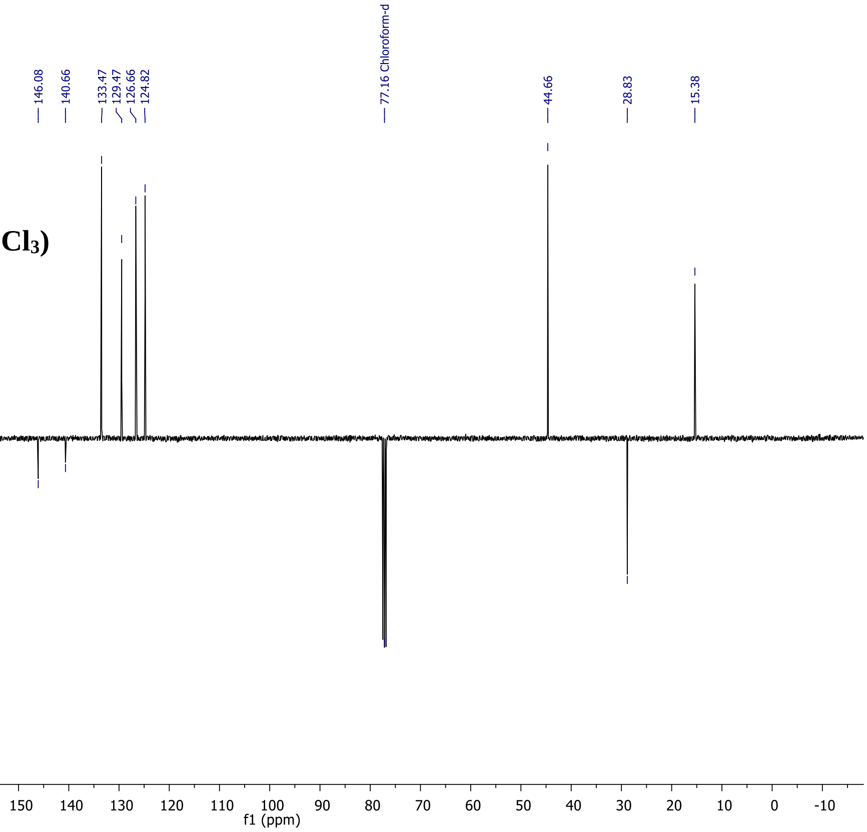

SI-1

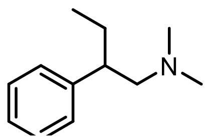

SI-1

^{1}H NMR (400 MHz, $CDCl_3$)

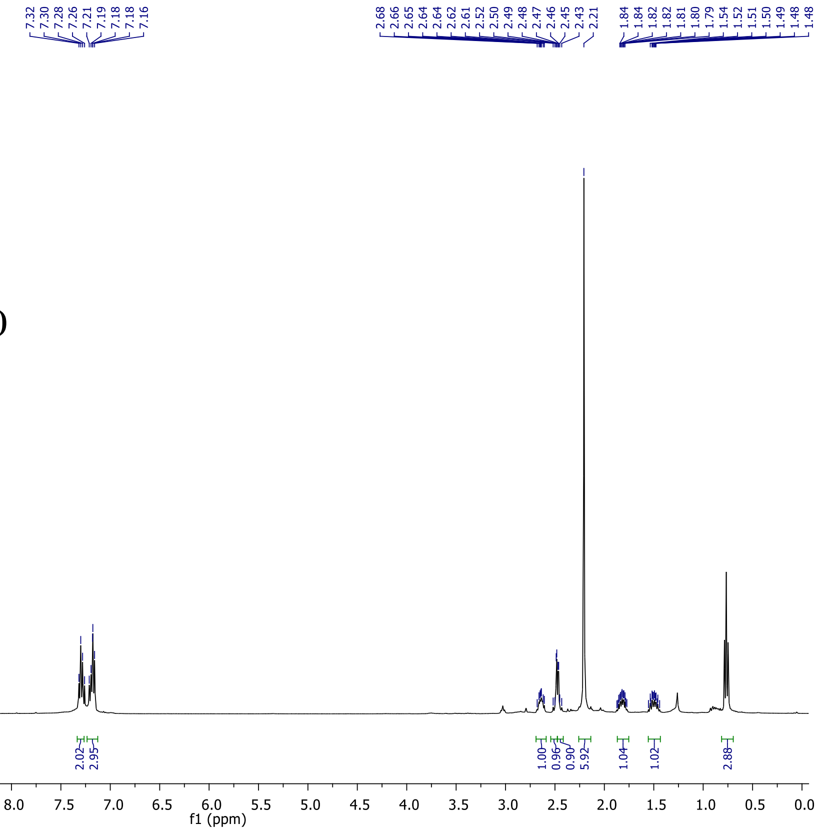

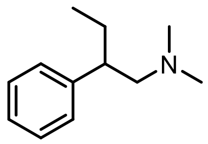

SI-1

^{13}C NMR (100 MHz, $CDCl_3$)

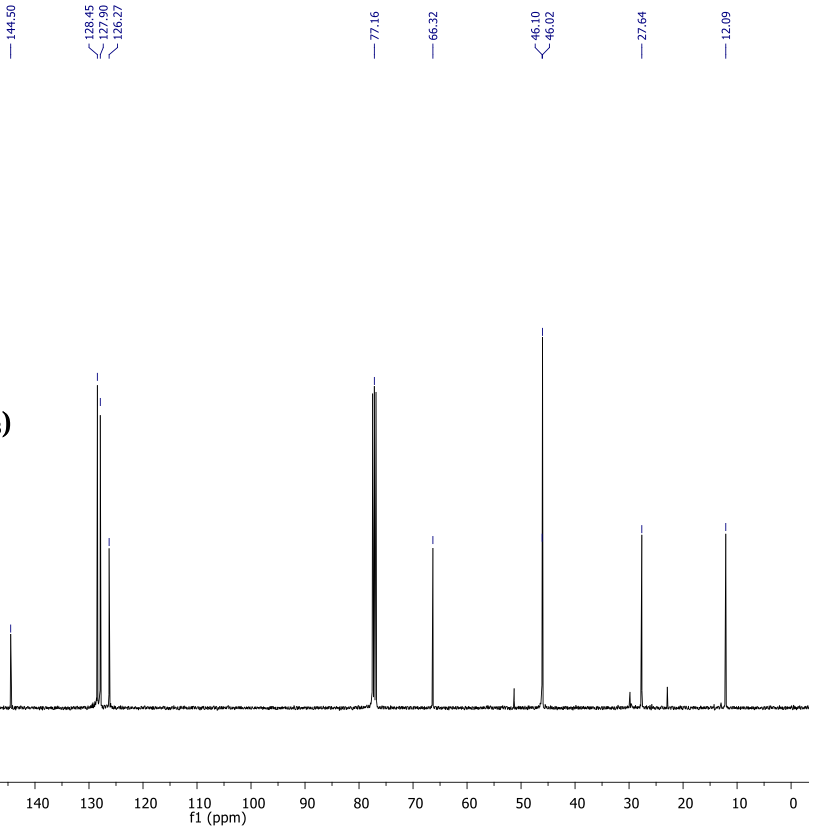

Computational details

The conformational space of all molecules has been searched using meta-dynamics simulations based on completely automated partially polarizable generic force-field (GFN-FF) calculations and, for comparative purposes, tight-binding quantum chemical calculations as implemented in CREST.[40–42]

The structures located with the CREST have then been subjected to M06-2X-D3/def2-TZVP[43–47] geometry optimization (*vide infra* for more details). The nature of all stationary points (minima and transition states) was verified through the computation of the vibrational frequencies at the level applied for the geometry optimization. Trifluoroacetic acid solvent was modeled using the polarizable continuum SMD[47,48] model with the refined parameters for iodine (SMD18)[49] and augmented by eps=8.42 and rsolv=2.479.[50] The DFT calculations have been performed with the Gaussian 16 program package.[51]

The Gibbs free energies are corrected at 348.15 K, with quasi-harmonic correction and concentration correction to 0.01 M performed using the GoodVibes program developed by the Paton group.[52]

Conformational sampling

The conformational sampling was performed as gas-phase calculations. Our technique to filter the most relevant conformations consists of several steps: 1) the CREST sampling (GFN-FF, gas-phase, NCI), 2) single-point quantum chemical (M06-2X-D3-SMD/def2-SVP) calculations for all found GFN-FF structures, 3) sorting the structures according to the M06-2X-D3-SMD/def2-SVP//GFN-FF energies, 4) optimization of the structures lying in ~30 kJ/mol energy window at the M06-2X-D3-SMD/def2-SVP, 5) re-optimization of the structures still remaining in the ~20 kJ/mol energy window at the M06-2X-D3-SMD/def2-TZVP.

Table S4 Energetic characteristics (total energies and free energies) for the intermediates A_t, B_t, C, D, and transition state TS_t. The single values of each conformer are denoted.

	Etot, a.u.	G_{298}, a.u[a]	*Grel, kcal/mol*
**A_t_1**	-1079.347946	-1079.144567	0.0
**A_t_2**	-1079.345757	-1079.143121	0.9
**A_t_3**	-1079.345747	-1079.142272	1.4
**A_t_4**	-1079.343426	-1079.141632	1.8
**A_t_5**	-1079.346372	-1079.141245	2.1
**A_t_6**	-1079.342800	-1079.139563	3.1
**TS_t_1**	-1079.342637	-1079.136174	0.0
**TS_t_2**	-1079.340582	-1079.134453	4.5
**B_t_1**	-1079.3617852	-1079.1531752	0.0

**B_t_2**	-1079.3628297	-1079.1508857	1.4
**B_t_3**	-1079.3444604	-1079.1395725	8.5
**B_t_4**	-1079.3447218	-1079.1385178	9.2
**B_t_5**	-1079.3427992	-1079.1369712	10.2
**C_1**	-1079.392323	-1079.182038	0.0
**C_2**	-1079.392323	-1079.181960	0.2
**C_3**	-1079.392519	-1079.181731	0.8
**C_4**	-1079.392859	-1079.181551	1.3
**C_5**	-1079.392547	-1079.181303	1.9
**C_6**	-1079.392321	-1079.180891	3.0
**C_7**	-1079.393014	-1079.180705	3.5
**C_8**	-1079.391930	-1079.180595	3.8
**C_9**	-1079.390833	-1079.180275	4.6
**C_10**	-1079.391529	-1079.180167	4.9
**C_11**	-1079.391839	-1079.180137	5.0
**C_12**	-1079.391957	-1079.179785	5.9
**C_13**	-1079.391321	-1079.179642	6.3
**C_14**	-1079.390026	-1079.177820	11.1
**C_15**	-1079.388996	-1079.177682	11.4
**C_16**	-1079.388999	-1079.177327	12.4
**C_17**	-1079.389788	-1079.176753	13.9
**D_1**	-1011.002927	-1010.748084	0.0
**D_2**	-1011.002928	-1010.748025	0.2
**D_3**	-1011.002384	-1010.747518	1.5
**D_4**	-1010.998499	-1010.746182	5.0
**D_5**	-1011.000347	-1010.745815	6.0
**D_6**	-1011.000347	-1010.745729	6.2
**D_7**	-1010.996750	-1010.745499	6.8
**D_8**	-1011.000771	-1010.745467	6.9
**D_9**	-1010.998470	-1010.744859	8.5
**D_10**	-1010.999063	-1010.744703	8.9

**D_11**	-1011.000105	-1010.744427	9.6
**D_12**	-1010.998934	-1010.743880	11.0
**D_13**	-1010.995748	-1010.742555	14.5
**D_14**	-1010.996181	-1010.742154	15.6
**D_15**	-1010.996681	-1010.740887	18.9

[a]These *G* values are taken directly from the Gaussian16 output files (M06-2X-D3-SMD/def2-TZVP) before additional corrections (temperature, concentration, quasi-harmonic correction) applied via GoodVibes software.

The influence of the DFT functional

Table S5 Two DFT functionals in comparison for the kinetic and thermodynamic properties of the $A_t \rightarrow B_t$ step. The A_t is used as the reference (0.0 kcal mol^{-1}).

Level of theory	Gibbs free energy ΔG_{348}, kcal mol^{-1}		
	**A_t**	**TS_t**	**B_t**
M06-2X-D3/def2-TZVP	0.0	4.8	-5.4
ωB97X-D/def2-TZVP	0.0	4.4	-7.1

The influence of the implicit solvation approach

Table S6 Alternative solvation approaches in comparison for the kinetic and thermodynamic properties of the $A_t \rightarrow B_t$ step as computed at the M06-2X-D3/def2-TZVP level of theory. The A_t is used as the reference (0.0 kcal mol^{-1}).

Solvation approach	Gibbs free energy ΔG_{348}, kcal mol^{-1}		
	**A_t**	**TS_t**	**B_t**
SMD18	0.0	4.8	-5.4
PCM	0.0	4.3	-5.0
PCM, surface=SAS	0.0	4.8	-3.3
Gas-phase	0.0	7.9	-4.6

Cartesian coordinates for the most stabilized (ΔG_{348}) conformations as computed at the M06-2X-D3/def2-TZVP level of theory

A'_1

C	-0.45683	-0.30395	0.00002
C	0.54272	-1.30648	0.00001
C	1.88255	-0.97873	-0.00000
C	2.28254	0.35796	-0.00002
C	1.31683	1.36144	-0.00000
C	-0.02879	1.04446	0.00002
C	-1.82640	-0.65656	-0.00001
H	0.23621	-2.34610	0.00004
H	2.62751	-1.76469	-0.00000
H	3.33419	0.61263	-0.00007
H	1.62111	2.40078	0.00000
H	-0.76590	1.83759	0.00005
C	-2.93133	0.33815	-0.00001
H	-2.07134	-1.71127	-0.00007
H	-2.88336	0.99313	0.87663
H	-2.88361	0.99283	-0.87690
H	-3.90245	-0.15266	0.00020

A'_2

I	-0.11618	1.53697	-0.03293
I	2.00757	-0.86949	0.01784
C	-1.68186	-1.31299	0.22160
H	-1.05582	-1.56931	-0.62217
N	-2.92645	-1.12322	0.04590
H	-1.26994	-1.26997	1.22086
C	-3.53819	-1.15000	-1.28154
C	-3.81879	-0.74748	1.14023
H	-3.27367	-0.77462	2.07811
H	-4.18991	0.25839	0.94660
H	-4.65567	-1.44279	1.16100
H	-4.37897	-1.84059	-1.26202
H	-3.89925	-0.14823	-1.51119
H	-2.80197	-1.46401	-2.01457

At

I	-1.74562	-1.71671	0.43824
I	-2.49807	1.32762	-0.25317
C	3.13026	-0.94023	-0.67022
C	4.09313	0.08437	-0.50598
C	4.38931	0.59102	0.74232
C	3.74966	0.09162	1.87894
C	2.81934	-0.93581	1.74465
C	2.50989	-1.44828	0.49670
C	2.77475	-1.39146	-1.96216
H	4.58825	0.47808	-1.38646
H	5.12439	1.38065	0.84126
H	3.98362	0.49201	2.85672
H	2.32499	-1.33512	2.62184
H	1.77071	-2.23513	0.40977
C	1.73961	-2.42866	-2.21039
C	0.74703	0.92718	0.15642
H	3.30183	-0.96174	-2.80522
H	2.03499	-3.39674	-1.78970
H	0.78571	-2.16984	-1.73632
H	1.56498	-2.56565	-3.27548
H	0.52068	0.26156	-0.66587
N	1.41117	1.99021	-0.05621
H	0.41806	0.68917	1.15878
C	1.85737	2.37065	-1.39481
C	1.73575	2.93475	1.01060
H	1.40767	2.53249	1.96434
H	2.81170	3.09766	1.01521
H	1.22849	3.87637	0.80092
H	1.66384	1.55630	-2.08796
H	1.30976	3.26351	-1.69685
H	2.92132	2.59544	-1.35679

As

I	-1.75211	-1.71640	0.43796
I	-2.49790	1.32892	-0.25437
C	3.13678	-0.94427	-0.66348
C	4.09444	0.08740	-0.51399
C	4.39304	0.60865	0.72772
C	3.76091	0.11766	1.87214
C	2.83561	-0.91612	1.75246
C	2.52392	-1.44338	0.51127
C	2.77961	-1.41187	-1.94918
H	4.58375	0.47460	-1.40059
H	5.12416	1.40328	0.81532
H	3.99672	0.52952	2.84469
H	2.34727	-1.30899	2.63589
H	1.78937	-2.23572	0.43576
C	1.75149	-2.45945	-2.18252
C	0.74677	0.92072	0.15180
H	3.30143	-0.98844	-2.79866
H	2.05796	-3.42168	-1.75627
H	0.79852	-2.20500	-1.70445
H	1.57131	-2.60642	-3.24535
H	0.52354	0.25170	-0.66866
N	1.40682	1.98570	-0.06362
H	0.41840	0.68429	1.15474
C	1.85156	2.36444	-1.40317
C	1.72589	2.93510	1.00056
H	1.40133	2.53296	1.95556
H	2.80069	3.10544	1.00386
H	1.21190	3.87265	0.78892
H	1.66212	1.54717	-2.09405
H	1.29968	3.25377	-1.70791

H	2.91438	2.59445	-1.36555

TSt

I	-2.86490	-1.32718	-0.24532
I	-1.98260	1.70270	0.37370
C	3.26456	0.71814	-0.23842
C	3.55725	0.58935	1.13093
C	4.77462	0.08433	1.55155
C	5.72225	-0.31539	0.61460
C	5.44934	-0.19124	-0.74489
C	4.23700	0.32145	-1.17146
C	1.95795	1.18220	-0.62926
H	2.81355	0.90060	1.85573
H	4.98745	-0.00170	2.60934
H	6.67268	-0.71661	0.94161
H	6.18904	-0.49706	-1.47350
H	4.03635	0.40868	-2.23164
C	1.64197	1.73839	-1.97285
C	0.88861	-0.71426	-0.82725
H	1.32730	1.52738	0.18574
H	0.56265	1.78874	-2.12298
H	2.02929	2.76131	-2.03777
H	2.08659	1.16540	-2.78620
H	1.29643	-0.88842	-1.81528
N	1.22402	-1.54855	0.11654
H	-0.03001	-0.15183	-0.71237
C	2.34740	-2.46073	-0.03094
C	0.65159	-1.45080	1.45049
H	0.18467	-2.40031	1.71244
H	1.45354	-1.24325	2.16120
H	-0.08952	-0.65546	1.47575
H	3.19129	-2.09609	0.55915
H	2.05361	-3.44180	0.34204
H	2.62883	-2.53076	-1.07788

TSs

I	-2.86636	-1.32442	-0.24332
I	-1.97731	1.70376	0.37344
C	3.26012	0.71637	-0.23900
C	3.55084	0.58906	1.13088
C	4.76901	0.08790	1.55378
C	5.71938	-0.30944	0.61859
C	5.44841	-0.18674	-0.74141
C	4.23528	0.32219	-1.17024
C	1.95276	1.17640	-0.63231
H	2.80494	0.89838	1.85427
H	4.98034	0.00300	2.61197
H	6.67046	-0.70766	0.94738
H	6.19024	-0.49069	-1.46862
H	4.03612	0.40832	-2.23080
C	1.63828	1.73202	-1.97654
C	0.88789	-0.72178	-0.83151
H	1.31943	1.51975	0.18138
H	0.55914	1.78235	-2.12785
H	2.02586	2.75482	-2.04154
H	2.08385	1.15864	-2.78911
H	1.29745	-0.89519	-1.81893
N	1.22434	-1.55496	0.11306
H	-0.03261	-0.16236	-0.71830
C	2.35064	-2.46380	-0.03273
C	0.65056	-1.45781	1.44651
H	0.18453	-2.40780	1.70826
H	1.45171	-1.24918	2.15780
H	-0.09155	-0.66337	1.47103
H	3.19302	-2.09610	0.55761
H	2.05950	-3.44537	0.34102
H	2.63295	-2.53407	-1.07941

Bt

C	2.69786	-0.24772	0.27614
C	4.04207	0.08289	0.44340
C	4.99803	-0.90994	0.60161
C	4.62127	-2.24793	0.59675
C	3.28523	-2.58539	0.43212
C	2.32958	-1.58986	0.26963
H	1.28504	-1.85261	0.14148
H	2.98291	-3.62487	0.43023
H	5.36697	-3.02252	0.72191
H	6.03848	-0.64001	0.73078
H	4.34533	1.12397	0.45177
C	1.64671	0.82410	0.10941
C	1.61916	1.81051	1.26973
H	2.54601	2.38555	1.32714
H	1.49407	1.26674	2.20619
H	0.78101	2.50456	1.18213
H	0.67285	0.33470	0.03401
C	1.90582	1.53690	-1.25138
N	0.91257	2.54776	-1.49012
C	-0.42899	2.14913	-1.80206
H	-0.94516	2.96252	-2.30845
H	-0.42056	1.23821	-2.39556
H	-0.95010	1.93867	-0.85311
C	1.15099	3.92710	-1.15868
H	2.11521	4.03874	-0.67358
H	0.34031	4.28488	-0.52004
H	1.12718	4.51289	-2.08384
H	1.83553	0.79709	-2.04986
H	2.88693	2.00879	-1.25088
I	-1.72691	-1.46464	-1.12142
I	-2.14283	0.43463	1.43501

C

I	-1.09707	-0.70028	0.36447
I	-3.21866	0.78807	-0.27498

C	2.64182	-1.13417	-0.04102
C	2.06605	-1.60723	1.13938
C	1.31114	-2.77486	1.15319
C	1.11770	-3.49231	-0.02407
C	1.68483	-3.03153	-1.20257
C	2.43837	-1.86237	-1.21017
C	3.40523	0.17402	-0.02410
H	2.21644	-1.05358	2.06025
H	0.88173	-3.12827	2.08263
H	0.53019	-4.40128	-0.01736
H	1.54074	-3.58147	-2.12400
H	2.87064	-1.52004	-2.14196
C	4.45530	0.28341	-1.12191
C	2.37414	1.30674	-0.10023
H	3.90691	0.23022	0.94576
H	3.99345	0.40799	-2.10337
H	5.09639	1.14728	-0.94945
H	5.08458	-0.60768	-1.14964
H	1.68816	1.21015	0.75985
N	2.93149	2.65482	-0.14122
H	1.77275	1.16339	-1.00341
C	3.71624	2.92259	1.05481
C	1.82871	3.60339	-0.21116
H	1.22545	3.40965	-1.09849
H	1.17236	3.53471	0.67090
H	2.21439	4.62109	-0.27094
H	3.13915	2.73404	1.97467
H	4.61323	2.30467	1.07830
H	4.02914	3.96658	1.05878

D

C	-0.10030	-2.42167	1.42936
C	-0.93713	-2.59608	0.33599
C	-0.54034	-2.13401	-0.91332
C	0.68002	-1.49172	-1.06318
C	1.52409	-1.30284	0.02981
C	1.12205	-1.78044	1.27428
H	1.77134	-1.64179	2.13177
C	2.84251	-0.57581	-0.13173
C	3.76338	-1.27352	-1.13337
H	3.31182	-1.29698	-2.12656
H	4.71993	-0.75355	-1.20551
H	3.95158	-2.30037	-0.82095
C	2.63390	0.85989	-0.60304
N	1.74980	1.67155	0.28109
H	0.80283	1.22780	0.35762
C	1.53043	3.01702	-0.30088
H	1.17526	2.90546	-1.32158
H	0.78186	3.53166	0.29702
H	2.47207	3.56316	-0.28082
C	2.25169	1.78086	1.67056
H	1.60560	2.46865	2.21061
H	2.21475	0.80597	2.14842
H	3.27236	2.15952	1.64783
H	3.58678	1.38874	-0.66471
H	2.16683	0.86700	-1.58893
H	3.34564	-0.56288	0.83705
H	0.96927	-1.12155	-2.04031
H	-1.18810	-2.26277	-1.77110
H	-1.89537	-3.08563	0.45547
H	-0.40167	-2.77718	2.40650
O	-1.11452	1.27877	-1.30761
C	-1.45937	0.91242	-0.17877
O	-0.81866	0.92803	0.89037
C	-2.92484	0.40564	-0.07423
F	-3.77046	1.44790	-0.12955
F	-3.17808	-0.24434	1.06150
F	-3.24730	-0.41122	-1.08201

I_rad_anion

I	0.00000	0.00000	1.60800
I	0.00000	0.00000	-1.60800

References

[26] S. Bezerra França, L. C. Barros de Lima, C. R. da Silva Cunha, D. Santos Anunciação, E. Ferreira da Silva-Júnior, M. E. de Sá Barreto Barros, D. J. da Paz Lima, *Bioorg. Med. Chem.* **2021**, *44*, 116299.

[27] F. Scheidt, J. Neufeld, M. Schäfer, C. Thiehoff, R. Gilmour, *Org. Lett*. **2018**, *20*, 8073–8076.

[28] Y.-M. Wang, J. Wu, C. Hoong, V. Rauniyar, F. D. Toste, *J. Am. Chem. Soc.* **2012**, *134*, 12928–12931.

[29] J. Holz, C. Pfeffer, H. Zuo, D. Beierlein, G. Richter, E. Klemm, R. Peters, *Angew. Chem. Int. Ed.* **2019**, *58*, 10330–10334.

[30] K. Polidano, B. G: Reed-Berendt, A. Basset, A. J. A. Watson, J. M. J. Williams, L. C. Morrill, *Org. Lett.* **2017**, *19*, 6716–6719.

[31] B. Das, Y. Srinivas, H. Holla, R. Narender, *Chem. Lett.* 2007, *36*, 800–801.

[32] L. Yang, J. Lin, L. Kang, W. Zhou, D.-Y. Ma, *Adv. Synth. Catal.* **2018**, *360*, 485–490.

[33] S. Zhu, S. L. Buchwald, *J. Am. Chem. Soc.* **2014**, *136*, 15913–15916.

[34] J. A. Fuentes, P. Wawrzyniak, G. J. Roff, M. Bühl, M. L. Clarke, *Catal. Sci. Technol.* **2011**, *1*, 431–436.

[35] S. Santra, J. Guin, *Eur. J. Org. Chem.* **2015**, *2015*, 7253–7257.

[36] P. J. Rushworth, D. G: Hulcoop, D. J. Fox, *J. Org. Chem.* **2013**, *78*, 9517–9521.

[37] B. Gong, H. Zhu, L. Yang, H. Wang, Q. Fan, Z. Xie, Z. Le, *Org. Biomol. Chem.* **2022**, *20*, 3501–3505.

[38] J. R. Cabrero-Antonino, A. Leyva-Pérez, A. Corma, *Adv. Synth. Catal.* **2012**, *354*, 678–687.

[39] Q. Zhu, E. C. Gentry, R. R: Knowles, *Angew. Chem. Int. Ed.* **2016**, *55*, 9969–9973.

[40] P. Pracht, F. Bohle, S. Grimme, *Phys. Chem. Chem. Phys.* **2020**, *22*, 7169–7192.

[41] S. Grimme, *J. Chem. Theory Comput.* **2019**, *15*, 2847–2862.

[42] S. Spicher, S. Grimme, *Angew. Chem. Int. Ed.* **2020**, *59*, 15665–15673.

[43] Y. Zhao, D. G. Truhlar, *Theor. Chem. Acc.* **2008**, *120*, 215–241.

[44] S. Grimme, J. Antony, S. Ehrlich, H. Krieg, *J. Chem. Phys*. **2010**, *132*, 154104.

[45] F. Weigend, R. Ahlrichs, *Phys. Chem. Chem. Phys.* **2005**, *7*, 3297–3305.

[46] F. Weigend, *Phys. Chem. Chem. Phys*. **2006**, *8*, 1057.

[47] E. Cancès, B. Mennucci, J. Tomasi, *J. Chem. Phys*. **1997**, *107*, 3032–3041.

[48] A. V: Marenich, C. J. Cramer, D. G: Truhlar, *J. Phys. Chem. B* **2009**, *113*, 6378–6396.

[49] E. Engelade, N. Schulz, F. Heinen, S. M. Huber, D. G: Truhlar, *Chem. Eur. J.* **2018**, *24*, 15983–15987.

[50] S. M. Bischof, D. H. Ess, S. K. Meier, J. Oxgaard, R. J. Nielsen, G: Bhalla, W. A. Goddard, R. A. Periana, *Organometallics* **2010**, *29*, 742–756.

[51] M. J. Frisch, G. W. Trucks, H. B. Schlegel, G. E. Scuseria, M. A. Robb, J. R. Cheeseman, G. Scalmani, V. Barone, B. Mennucci, G. A. Petersson, H. Nakatsuji, M. Caricato, X. Li, H. Hratchian, A. Izmaylov, J. Bloino, G. Zheng, J. Sonnenberg, M. Hada, M. Ehara, K. Toyota, R. Fukuda, J. Hasegawa, M. Ishida, T. Nakajima, Y. Honda, O. Kitao, H. Nakai, T. Vreven, J. Montgomery, J. Peralta, F. Ogliaro, M. Bearpark, J. Heyd, E. Brothers, K. Kudin, V. Staroverov, R. Kobayashi, J. Normand, K. Raghavachari, A. Rendell, J. Burant, S. Iyengar, J. Tomasi, M. Cossi, N. Rega, J. Millam, M. Klene, J. Knox, J. Cross, V. Bakken, C. Adamo, J. Jaramillo, R. Gomperts, R. Stratmann, O. Yazyev, A. Austin, R. Cammi, C. Pomelli, J. Ochterski, R. Martin, K. Morokuma, V. Zakrzewski, G. Voth, P. Salvador, J. Dannenberg, S. Dapprich, A. Daniels, O. Farkas, J. Foresman, J. Ortiz, J. Cioslowski, D. Fox, Gaussian 16, Revision C.01; Gaussian, Inc., Wallingford CT, **2016**.

[52] G. Luchini, J. V. Alegre-Requena, I. Funes-Ardoiz, R. S. Paton, *F1000Research* **2020**, *9*, 1–14.
